# New insights into the early morphological evolution of sea turtles by re-investigation of *Nichollsemys baieri*, a three-dimensionally preserved fossil stem chelonioid from the Campanian of Alberta, Canada

**DOI:** 10.1186/s13358-024-00323-8

**Published:** 2024-07-12

**Authors:** Juliette C. L. Menon, Donald B. Brinkman, Guilherme Hermanson, Walter G. Joyce, Serjoscha W. Evers

**Affiliations:** 1 Department of Geosciences, University of Fribourg, Chemin du Musée 6, 1700 Fribourg, Switzerland; 2 Royal Tyrrell Museum of Palaeontology, Drumheller, AB Canada; 3 Department of Biological Sciences, University of Alberta, Edmonton, AB Canada

**Keywords:** Pan-Chelonioidea, Sea turtle, CT scan, Evolution, Phylogeny, Neck retraction

## Abstract

The early evolution of Pan-Chelonioidea (sea turtles) is poorly understood. This is in part due to the rarity of undeformed skulls of definitive early stem chelonioids. In this work, we redescribe the holotype of *Nichollsemys baieri* using µCT scans and segmentations of the skull. This fossil is the best 3D preserved skull of any Campanian sea turtle, and includes partial “soft tissue” preservation. *Nichollsemys* is morphologically similar but clearly distinct from *Toxochelys* spp., and both show a mosaic of plesiomorphic and derived chelonioid features. The internal cranial anatomy documents the presence of derived characters in *Nichollsemys baieri* that are absent in *Toxochelys* spp., such as the loss of the epipterygoids and the rod-like shape of the rostrum basisphenoidale. Among the numerous plesiomorphic characters is the presence of a splenial bone, which was unnoticed before. An updated phylogenetic analysis retrieves *Nichollsemys baieri* as a non-protostegid early stem chelonioid in a slightly more crownward position than *Toxochelys latiremis*. Our phylogeny includes macrobaenids and protostegids as pan-chelonioids, and we find unorthodox results for dermochelyids. Thus, although *Nichollsemys baieri* provides important new insights into the early morphological evolution of sea turtles, much work remains to be done. As a completely 3D preserved specimen, we included *Nichollsemys baieri* into a recent landmark-based skull shape dataset of turtles. Morphospace analysis reveals an intermediate position between cryptodires and crown chelonioids. Based on these data, we also predict that *Nichollsemys baieri* was still capable of neck retraction, constraining the loss of this trait to more crownward pan-chelonioids.

**Supplementary Information:**

The online version contains supplementary material available at 10.1186/s13358-024-00323-8.

## Introduction

Among turtles, the extant sea turtles (Chelonioidea) are one of the most charismatic groups with seven living species, including the leatherback sea turtle *Dermochelys coriacea* and six species of hard-shelled sea turtles of the clade Cheloniidae. Evolutionarily, chelonioids are interesting because they represent a secondarily marine reptile group (Benson et al., 2013; Bardet et al., 2014; Evers & Benson, 2019; Hirayama, 1998; Motani, 2009; Motani & Vermeij, 2021) and because they, contrary to other Cretaceous marine reptiles, survived the Cretaceous-Paleogene mass extinction (e.g., Benson et al., 2010; Evers & Benson, 2019; Hutchison & Archibald, 1986; Parham & Pyenson, 2010)*.* Despite this, the early evolution of chelonioid sea turtles is not fully understood, which is partially due to unresolved phylogenetic questions.

Several recent phylogenetic studies produced divergence time estimates for the crown group of chelonioids based on molecular sequence data and fossil calibrations (Joyce et al., 2013; Near et al., 2005; Pereira et al., 2017; Thomson et al., 2021). These suggest that the crown group originated sometime between the early Campanian (Late Cretaceous) and the early Eocene (Ypresian), with some studies leaning toward slightly younger ages (e.g., Pereira et al., 2017) and others toward slightly older ages (e.g., Joyce et al., 2013; Thomson et al., 2021). The fossil record of marine adapted turtles is relatively good, with fossils known from the Late Jurassic up to the Recent. However, a difficulty with fossils is reliably identifying their systematic position as stem chelonioids, crown chelonioids, or not being related to chelonioids at all, which is usually done by phylogenetic inference based on morphology. Currently, there are no definitive candidates for crown chelonioids from pre-Campanian deposits, and the few fossils that have been proposed to represent potential crown members are based on very incomplete material that cannot be easily assessed by phylogenetic analysis (e.g., Cenomanian turtle humeri in Evers et al., 2019a). Thus, the fossil record can be grossly reconciled with molecular divergence estimates.

Most Early Cretaceous “sea turtle” remains are interpreted to belong to Protostegidae (e.g., Cadena & Parham, 2015; Collins, 1970; Elliott et al., 1997; Evers et al., 2019a; Hirayama, 1998; Hooks, 1998; Kear & Lee, 2006; Raselli, 2018; Scavezzoni & Fischer, 2018; Zangerl & Sloan, 1960), but it is unclear if these turtles are closely related to chelonioid sea turtles (for example, as an early stem chelonioid radiation; Gentry et al., 2019) or if they represent an independent lineage of marine turtles (see discussions in, for instance, Cadena & Parham, 2015; Evers & Benson, 2019; Gentry et al., 2019). Sediments of the Smokey Hill Chalk of the Niobrara Formation (late Coniacian to early Campanian) yielded the stratigraphically oldest definite pan-chelonioids, *Toxochelys latiremis* and *Porthochelys laticeps* (Hay, 1905; Matzke, 2008, 2009; Nicholls, 1988; Zangerl, 1953a), which are universally found as a stem chelonioids in phylogenetic matrices that otherwise do not agree on many details of chelonioid evolution (e.g., Anquetin, 2012; Anquetin et al., 2015; Cadena & Parham, 2015; Danilov & Parham, 2008; Evers & Benson, 2019; Gaffney & Meylan, 1988; Gentry et al., 2018; Joyce, 2007; Joyce et al., 2016; Sterli, 2010; Sterli et al., 2013; Zhou & Rabi, 2015). *Toxochelys* spp. are known from many specimens from the Pierre Shale Group (early–middle Campanian) and the Smokey Hill Chalk of the Niobrara (late Coniacian to early Campanian) and Mooreville Chalk (early Campanian) formations of North America (Everhart, 2017; Gentry, 2016; Hart Carrino, 2007; Liu, 2007; Nicholls et al., 1990; Walaszczyk & Cobban, 2006), which represent deposits from the Western Interior Seaway and shorelines of what now represent the Gulf of Mexico. These and other North American deposits from the Late Cretaceous also yielded a number of additional fossil sea turtles, which include forms that are similar to *Toxochelys* (e.g., *Porthochelys laticeps* from low in the Smokey Hill Chalk and thus likely from the Coniacian; Everhart, 2017; *Nichollsemys baieri* from the Bearpaw Formation and thus from the late Campanian; Ramezani et al., 2022), protostegids (e.g., *Calcarichelys gemma*, *Archelon ischyros*, *Protostega gigas*), the oldest potential crown chelonioids (Ctenochelyidae of Gentry, 2018, including *Ctenochelys* spp., *Prionochelys* spp., *Peritresius* spp.), and indeterminate sea turtle material (e.g., *Corsochelys haliniches*). Thus, although we have a rich fossil record of Late Cretaceous sea turtles, early chelonioid evolution remains poorly understood due to several issues. First, the taxonomy of many Late Cretaceous sea turtles is poorly justified by modern taxonomic standards. Second, much fossil material is either highly incomplete (e.g., *Corsochelys haliniches*), or often severely crushed, concealing much of their original anatomy (e.g., *Toxochelys latiremis*; Nicholls, 1988; Matzke, 2008, 2009). This is in contrast to the record of protostegids, for which many exquisitely preserved, three-dimensional specimens are known and described (Evers et al., 2019a; Hirayama, 1998; Kear & Lee, 2006; Raselli, 2018; Scavezzoni & Fischer, 2018).

The insufficient preservation of fossils belonging to potential stem sea turtles leads to phylogenetic uncertainty and difficulties in understanding character evolution at the origin of the group. An important exception to this type of preservation is the material described as *Nichollsemys baieri* (Brinkman et al., 2006). The type material of this turtle is from the Bearpaw Formation in Alberta, Canada, which was also part of the Western Interior Seaway during the late Campanian. The Bearpaw Formation is dated to 74.5 and 73.25 Ma (Ramezani et al., 2022) and is thus about 5 million years younger than the Sharon Springs, filling a significant gap in the North American fossil record of pan-chelonioids. This northern province of the Western Interior Seaway has a significatively scarcer fossil record for sea turtles than its older southern counterpart (Brinkman et al., 2015; Nicholls & Russell, 1990; Nicholls et al., 1990). The holotype of *Nichollsemys baieri*, TMP 1997.99.1, consists of a nearly complete, three-dimensionally-preserved skull, which represents the least deformed non-protostegid pan-chelonioid skull specimen known from the Campanian. The species was originally retrieved as a stem chelonioid, specifically as the sister taxon to *Toxochelys latiremis* (Brinkman et al., 2006). The taxon has since been largely ignored in phylogenies (e.g., Cadena & Parham, 2015; but see Brinkman et al., 2009), with only recent phylogenetic studies including it into a broad sample of chelonioids (Evers et al., 2019a; Joyce et al., 2021a). These studies retrieved *Nichollsemys baieri* within the crown along the cheloniid stem.

In this work, we used a new X-ray computed tomography (CT) scan of the holotype skull of *Nichollsemys baieri* to investigate its cranial and mandibular anatomy in order to provide an updated description. We furthermore review the phylogeny of early sea turtles based on our new observations. We show that the redescription and resulting scoring modifications have a substantial impact on chelonioid phylogeny and on the reconstruction of key features for this group. Finally, we evaluate the neck retraction capacity for this stem chelonioid turtle and provide insights into the evolution of this trait along the sea turtle lineage.

### Institutional abbreviations

AMNH: American Museum of Natural History, New York, New York, USA

FMNH: Field Museum of Natural History, Chicago, Illinois, USA

IRSNB: Institut Royal des Sciences Naturelles de Belgique, Brussels, Belgium

MHNLM: Muséum d ‘Histoire Naturelle, Le Mans (Musée Vert du Mans), France

NHMM: Natuurhistorisch Museum Maastricht, Maastricht, The Netherlands

SDSM: Museum of Geology, South Dakota School of Mines and Technology, Rapid City, South Dakota, USA

TMP: Royal Tyrrell Museum of Palaeontology, Drumheller, Alberta, Canada

UMZC: University Museum of Zoology, Cambridge, United Kingdom

USNM: Smithsonian National Museum of Natural History, Washington DC, USA

YPM: Yale Peabody Museum of Paleontology, New Haven, Connecticut, USA

## Methods

### CT-scan data acquisition

The holotype skull of *Nichollsemys baieri,* TMP 1997.99.1, was subjected to high-resolution micro-computed tomography (µCT) scanning in the mid-2000’s for the initial description of the taxon (Brinkman et al., 2006). Although these scans allow seeing many internal details (Brinkman et al., 2006), a preliminary full (i.e., bone-by-bone) segmentation of the specimen based on the original scans performed by us revealed that the scan could not resolve several sutural ambiguities that we thought to be important to understand from a systematic point of view. Thus, we produced a second CT scan of the skull of the holotype specimen, using a XT 5.4 Nikon Metrology NV device at the Permafrost Archives Science Laboratory (PACS) scanning facility of the Department of Earth and Atmospheric Sciences of the University of Alberta (Edmonton, Alberta, Canada). The scan was acquired with a beam energy of 220 kV and a beam intensity of 200 µA, resulting in an isotropic voxel size of 0.057 mm. The segmentation of the CT-scans obtained was performed manually with various thresholds and the lasso tool of the software Mimics v. 24 (http://biomedical.materialise.com/mimics). The resulting 3D models were exported as .ply files. The figures of digital renderings were performed using Blender v. 3.31 (blender.org). The new CT data as well as 3D models are deposited on MorphoSource (https://www.morphosource.org/projects/000600361).

Our segmentations revealed the potential presence of “soft tissue” preservation of the partial membranous labyrinth (see description). In order to quantitatively contrast the membranous posterior semicircular duct diameter with that of the semicircular canal, we re-sliced our Mimics file so that the posterior semicircular canal lies within one of the principal planes of the 3D coordinate system. This allows to produce a cross-section through the canal and duct that is perpendicular to the principal axis of the canal. For TMP 1997.99.1, this is relatively easy, due to the straight mid-section of the semicircular canal (see description). From this straight mid-section, we selected a centrally positioned slice and measured the cross-sectional area of the canal and duct using the Ellipse-measurement tool of Mimics (as in Evers et al., 2019b).

### Comparative material

The description and the comparison of *Nichollsemys baieri* presented here are only based on the type specimen, TMP 1997.99.1, although the original paper by Brinkman et al. (2006) refers three additional specimens to the taxon. Our detailed descriptions (below) suggest that the strong anatomical differences between the holotype and one of the referred specimens, TMP 2000.55.1, indicate that these materials represent different taxa. Consequently, we focus our descriptions on TMP 1997.99.1, the holotype of *Nichollsemys baieri*. Moreover, as no CT-scan data is available for the third specimen that consists of a sub-complete skull, SDSM 76193 (illustrated in Hart-Carrino, 2007), we also exclude this from our anatomical descriptions.

Comparative material primarily includes stem chelonioids from the Late Cretaceous (mainly Campanian), such as *Porthochelys laticeps* and *Toxochelys latiremis*, as these are coeval with and likely closely related to *Nichollsemys baieri*. The taxonomy of *Toxochelys* spp. is not fully resolved, with different authors voicing different opinions, for example on the validity or synonymy of *Toxochelys browni* (from the Pierre Shale) with *Toxochelys latiremis* (e.g., Hart Carrino, 2007; vs. Nicholls, 1988). Thus, for all mentions of *Toxochelys* spp., we either provide citations to specific literature when using specific species, or specimen numbers when the statements are based on observations that are not specifically mentioned in previous literature. We also include comparisons with more derived, but coeval chelonioid species from North American Late Cretaceous localities, for instance the putative stem cheloniid *Ctenochelys stenopora* (Santonian) or the potentially durophagous, indeterminate sea turtle *Euclastes coahuilaensis* (Late Campanian). We also include some protostegid comparisons, as protostegids may represent stem chelonioids. We include various comparisons to other fossil sea turtles as well as extant chelonioid species where relevant.

### Phylogenetic inference

#### Character-taxon matrix

To investigate the phylogenetic relationship of *Nichollsemys baieri* with other sea turtles, we used a revised version of the matrix from Joyce et al., (2021a; 356 characters for 97 taxa). This matrix was chosen because it is the most recent make-over of the dataset of Evers and Benson (2019), who provided a global turtle matrix with a specific focus on secondarily marine turtle groups, including chelonioids, and who furthermore included many new cranial character observations that were largely derived from CT-scan and 3D model observations. We did not change any character definition relative to Joyce et al. (2021a), but provide them nonetheless in Supplementary File S1. We revised the scorings for *Nichollsemys baieri* based on our new observations. As there is doubt about the taxonomic identity of other cranial specimens referred to *Nichollsemys baieri* in Brinkman et al. (2006) (see “Comparative material”, above, for further comments), the new scorings for *Nichollsemys baieri* are only based on the type specimen, TMP 1997.99.1. We rescored 28 characters for *Nichollsemys baieri* as follows: ch. 14:0- > 1; ch. 19: 1- > (1/2); ch. 26: 1- > ?; ch. 43: 0- > ?; ch. 50:?- > 1; ch. 58: 1- > ?; ch. 59:0- > 1; ch. 63:1- > 0; ch. 90:0- > 1; ch. 93:?- > 0; ch. 104:1- > 0; ch. 106:0- > 1; ch. 107:– > 1; ch. 111:1- > 0; ch. 115:?- > 0; ch. 117: 0- > 1; ch. 121:1- > 0; ch. 125:1- > 0; ch. 129:?- > 0; ch. 130:0- > 1; ch. 152:1- > 0; ch. 157:?- > 0; ch. 178:?- > 0; ch. 179:?- > 0; ch. 180:?- > 1; ch. 181:?- > 1; ch. 182:?- > 0; ch. 184:?- > 0; ch. 187:?- > 0. This resulted in a matrix in which only scorings for *Nichollsemys baieri* were changed with regard to the baseline matrix of Joyce et al. (2021a). We used this first matrix to highlight the topological changes that are entirely due to the new scorings of *Nichollsemys baieri* (see below; Supplementary file S2)*.*

We furthermore prepared a second matrix (Supplementary file S3), in which, in addition to the changes for *Nichollsemys baieri*, we also rescored some characters for other taxa when obvious mistakes became apparent in the previous scorings. These changes are: *Archelon ischyros* (ch. 57: 0- > ?), *Desmatochelys lowii* (ch. 179: 0- > ?), *Desmatochelys padillai* (ch. 179: 0- > ?)*, Eochelone brabantica* (ch. 179:0- > (0&1))*, Lepidochelys kempii* (ch. 179:0- > 1)*, Protostega gigas* (ch. 57: 0- > ?), *Rhinochelys pulchriceps* (ch. 179:0&1- > 0) and *Toxochelys latiremis* (ch. 7: 0- > ?; ch. 35: 0- > 1; ch. 41:?- > 0; ch. 69:(0&1)- > 0; ch. 82: 0- > 1; ch. 127:0- > ?; ch. 178:(0&1)- > 0; ch. 179:1- > 0; ch. 187:(0&1)- > 0)*.*

#### Parsimony analysis

All parsimony analyses were performed in TNT version 1.6 (update from 12 October 2023; Goloboff & Morales, 2023). For our analyses, we used *Proganochelys quenstedtii* as the outgroup and enforced a backbone constraint of the topology of extant taxa based on recent molecular studies (Thomson et al., 2021), as was also done previously (Evers et al., 2019a; Joyce et al., 2021a). We performed the analyses using the New Technology Search with default settings and enabled tree drifting (Goloboff, 1999) and parsimony ratchet (Nixon, 1999) algorithms. We set the initial level of driven search to 30 and the number of times the minimal tree length should be found to 30. We performed a further round of tree bisection and reconnection (TBR) to the most parsimonious trees (MPTs) previously obtained.

Initial exploratory analyses of our data as well as analyses from previous studies (Evers et al., 2019a; Joyce et al., 2021a) found poorly resolved strict consensus topologies that were caused by some highly instable rogue taxa. Thus, for all our analyses, we identified the following six taxa to be rogue using the Pruned tree comparison of TNT: *Annemys levensis, Cabindachelys landanensis, Corsochelys haliniches*, *Erquelinnesia gosseleti*, *Ordosemys* sp*.* and *Plesiochelys planiceps*. Only three of these are plausibly chelonioid sea turtles that might affect the parts of the tree that we are interested in for this study (*Cabindachelys landanensis, Corsochelys haliniches*, *Erquelinnesia gosseleti*). Of these, we choose to exclude only *Corsochelys haliniches* from subsequent analyses*,* because this taxon is based on poorly preserved material (Zangerl, 1960) that is not well described and which should be reevaluated both taxonomically and morphologically. We keep *Erquelinnesia gosseleti* because it is known by several fairly complete skulls as well as at least one incomplete individual (Zangerl, 1971) and therefore provides important information to the analysis. Its rogue nature may therefore be caused by “true” character conflict, and not be driven by incomplete knowledge of the available fossil material. In addition, we kept *Cabindachelys landanensis*, because its position was more stable once *Corsochelys haliniches* was removed.

We performed different analyses on the two matrices. The matrix in which only the scorings of *Nichollsemys baieri* were changed was analyzed in the very same manner as in previous studies (Evers et al., 2019a; Joyce et al., 2021a), i.e., all characters were left unordered and equally weighted. This was done to achieve maximal comparability with previous results. The results from this analysis are provided as Supplementary file S4 and discussed in the results section (below). However, there is good evidence that multistate characters that represent morphoclines should be ordered (e.g., Slowinski, 1993; Wilkinson, 1992). Thus, in our analysis of the second matrix we ordered the following multistate characters that form morphoclines: 7, 14, 18, 21, 34, 61, 65, 67, 74, 76, 79, 90, 93, 94, 103, 107, 117, 123, 130, 131, 138, 142, 145, 147, 205, 210, 217, 248, 253, 281, 292, 305, 325, 339, 340, 344. In addition, many turtle matrices (including the current one) have high levels of homoplasy (Evers & Benson, 2019; Evers et al., 2019a; Joyce et al., 2021a), such that implied weighting strategies may be more suited for phylogenetic analysis. Thus, we used implied weighting to reduce the impact of homoplasy on the topology of our tree, using a concavity constant of k = 12, following the recommendations of Goloboff et al. (2018). The resulting trees from this analysis are provided as Supplementary file S5 and are discussed in the results section (below).

#### Character optimization

To understand morphological evolution, it is important to understand character transitions across the topology. However, in matrices with incomplete data and character conflict between sister taxa, different end-members of character optimization, ACCTRAN (accelerated transformations) and DELTRAN (delayed transformation), often disagree so that there are ambiguous synapomorphies. We chose to use PAUP* v.4 (Swofford, 1998) for the character optimization for both analyses, because it allows one to choose manually the optimality criterion (i.e., ACCTRAN and DELTRAN), whereas TNT retains only the unambiguous synapomorphies (i.e., those synapomorphies for which ACCTRAN and DELTRAN agree). Looking at the ambiguous synapomorphies provides additional information, as the node of optimization, although not precisely known, can often be constrained by ACCTRAN and DELTRAN to be somewhere between a specific nodal arrangement in a tree. Character optimization should be performed on a fully resolved tree. To do this, we selected a random MPT of the above-mentioned TNT analyses. For the analysis with only *Nichollsemys baieri* scorings updated, we chose MPT tree number 1 from Supplementary file S4, and the resulting synapomorphy lists are deposited as Supplementary file S6. For the analysis of the fully updated matrix, we chose MPT tree number 1 from Supplementary file S5 for the optimization procedure, and deposit the synapomorphy list as Supplementary file S7.

### Prediction of neck retraction

Due to 3D preservation of the specimen, we included *Nichollsemys baieri* in the ecomorphological framework of Hermanson et al. (2022) to assess the probability of neck retraction capacity in the taxon. We used Avizo 9.0.0 (Visualization Sciences Group) to place 3D landmarks on the *Nichollsemys* type specimen following the same landmarking scheme as in the “full dataset” of Hermanson et al. (2022). We digitally rearranged both squamosals to their original position on the cranium of *Nichollsemys* to place specific squamosal landmarks and the semilandmark curve pertaining to the posterodorsal emargination. In addition, to account for potential uncertainty in this manual articulation of the squamosals, we also used a ‘reduced’ version of the landmarking scheme not including squamosal and posterodorsal emargination-related landmarks.

The analytical workflow of Hermanson et al. (2022) uses General Procrustes Analysis (GPA; Gower, 1975) and the resulting Procrustes coordinates as input for distance-based phylogenetic generalized least squares regression analysis (proc.D.pgls) in the geomorph package (Adams, 2014; Adams et al., 2020). Hermanson et al. (2022) used multiple regression models to test the effect of ecomorphology and function (including neck retraction) on skull shape. The best supported ecomorphological model included neck retraction as a significant explanatory variable. We use regression scores from this model as input data for a phylogenetic flexible discriminant analysis (pFDA; Motani & Schmitz, 2011). This analysis assesses the probability of neck retraction capacity in *Nichollsemys baieri* by calculating the mean of predicted probability values across a set of 1000 pFDA replicates. For each iteration, we randomly subset our sample to produce a training dataset with the same number of taxa in both categories of neck retraction (absent/present) to have an equal prior probability for each state (Motani & Schmitz, 2011). From the training datasets, an accuracy rate of correct predictions is generated, which provides important information for the interpretation of fossil predictions. The pFDA also requires a tree input, for which we used a pruned version of the tip-dated turtle tree of Farina et al., (2023, ‘Ev19’ tree) to match the taxa in our sample. *Nichollsemys baieri* was manually added as a stem chelonioid based on our phylogenetic results (see below; Revell, 2012). pFDA predictions are posterior probabilities, for which values over 0.5 indicate the presence of neck retraction and higher values generally indicate lower uncertainty in the prediction of the value. However, posterior probabilities should be evaluated in combination with the accuracy rates of predictions, such that also relatively low values above 0.5 can indicate the presence of a trait with high certainty when accuracy rates are very high (Fabbri et al., 2022; Hermanson et al., 2022).

We performed ancestral reconstructions of neck retraction capacity in the chelonioid lineage on our tree using the fastAnc function from the phytools package and mapped these onto the phylogeny using contMap function, also from phytools (Revell, 2012). We also ordinated the Procrustes coordinates into a Principal Component Analysis (PCA) to visualize the morphospace of turtle skull shape. All analyses were conducted in R environment (R Core Team 2020) following the script in Hermanson et al. (2022). We provide the landmark coordinates for *Nichollsemys baieri* as Supplementary file S8, the phylogenetic tree used in the analysis as Supplementary file S9, and the R script as Supplementary file S10.

## Systematic paleontology

*Testudines* Batsch, 1788

*Pan-Chelonioidea* Joyce et al., 2021b

*Nichollsemys baieri* Brinkman et al., 2006

**Fig. 1:**
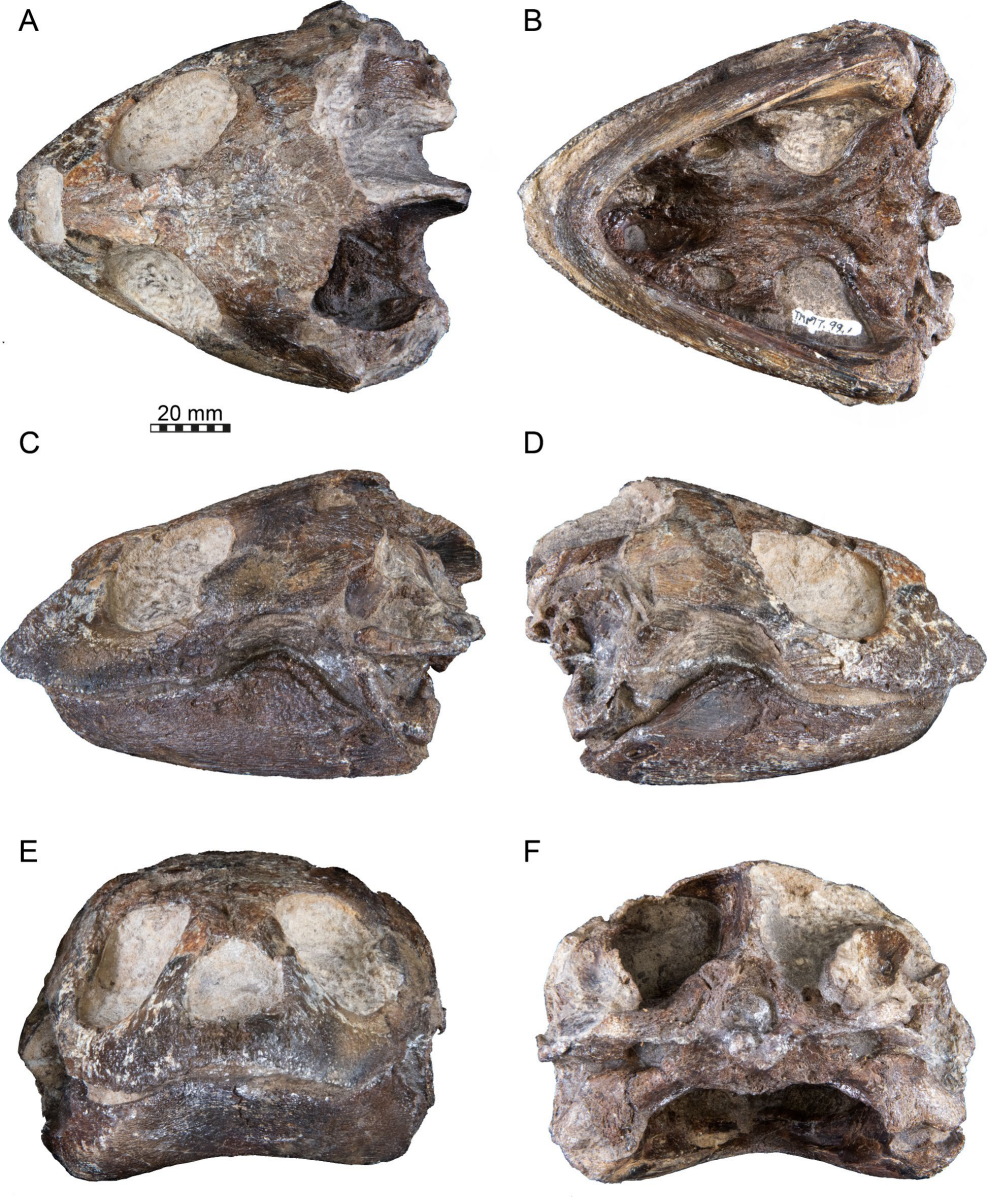
Photographs of the holotype skull of *Nichollsemys baieri* (TMP 1997.99.1). **A** dorsal view. **B** ventral view. **C** left lateral view. **D** right lateral view. **E** anterior view. **F** posterior view

Type material: TMP 1997.99.1 (holotype), a skull preserved in a concretionary nodule (Brinkman et al., 2006: Figs. 2–5) (Fig. 1).

Type locality: Along Chin Coulee, South of Taber, southern Alberta, Canada (Brinkman et al., 2006).

Type horizon: A glacial till containing concretions typical of the Bearpaw Formation, Bearpaw Shale, Late Campanian, Late Cretaceous.

### Revised diagnosis

*Nichollsemys baieri* can be identified as belonging to *Pan-Chelonioidea* by having a roughly triangular ventral exposure of the parabasisphenoid, with both ventral pterygoid ridges forming a reverse “V” pattern alongside the parabasisphenoid and basioccipital ventral exposures; a posteriorly retracted processus inferior parietalis (Gaffney, 1979); a high dorsum sellae with a distinct dorsomedian process between both clinoid processes.

*Nichollsemys baieri* shares with early protostegids (e.g., *Santanachelys gaffneyi*, *Bouliachelys suteri*, *Rhinochelys pulchriceps*) and some crown chelonioids (e.g., *Allopleuron hofmanni*) the presence of a splenial, which is well-developed with a surface area equivalent to that of the prearticular. It differs from protostegids by having a laterally closed foramen palatinum posterius; the presence of foramina praepalatina; by having a large medial jugal process contacting the palatine; and having a dorsoventral elongate foramen nervi trigemini.

*Nichollsemys baieri* shows many similarities with the stem chelonioid *Toxochelys* spp. It resembles the species by displaying the same striated ornamentation pattern of skull roof elements such as the parietal and postorbital (see Williston, 1903, pl. 18; Matzke, 2009); a robust processus pterygoideus externus terminated by a large flange; a deep temporal emargination that opens anteriorly to the foramen stapedio-temporale in dorsal view; and the presence of foramina praepalatina. However, *Nichollsemys baieri* clearly differs from *Toxochelys latiremis* and *Toxochelys moorevillensis* by showing an elongated rod-like rostrum basisphenoidale (the rostrum is flat and short in *Toxochelys)* and by lacking an epipterygoid (which is present in *Toxochelys* spp.). *Nichollsemys baieri* shares with the stem chelonioids *Toxochelys latiremis, Toxochelys moorevillensis* and *Porthochelys laticeps* a narrow interorbital bar in dorsal view, and large foramina palatinum posterius, the latter of which is likely a symplesiomorphic feature as it is present in outgroups but absent in crown chelonioids. *Nichollsemys baieri* differs from *Porthochelys laticeps* by having a much narrower cranium and narrower dentary triturating surface.

*Nichollsemys baieri* shares several features with crown chelonioids that differentiate it from protostegids, *Toxochelys* spp. and *Porthochelys laticeps*, specifically by lacking nasals. The aforementioned presence of a rod-like rostrum basisphenoidale of the parabasisphenoid is also a typical crown chelonioid feature, although it is present in at least some protostegids (e.g., *Rhinochelys pulchriceps*). It differs from many crown chelonioids (e.g., *Allopleuron hofmanni,* cheloniids) and also the indeterminate, possible crown chelonioid *Ctenochelys* spp. by lacking the inclusion of the vomer in the triturating surface and showing larger flanges on the external process of the pterygoid, and from crown chelonioids by the presence of an elongate anterior frontal process. Similarities of *Nichollsemys baieri* with the dermochelyids *Dermochelys coriacea* and *Allopleuron hofmanni* that are not shared with cheloniids include having a "T"-shaped quadratojugal. *Nichollsemys baieri* resembles *Dermochelys coriacea* by having strongly anteriorly protruding "V" shaped processes of the frontals into the prefrontals. It shares with *Allopleuron hofmanni* an anteroposteriorly very short processus inferior parietalis that contacts the crista pterygoidei posteriorly to the anterior end of the basisphenoid; the presence of a large fenestra from the internal carotid artery canal into the canalis cavernosus; and a deep cheek emargination that reaches the height of the ventral third of the orbit. *Nichollsemys baieri* differs from *Dermochelys coriacea* by lacking tooth-like processes along the cranial labial ridge and by the presence of the coronoid. It differs from *Dermochelys coriacea* and *Allopleuron hofmanni* by lacking an extensive contact between jugal and squamosal and showing a postorbital-quadratojugal contact.

*Nichollsemys baieri* is unique among pan-chelonioids by having by a very small ventral exposure of the parabasisphenoid, a strongly reduced sella turcica, and a dorsally notched labial margin in the symphysis of the dentary.

## Description and comparisons

### General appearance

**Fig. 2:**
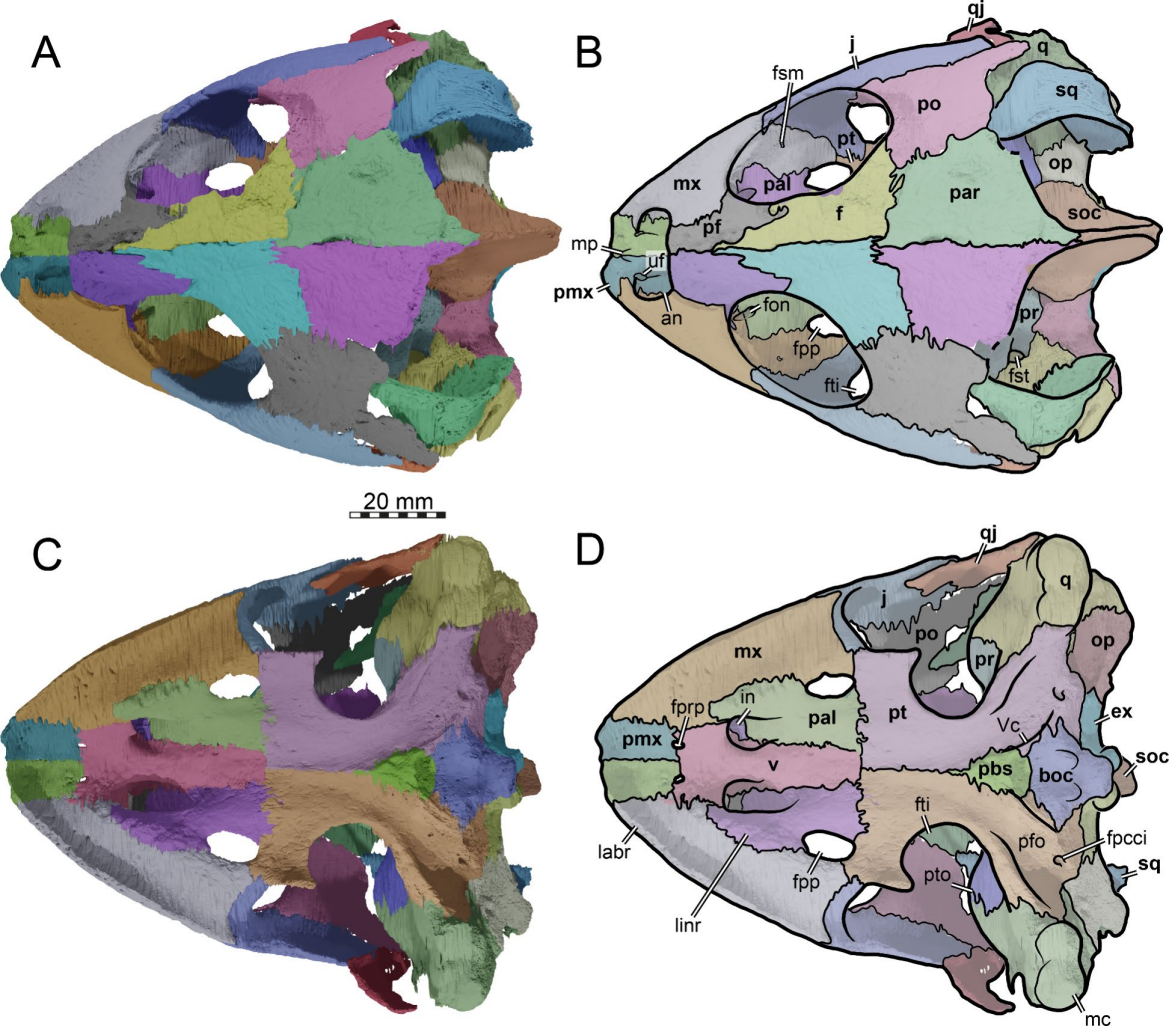
Three-dimensional renderings of the cranium of the holotype specimen of *Nichollsemys baieri* (TMP 1997.99.1). **A** dorsal view; **B** interpretative line-drawing; **C** ventral view; **D** interpretative line-drawing. Note that bones are labelled in bold and features labelled in regular font. *an* apertura naris, *boc* basioccipital, *ex* exoccipital, *f* frontal, *fon* foramen orbito-nasale, *fpcci* foramen posterius canalis caroticus interni, *fpp* foramen palatinum posterius, *fprp* foramen praepalatinum, *fsm* foramen supramaxillare, *fst* foramen stapedio-temporale, *fti* inferior temporal fossa, *in* internal nares, *j* jugal, *labr* labial ridge, *linr* lingual ridge, *mc* mandibular condyle, *mx* maxilla, *mp* median pocket, *op* opisthotic, *pal* palatine, *par* parietal, *pbs* parabasisphenoid, *pf* prefrontal, *pfo* pterygoid fossa, *pmx* premaxilla, *po* postorbital, *pr* prootic, *pt* pterygoid, *pto* processus trochlearis oticum, *q* quadrate, *qj* quadratojugal, *soc* supraoccipital, *sq* squamosal, *uf* unnamed foramen,* v* vomer, *Vc “*V” shaped ventral crest

The skull of TMP 1997.99.1 is fairly complete, with nearly the entire cranium and the mandible being preserved (Fig. 1; Brinkman et al., 2006). Most sutures are clear in the slice data so that we could produce a full digital reconstruction of each cranial bone (Fig. 2). The posterior part of the parietal and the crista supraoccipitalis are broken. The squamosals are not articulated anymore and still floating in the matrix surrounding the upper temporal emargination (see below). On the right side, the posterior part of the jugal and postorbital bones are not preserved. In addition, most of the right quadratojugal is not preserved. Only a fragment of it remains in disarticulation still floating in the matrix. The cranium has a length of 111.5 mm from the tip of the snout to the base of the occipital condyle and a maximum width of 96.5 mm between the quadratojugals, as measured with the straight-line measurement tool of Mimics. Cranial scales sulci are not visible despite good preservation of the external surface on the skull roof that bears a radiated ornamentation pattern (Brinkman et al., 2006) similar to the condition found in *Toxochelys latiremis* (Fig. 1; Williston, 1903: pl. 18). TMP 1997.99.1 shows potential internal “soft tissue” preservation, particularly the preservation of a semicircular duct (see below). The snout is slightly elongated but blunt. In dorsal view, the interorbital bar is narrow like in *Toxochelys latiremis* (Matzke, 2009) and the orbits are dorsolaterally oriented (Fig. 1A). The maxilla shows a slightly grainy exterior surface below the ascending process, possibly indicating the extent of the rhamphotheca. The cheek emargination, which reaches the lower third of the orbit, is deep for a marine turtle. The braincase and the otic region are well preserved (see below). There is no evidence for the presence of ossified epipterygoids, as any corresponding sutures that would indicate their presence are not visible, even though all other sutures are easily discerned. The specimen has a pair of small and anteroposteriorly elongated foramina praepalatina, a pair of large and anteroposteriorly elongated foramina palatinum posterius on the palate (Brinkman et al., 2006), and a pair of large and rounded foramina orbito-nasale between the orbit and the nasal cavity. A secondary palate is absent (Fig. 1). The mandible is almost completely preserved, showing a small abraded area at its posterior left end. The anterior tip of the dentary shows unusual symmetrical notch (see below). The mandible is still in articulation with the cranium (Fig. 1).

### Nasal

**Fig. 3:**
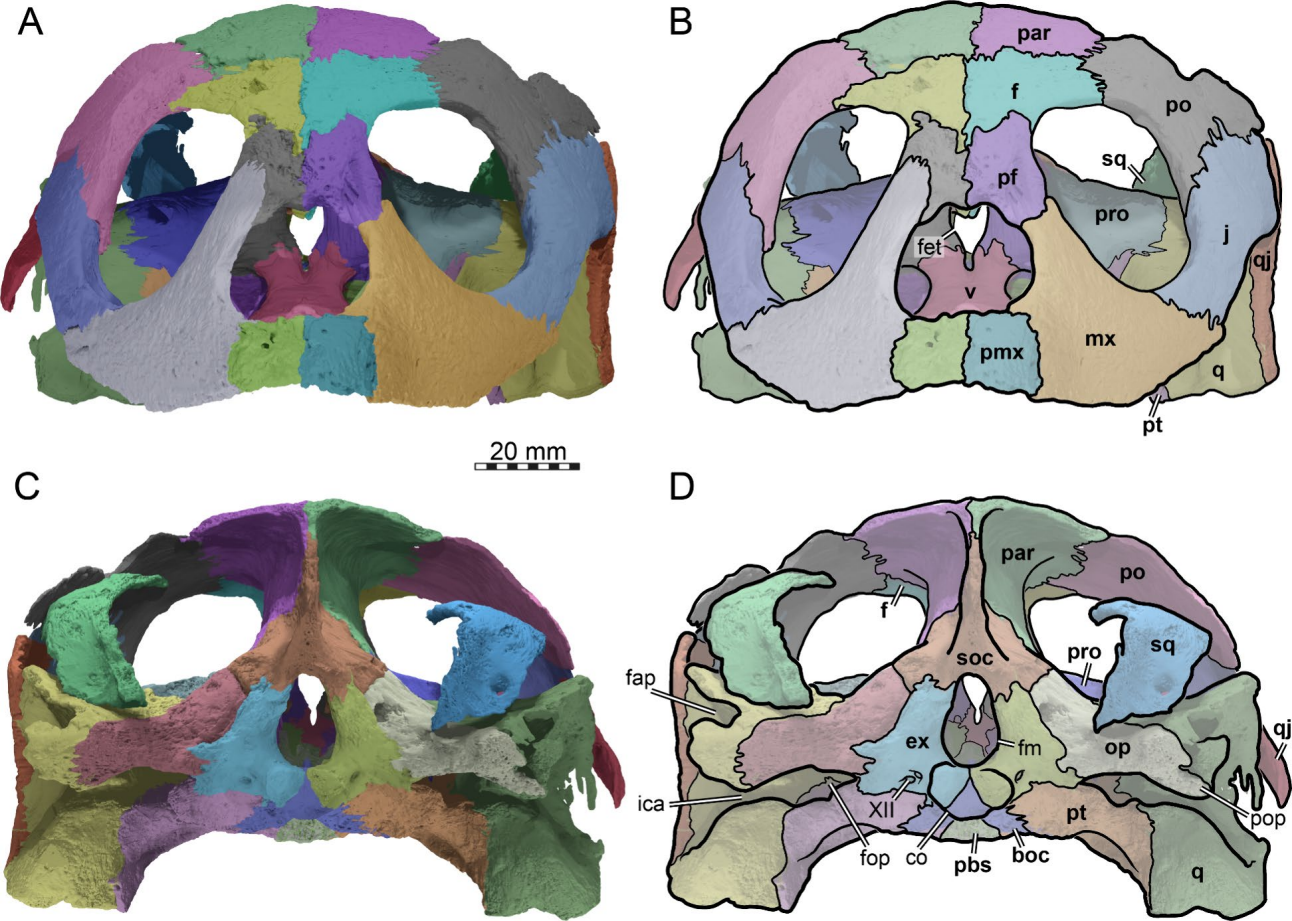
Three-dimensional renderings of the cranium of the holotype specimen of *Nichollsemys baieri* (TMP 1997.99.1). **A** anterior view; **B** interpretative line-drawing; **C** posterior view; **D** interpretative line-drawing. Note that bones are labelled in bold and features labelled in regular font. *an* apertura naris, *boc* basioccipital, *ex* exoccipital,* f* frontal, *fap* foramen antrum postoticum, *fet* fissura ethmoidalis, *XII* foramen nervi hypoglossi, *fm* foramen magnum, *fop* fenestra postotica, *ica* incisura columella auris,* j* jugal, *mx* maxilla, *op* opisthotic, *pal* palatine, *par* parietal, *pbs* parabasisphenoid, *pf* prefrontal, *pmx* premaxilla, *po* postorbital, *pop* paraoccipital process, *pr* prootic, *pt* pterygoid, *qj* quadratojugal, *q* quadrate, *soc* supraoccipital, *sq* squamosal, *v* vomer

The dorsal margins of the external nares of TMP 1997.99.1 are fully intact (Figs. 2, 3). It is therefore apparent that nasals are absent (Brinkman et al., 2006). The absence of nasals contrasts with the condition found in the stem chelonioids *Toxochelys latiremis* and *Porthochelys laticeps* (Matzke, 2009; Zangerl, 1953a) and early protostegids (Evers et al., 2019a; Hooks, 1998; Kear & Lee, 2006; Raselli, 2018).

### Prefrontal

**Fig. 4:**
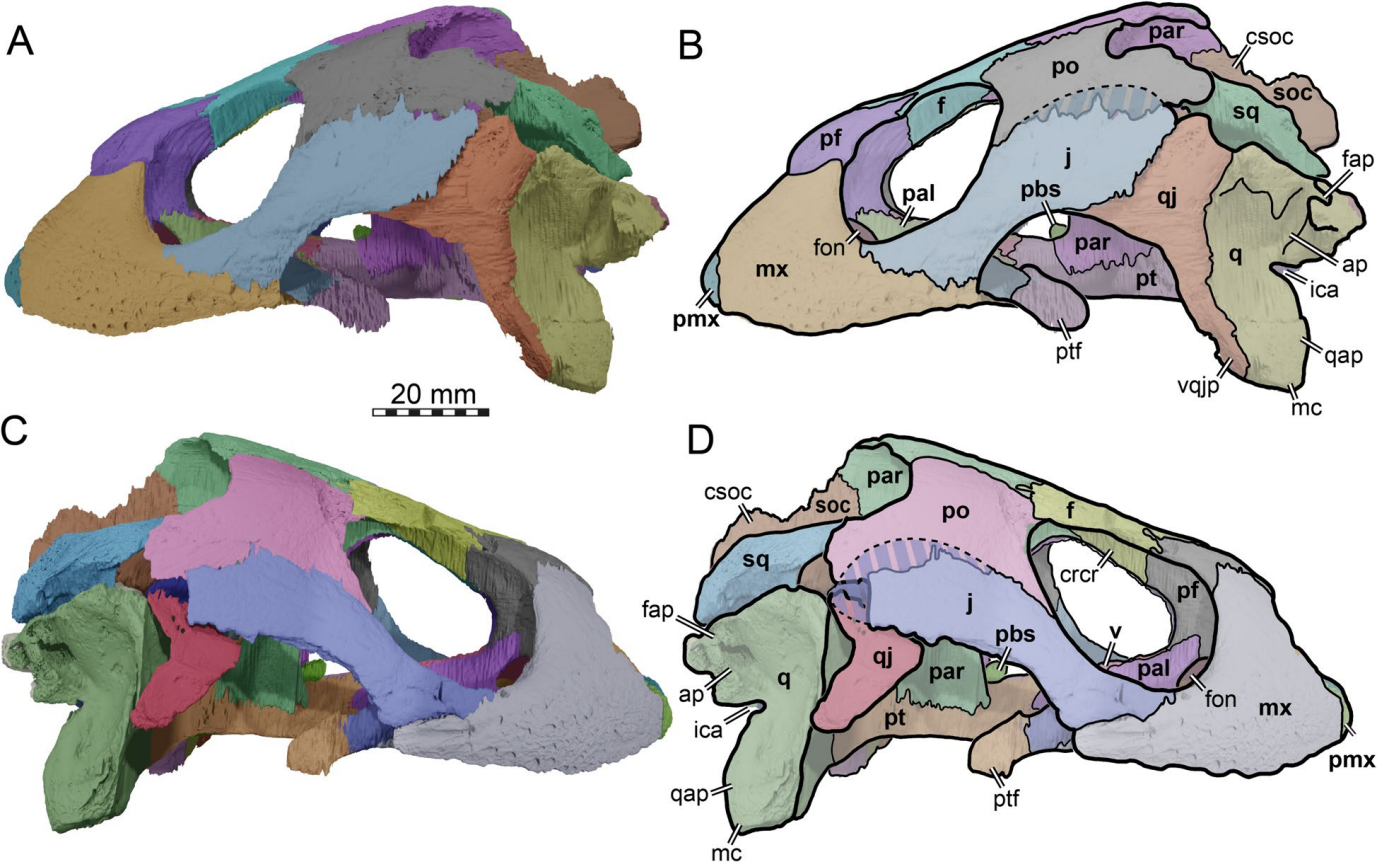
Three-dimensional renderings of the cranium of the holotype specimen of *Nichollsemys baieri* (TMP 1997.99.1). **A** left lateral view; **B** interpretative line-drawing; **C** right lateral view; **D** interpretative line-drawing. Note that bones are labelled in bold and features labelled in regular font. Also note that on the right skull side, the jugal facets on the postorbital and quadratojugal indicate an originally larger extent of the bone, indicated in D by hashed color and a dashed outline. *ap* antrum postoticum, *crcr* crista cranii, *csoc* crista supraoccipitalis, *f* frontal, *fap* foramen antrum postoticum, *fon* foramen orbito-nasale, *ica* incisura columella auris, *j* jugal, *mc* mandibular condyle, *mx* maxilla, *pal* palatine, *par* parietal, *pbs* parabasisphenoid, *pf* prefrontal, *pmx* premaxilla, *po* postorbital, *pt* pterygoid, *ptf* pterygoid flange, *q* quadrate, *qap* quadrate articular process, *qj* quadratojugal, *soc* supraoccipital, *sq* squamosal, *v* vomer, *vqjp* ventral quadratojugal process

The prefrontals of TMP 1997.99.1 are fairly complete (Figs. 2, 3, 4). The posterior part of the dorsal surface of the right prefrontal is damaged at the articulation with the frontal along the orbit. In addition, the anterolateral border of the right prefrontal is damaged at the suture with the maxilla. Nevertheless, the full morphology of the prefrontal can be appreciated. The prefrontal contacts the maxilla laterally, the frontal posteriorly, its counterpart medially, and the descending process contacts the vomer and the palatines posteroventrally (Figs. 2, 3, 4). The prefrontal forms the dorsal border of the foramen orbito-nasale and participates anterodorsally in the orbital margin (Fig. 4). The prefrontal forms the dorsal border of the external nares (Fig. 2A, B). The prefrontals jointly contribute to the anterior two thirds of the interorbital bar. In dorsal view, the prefrontals are elongated but narrower than in moderns cheloniids (Fig. 2A, B). A narrow interorbital bar occurs in *Toxochelys latiremis* (Matzke, 2009), *Porthochelys laticeps* (Williston, 1903), and in *Mexichelys coahuilaensis* (Brinkman et al., 2009)*.* The medial suture between both prefrontals of TMP 1997.99.1 is less than half of the total length of these bones, as a full midline contact is hindered by a deep median process formed by the frontals (Fig. 2A, B). The contribution of the prefrontal to the external naris is greatly reduced in comparison to other chelonioids without nasals, in which the prefrontal often forms a small part of the lateral margin of the external naris. The prefrontal of TMP 1997.99.1 forms the lateral margin of the keyhole shaped fissura ethmoidalis (Fig. 3A, B).

### Frontal

**Fig. 5:**
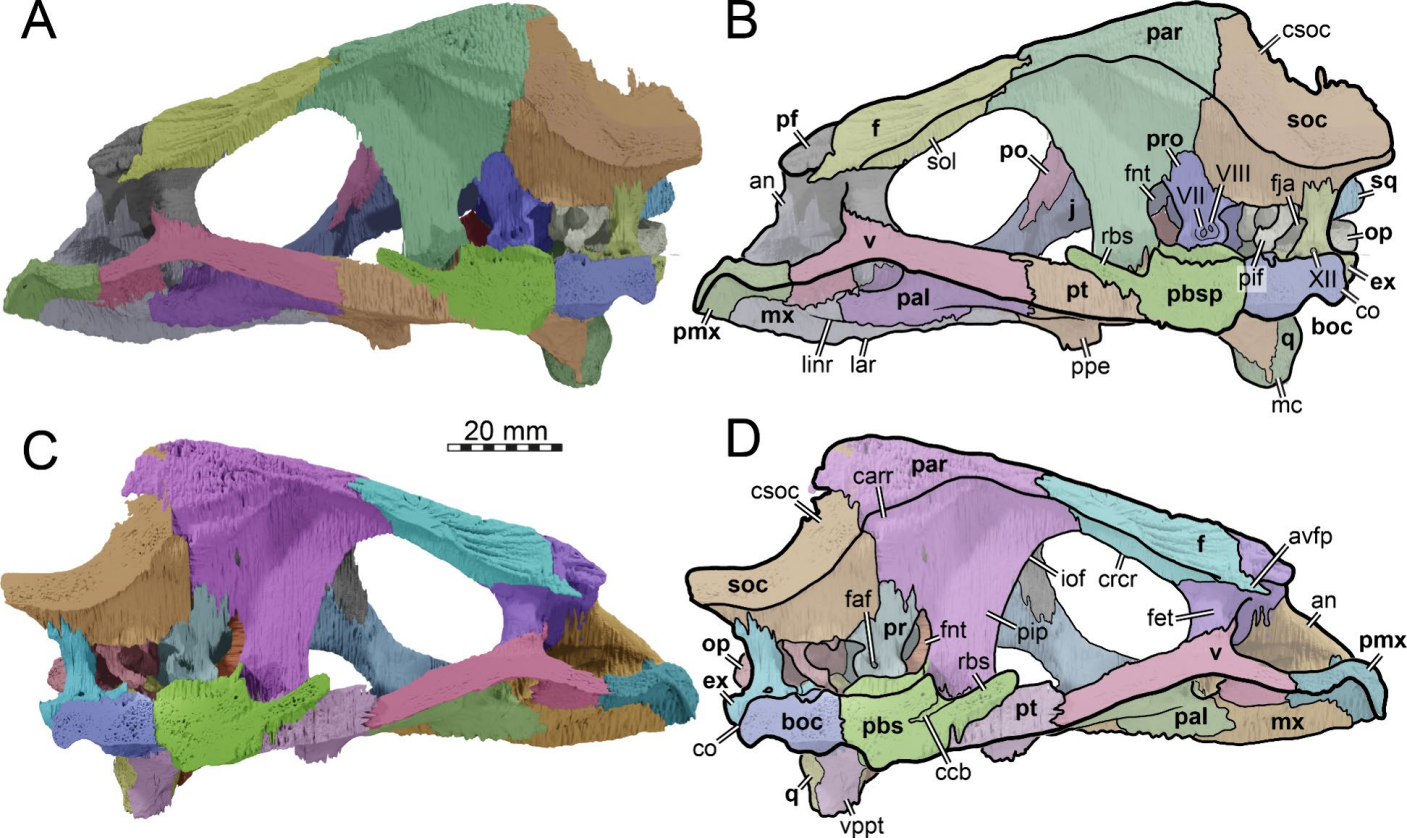
Three-dimensional renderings of the cranium of the holotype specimen of *Nichollsemys baieri* (TMP 1997.99.1). **A** right internal view; **B** interpretative line-drawing; **C** left internal view; **D** interpretative line-drawing. Note that bones are labelled in bold and features labelled in regular font. *an* apertura naris, *avfp* anteroventral process of the frontal, *boc* basioccipital, *carr* cartilaginous rider, *ccb* canalis caroticus cerebralis, *co* occipital condyle, *csoc* crista supraoccipitalis, *ex* exoccipital, *f* frontal, *faf* fossa acustico-facialis, *fet* fissura ethmoidalis, *fnt* foramen nervi trigemini, *fon* foramen orbito-nasale, *iof* interorbital fenestra, *j* jugal, *mc* mandibular condyle, *mx* maxilla, *op* opisthotic, *pal* palatine, *par* parietal, *pbs* parabasisphenoid, *pf* prefrontal, *pif* processus interfenestralis, *pip* processus inferior parietalis, *pmx* premaxilla, *po* postorbital, *pr* prootic, *pt* pterygoid, *qj* quadratojugal, *q* quadrate, *rbs* rostrum basisphenoidale, *soc* supraoccipital, *sol* sulcus olfactorius, *sq* squamosal, *XII* foramen nervi hypoglossi,* v* vomer, *VII* foramen nervi facialis, *VIII* foramen nervi acusticus, *vppt* ventral process of the pterygoid

The left frontal of TMP 1997.99.1 is completely preserved but the right one is damaged near the orbit (Figs. 2, 3, 4, 5). The frontal participation to the skull roof is just slightly smaller than that of the parietal (Fig. 2A, B), in contrast to modern chelonioids that exhibit a significantly larger parietal contribution than their frontal one. The frontal of TMP 1997.99.1 contacts the prefrontal anteriorly, meets the postorbitals posterolaterally and the parietals posteriorly, and its counterpart at the midline (Fig. 2A, B). The frontal laterally makes an important contribution to the orbit about equal in size to that of the postorbital (Figs. 2, 3, 4). This is similar to the early protostegid *Rhinochelys pulchriceps* and stem chelonioids *Toxochelys latiremis* (Matzke, 2009) and *Porthochelys laticeps*, but different to the frontal contribution to the orbit in cheloniids, which is often small (e.g., Chatterji et al., 2021; Jones et al., 2012). The frontals of TMP 1997.99.1 jointly form a pointed anterior process that deeply inserts between the prefrontals (Fig. 5). The process is longer ventrally than dorsally, such that the frontal deeply underlaps the prefrontal and extends through the fissura ethmoidalis into the nasal capsule. The pointed anterior process is longer and narrower than in other chelonioids. On the ventral surface of the frontal of TMP 1997.99.1, low and blunt ridges, the crista cranii, are present that anteriorly connect to the fissura ethmoidalis and posteriorly to the descending process of the parietal (Figs. 4, 5). The crista cranii form the sulcus olfactorius, a ventrally open trough that allows the transmission of the olfactory nerve from the braincase to the nasal capsule (e.g., Evers et al., 2019b; Ferreira et al., 2022; Gaffney, 1979). The crista cranii of *Nichollsemys baieri* resemble those of most pan-chelonioids, including *Toxochelys latiremis* (USNM 11558), but are higher than those of *Dermochelys coriacea* and lack the midline contact that is seen in some early protostegids (Evers et al., 2019a).

### Parietal

The parietals of TMP 1997.99.1 are generally well preserved, but their posterior margins show signs of damage, particularly the left one (Figs. 2, 4, 5). As preserved, the parietals initially suggest the presence of deep upper temporal emarginations that expose the foramen stapedio-temporale on the otic process in dorsal view (Fig. 2A, B), but the entire bone margin, and the articulation with the supraoccipital, is too damaged to document the depth of the emargination with certainty. However, despite these damaged margins of the parietal, there is good evidence for deep posterior emarginations from the squamosals, which are completely preserved but disarticulated (see below). The dorsal margin of the squamosal is strongly anteromedially curved, as is only the case in taxa with relatively deep emarginations. Thus, we interpret that the damage along the posterior parietal margins is only relatively superficial and that the posterior emargination reached beyond the level of the foramen stapedio-temporale, as also is the case in *Toxochelys latiremis* (Matzke, 2009; see the description of the squamosal below for a more detailed justification). This greatly contrasts with the condition of TMP 2000.55.1, another skull referred to *Nichollsemys baieri* by Brinkman et al., (2006: Fig. 3), which seems to lack any trace of temporal emarginations and which is one of the reasons why we believe this specimen to belong to a different taxon. We therefore here and elsewhere focus on the descriptions of TMP 1997.99.1, the holotype of *Nichollsemys baieri*, and do not consider TMP 2000.55.1 as part of the paradigm for *Nichollsemys baieri*.

**Fig. 6:**
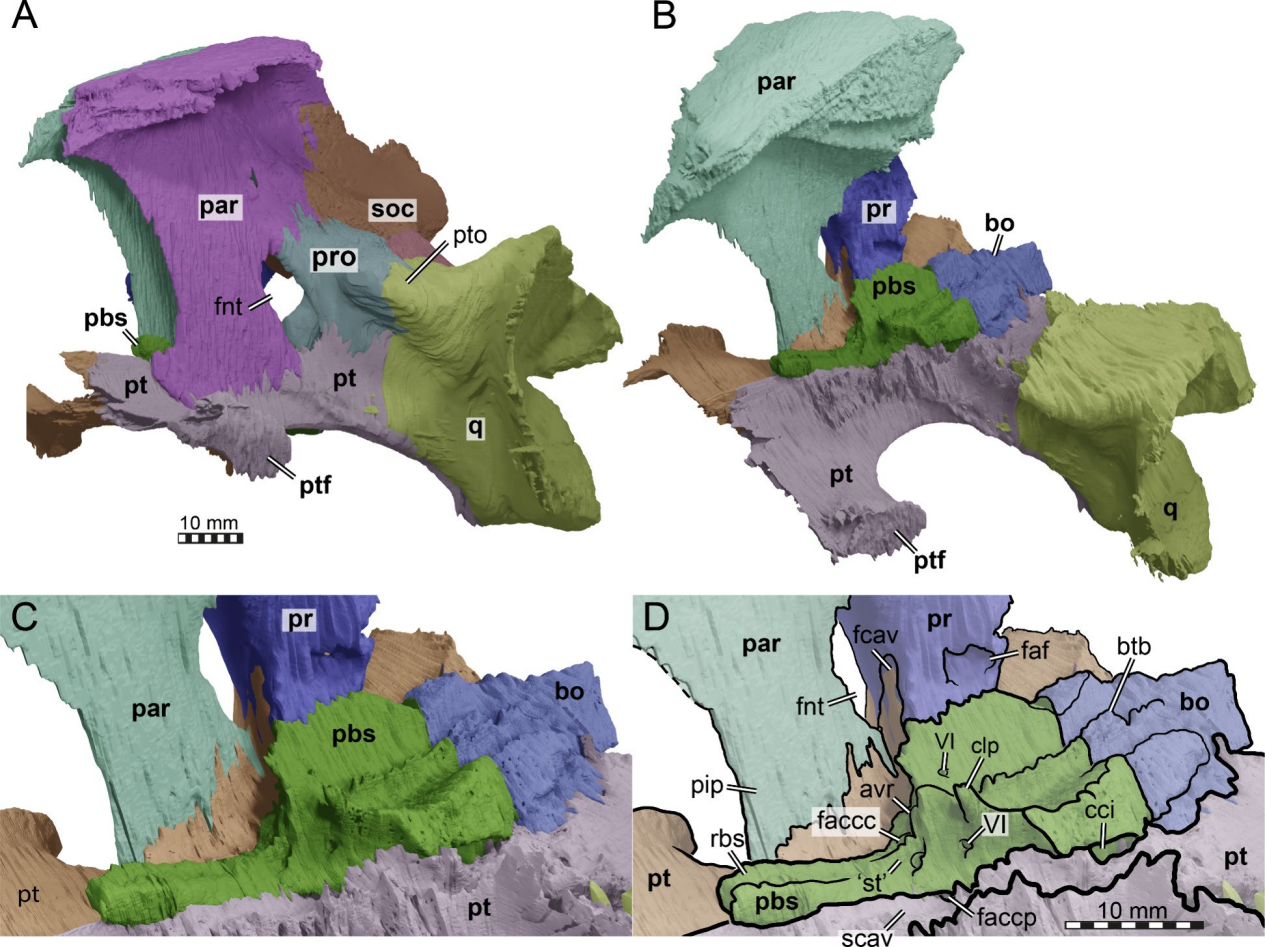
Three-dimensional renderings of the partial braincase of the holotype specimen of *Nichollsemys baieri* (TMP 1997.99.1), **A** anterolateral view onto the trigeminal area of the left skull side; **B** anterolateral view of the internal basicranium; **C** close-up on the parabasisphenoid area based on B; **D** interpretative line-drawing. Note that bones are labelled in bold and features labelled in regular font. *avr* anterior ventral ridge, *boc* basioccipital, *btb* basis tuberculum basalis, *cci* canalis caroticus internus, *clp* clinoid process, *faccc* foramen anterius canalis caroticus cerebralis, *faccp* foramen anterius canalis carotici palatinum, *faf* fossa acustico-facialis, *fcav* foramen cavernosum, *fnt* foramen nervi trigemini, *par* parietal, *pbs* parasphenoid, *pip* processus inferior parietalis, *pr* prootic, *pt* pterygoid, *q* quadrate, *rbs* rostrum basisphenoidale, *scav* sulcus cavernosus, *soc* supraoccipital, *‘st'* vestigial sella turcica, *VI* foramen nervi abducentis

The parietal of TMP 1997.99.1 is a large bone that is constituted of two plates (Figs. 2, 4, 5). The horizontal plate contributes to the dorsal skull roofing and the upper temporal emargination, while the descending plate forms the processus inferior parietalis (Figs. 5, 6). Anteriorly, the horizontal plate contacts the frontal along an oblique suture, which traverses posterolaterally from the skull midline to the triple junction with the postorbital (Fig. 2A, B). The parietal contacts the postorbital laterally and meets the other parietal medially alongside the interparietal suture (Fig. 2A, B). Due to the disarticulation of the squamosals and to the damaged posterior margins of the postorbitals and parietals, it is not possible to determine with certainty if the parietal contacted the squamosals posterolaterally. However, when the squamosals are rotated back into what we believe to be their original position, a contact is absent by a short distance (see squamosal, below). Nevertheless, we cannot exclude that this contact was present, as the probably closely related stem chelonioid *Toxochelys latiremis* has a peculiar posterolaterally directed parietal process that extends along the posterior postorbital margin (Matzke, 2009), thereby excluding the latter from the temporal emargination. In *Toxochelys latiremis*, this process establishes a contact with the squamosal (e.g., Matzke, 2009), but the broken margin of the parietal in TMP 1997.99.1 does not allow us to verify if this process may have been present. The horizontal plate of the parietal of TMP 1997.99.1 is short, roughly trapezoidal, with its longest edge forming the interparietal suture (Fig. 2A, B). A pineal foramen is clearly absent. However, fine striations that dorsally cover the parietals radiate from the midpoint of the interparietal suture (Fig. 1A). The parietals fully overlap the supraoccipital in the skull roof for their preserved posterior length (Fig. 2A, B). Anterior to the supraoccipital, the parietals are ventrally strongly constricted inside the cavum cranii, forming a deep, midline trough (Figs. 5, 6). In an endocast of the braincase, this would show as a positively protruding, dorsal medial ridge, the ‘cartilaginous rider’ of Zangerl (1960) (Fig. 5), which has shown to be a space for the anterior, cartilaginous parts of the supraoccipital (Werneburg et al., 2021).

The processus inferior parietalis (Figs. 5, 6) forms nearly the entire secondary lateral braincase wall. The processus inferior parietalis has a part anterior to the trigeminal foramen, which extends to the pterygoid ventrally (Figs. 5, 6). In most turtles, including extant chelonioids, the processus inferior parietalis articulates with the pterygoid along a ridge called the crista pterygoidei, which is a dorsally ascending plate of the pterygoid that also forms parts of the secondary lateral braincase wall. In TMP 1997.99.1, the crista pterygoidei is extremely low (Fig. 6), such that the parietal directly contacts the dorsal surface of the central part of the pterygoid. The processus inferior parietalis anterior to the trigeminal foramen is anteroposteriorly short, creating a large foramen interorbitale (Fig. 5), as in all chelonioids except *Dermochelys coriacea*, which lacks an osseous processus inferior parietalis completely. The anterior part of the processus inferior parietalis is mediolaterally thin and its lateral surface bears no ridge (Fig. 6B). This contrasts with extant chelonioids, in which the lateral surface of this part of the processus inferior parietals is laterally thickened. The anterior part of the processus inferior parietalis of TMP 1997.99.1 also forms the anterior margin of the large, ovoid, and almost vertically oriented trigeminal foramen (Fig. 6B). The posterior rim of the foramen is formed by the prootic, the ventral rim by the pterygoid, as in protostegids and chelonioids (Chatterji et al., 2021; Evers et al., 2019a; Jones et al., 2012; Raselli, 2018). A posteroventral process of the parietal along the posterodorsal margin of the trigeminal foramen is absent in TMP 1997.99.1 (Fig. 6B). Posterodorsal to the trigeminal foramen, the processus inferior parietalis overlaps the prootic and supraoccipital (Fig. 6B).

### Postorbital

The posterior part of the postorbitals is difficult to differentiate from the jugals in the CT scans, due to the shingled nature of the contact of these bones. However, the suture lines are quite distinct in external view of the specimen (Fig. 1C, D), in part due to the striations on both bones, which have different trajectories. Thus, we tried to find and model the sutures in the CT scan along the expected sutural trajectory from the external specimen’s view.

The postorbital of TMP 1997.99.1 (Figs. 2, 3, 4) is an elongated, roughly rectangular bone (Fig. 2A, B). Its surface is sculptured by fine striations that originate from a point just behind the orbit (Fig. 1C, D). It contributes to the skull roof, from the posterior rim of the orbit to the anterior rim of the squamosal (Figs. 2, 3, 4). The postorbital contacts the jugal anterolaterally, the frontal anteromedially, the parietal posteromedially, and the squamosal posteriorly (Figs. 2, 3, 4). A contribution to the rim of the upper temporal emargination is not preserved, but it likely existed as the squamosal probably lacked a parietal contact (see parietal and squamosal). A small gap separates the ventral margin of the posterior process of the postorbital and the dorsal margin of the quadratojugal on both skull sides (Fig. 4), but the edges of these bones all appear slightly broken or eroded. It is possible, and maybe even likely, that the postorbital–quadratojugal contact was originally present. However, it is also possible that this contact, if existent, was limited to the internal side of the cheek, and not expressed on the lateral skull surface. This would suggest a small jugal–squamosal contact, which is potentially indicated on the right side of the skull by a shallow facet on the ventral part of the posterior process of the postorbital, which seems to indicate the former extent of the now broken right jugal. The postorbital contributes to the posterior orbital margin and constitutes approximatively one quarter of the orbital perimeter. The orbital margin formed by the postorbital is well rounded (Figs. 2, 3, 4) and lacks an “eyebrow” process that is seen in many extant chelonioids (e.g., *Lepidochelys olivacea*: SMNS 11070; *Chelonia mydas*: NHMUK 1969.766). The posterior margin of the orbit is slightly thickened on the visceral side of the postorbital (Fig. 3A, B).

### Jugal

The jugals are intact with the exception of damage to the posterior process of both bones (Figs. 2, 3, 4). The jugal of TMP 1997.99.1 is an elongate bone that forms much of the cheek region (Fig. 4). It externally contacts the maxilla anteroventrally, the postorbital dorsally, and the quadratojugal posteroventrally (Fig. 4). We are unable to establish a clear contact between the quadratojugal and postorbital despite the general articulation and exquisite preservation of the skull (see postorbital above). If this absence of contact is genuine, then the squamosal and jugal must have had a small contact. A squamosal–jugal contact is rare among turtles, but typically occurs in dermochelyids, including the probable fossil dermochelyid *Allopleuron hofmanni* (Mulder, 2003). Its potential presence in TMP 1997.99.1 may thus have systematic relevance. However, the squamosal–jugal contact in dermochelyids is at least partially formed by an anteroventral process of the squamosal, which inserts anteriorly between the quadratojugal and postorbital along the lateral skull surface. Such a process is clearly absent in the well-preserved squamosals of TMP 1997.99.1 (see below). Thus, even if a small squamosal–jugal contact was originally present, this may not be homologous with the dermochelyid condition. Although the contact is usually absent in stem chelonioids such as *Toxochelys latiremis* (Matzke, 2009) and extant cheloniids, it can occur in some extant specimens as individual variation (e.g., *Caretta caretta*: MHNLM EMV 2004.3.22; Supplementary file S1: Fig. S1).

The anterior half of the jugal of TMP 1997.99.1 forms a narrow jugal arch posteroventral to the orbit (Fig. 4). This arch participates in the cheek emargination ventrally and forms the posteroventral orbital rim dorsally. The jugal participation to the orbital rim is about one quarter of the total perimeter (Fig. 4) and thus more extensive than in extant cheloniids and *Dermochelys coriacea* (Chatterji et al., 2021, 2022; Gaffney, 1979; Jones et al., 2012; Seago, 1979), which already have comparatively large jugals. In the stem chelonioid *Toxochelys latiremis*, the jugal participation to the orbit is much smaller (Matzke, 2009), and more similar to non-chelonioid turtles. The ventral rim of the jugal arch of TMP 1997.99.1 forms the anterior two thirds of the cheek emargination (Fig. 4), which is moderately deep in comparison to most turtles generally, but notably deep for a marine taxon more widely, or pan-chelonioids specifically. The cheek emargination of TMP 1997.99.1 stretches from the posterior end of the jugal-maxilla contact to the posterior end of the quadratojugal, forming a wide concave margin that extends dorsally to the level of the lower third of the orbit. This condition is similar to *Toxochelys latiremis* (see Matzke, 2009: Figs. 3, 4) and somewhat deeper than in dermochelyids, including *Allopleuron hoffmanni* (Mulder, 2003) and *Dermochelys coriacea.*

Medially, the jugal of TMP 1997.99.1 is extended by a broad and hooked-shaped medial process (Fig. 2C, D). This medial process is large, which is typical for most turtles, contacts the maxilla anteriorly, has a broad contact with the pterygoid posteriorly (Fig. 2C, D), but a contact with the palatine is clearly absent. The later contact is prevented both by the presence of a large foramen palatinum posterius and a pterygoid–maxilla contact along the posterior margin of this foramen (Fig. 2C, D). These contacts are basically identical to the morphology of *Toxochelys latiremis* (AMNH 5118). This can even be appreciated by the figures of Matzke (2009: Fig. 2a, b), although the author attests the contact to be present, citing their reconstruction drawing as evidence (Matzke, 2009: Fig. 22). The presence of a plesiomorphically large medial process of the jugal in *Toxochelys latiremis* and TMP 1997.99.1 contrasts with the condition in cheloniids, in which this process is generally less developed than in TPM 1997.99.1 and in which a contact with the palatine is present, due to the absence of a foramen palatinum posterius. In protostegids and dermochelyids, the medial process of the jugal is entirely reduced (e.g., Evers & Benson, 2019) and its absence prevents the possibility of the jugal contacting the palatine or pterygoid.

### Quadratojugal

The right quadratojugal of TMP 1997.99.1 is poorly preserved (Fig. 4C, D). Only a little fragment is preserved floating in the matrix. The left quadratojugal, by contrast, is essentially complete (Fig. 4A, B). The quadratojugal of TMP 1997.99.1 is situated in the posterior region of the cheek area and participates in the posterior half of the cheek emargination (Figs. 2, 3, 4). The bone is composed of two principal parts, an expanded dorsal plate that forms parts of the temporal region and an elongated, ventrally descending process (Fig. 4). The dorsally expanded part is anterolaterally overlapped by the jugal. Posterior to the jugal contact, the quadratojugal certainly contacted the disarticulated squamosal, but a contact with the postorbital dorsally is less clear (Fig. 4). This contact is not preserved on neither side of the skull, making it possible that this represents a genuine absence. In this case, a squamosal–jugal contact would be present instead. In *Allopleuron hofmanni* and *Dermochelys coriacea*, the quadratojugal is excluded from contacting the postorbital by an extensive contact between the squamosal and the jugal (Mulder, 2003). In addition, the quadratojugal of TMP 1997.99.1 contacts the quadrate along its descending process, which overlays the quadrate laterally (Fig. 3A, B), thereby forming the anterolateral margin of the cavum tympani (Fig. 4). The vertical ventral quadratojugal process of TMP 1997.99.1 is rod-shaped, resulting in an overall “T-shaped” quadratojugal that is also found in *Allopleuron hofmanni* (Mulder, 2003) and *Dermochelys coriacea* (Gaffney, 1979; Nick, 1912; Seago, 1979), which contrasts with the reconstruction of the ventral quadratojugal process of the original description (Brinkman et al., 2006). This shape is due to the absence of the anterior prolongation of the anterior part of the quadratojugal that occurs in cheloniids and protostegids, and which gives a “L” shape to this bone. The deep ventral process of the quadratojugal also contributes to the cheek emargination (Fig. 4), which is generally deeper than in most other chelonioids. Despite the similarity in the “T-shape”, the quadratojugal of TMP 1997.99.1 differs from those of dermochelyids in being less clearly integrated into the cavum tympani. Although it certainly forms its anterior margin, it does not extend medially to form parts of the internal, anterior surface of the cavum tympani, as is the case in *Allopleuron hofmanni* (NHMUK R4213) and *Dermochelys coriacea* (UMZC R3031). All *Toxochelys latiremis* specimens that preserve the quadratojugal are severely crushed, making it difficult to fully interpret this morphology (e.g., Matzke, 2009). However, it seems possible that its original shape was similar to that of *Nichollsemys baieri*, based on our examinations of 3D models of AMNH 5118. This specimen preserves a thin ventral quadratojugal process as in TMP 1997.99.1. Additionally, the medial side of the cheek region of AMNH 5118 shows that the quadratojugal is anterodorsally expanded underneath the jugal to a similar degree and shape as TMP 1997.99.1. However, possible contributions to the actual cavum tympani of AMNH 5118 are not discernable based on the fossil.

### Squamosal

In TMP 1997.99.1, the squamosals are not preserved in articulation (Figs. 2A, B, 3C, D; Brinkman et al., 2006). They are floating in the surrounding matrix within the upper temporal fossa (Fig. 3C, D). This preservation prevents the identification of all contacts with certainty. Indeed, there is particular ambiguity regarding the possible contacts with the jugal, the parietal, and opisthotic, all of which are discussed below. Contacts with the quadrate, the quadratojugal, and postorbital, however, were certainly present.

The squamosal of TMP 1997.99.1 has an elongated, tapering anterior process (Fig. 2A, B), which extends along the posterior margin of the upper temporal emargination. The lateral surface of the anterior process hereby underlaps the postorbital, and, to a lesser extent, the quadratojugal. This strong overlapping articulation between postorbital and squamosal differs from the articulation seen in extant chelonioids, in which the squamosal is anteriorly broad (instead of tapering), and in which the contacts with the postorbital and quadratojugal are simple contacts that abut along their respective margins.

**Fig. 7:**
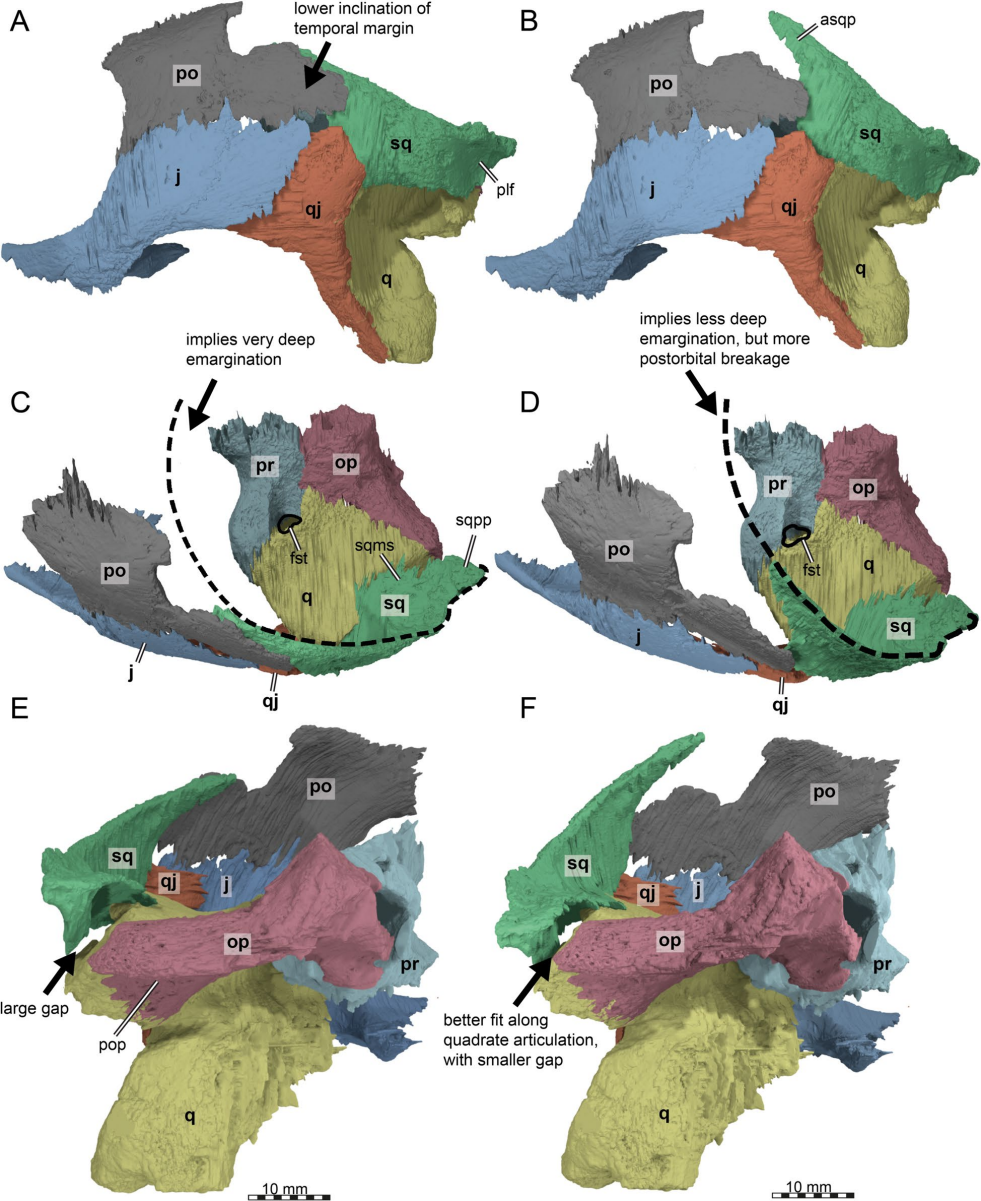
Three-dimensional renderings of the partial left lateral skull side of *Nichollsemys baieri* (TMP 1997.99.1) with the squamosal rotated into alternative positions. **A, C, E** squamosal with low inclination. **B, D, F** squamosal with high inclination. **A, B** left lateral view; **C, D** dorsal view; **E, F** posterior view. Note that bones are labelled in bold and features labelled in regular font. *aspq* anterior squamosal tapering process, *fst* foramen stapedio-temporale, *j* jugal, *op* opisthotic*, plf* posterolateral fossa, *po* postorbital, *pop* paraoccipital process prootic, *q* quadrate, *qj* quadratojugal, *sq* squamosal, *sqms* squamosal medial sheet, *sqpp* squamosal posterior process

We produced a partial skull reconstruction by digitally re-articulating the squamosal into its original position (Fig. 7). As there is uncertainty to reconstructions, we produced two alternatives, which primarily differ in the angle with which the squamosal caps the cavum tympani (Fig. 7). This leads to different interpretations regarding the upper (= posterodorsal) temporal emargination. In particular, when the anterior process is relatively strongly downturned (Fig. 7A, C, D), the emargination becomes quite deep (Fig. 7C). This implies a deeper emargination than in *Toxochelys latiremis* (Matzke, 2009), but still a less deep emargination than in extant or fossil members of the sister clade of chelonioids, Chelydroidea (e.g., *Emarginachelys cretacea*: Whetstone, 1978; *Leiochelys tokaryki*: Brinkman et al., 2022; *Protochelydra zangerli*: Erickson, 2010; extant chelydrids: Joyce, 2016; extant kinosternids: Joyce & Bourque, 2016). This reconstruction, however, also implies a slightly larger gap in the capping of the cavum tympani along the quadrate contact (Fig. 7E). An alternative orientation of the squamosal of TMP 1997.99.1 (Fig. 7B, D, F), which minimizes the aforementioned cavum tympani gap (Fig. 7F), results in a strongly dorsally oriented anterior squamosal process (Fig. 7B). This reconstruction implies that more bone of the postorbital (and parietal) is posteriorly missing in comparison to the other reconstruction. It also implies a less deep upper temporal emargination (Fig. 7D). This is closer to the condition of *Toxochelys latiremis* (Matzke, 2009). However, this also implies the upper temporal emargination margin to be strongly posteroventrally inclined (Fig. 7B), which is generally not observed in turtles, including *Toxochelys latiremis* (Matzke, 2009). Thus, we prefer the first-described reconstruction with a lower angle of the anterior process.

A number of morphological statements can be made about the squamosal of TMP 1997.99.1 regardless of exact orientation. The potential jugal–squamosal contact depends on the margins of the jugal, quadratojugal, and postorbital in this area and cannot be determined with certainty. A contact with the parietal is extremely unlikely, when a “regular” parietal morphology is assumed as the tapering tip of the squamosal of TMP 1997.99.1 seems fully preserved. However, there is one way in which a squamosal–parietal contact could have existed: *Toxochelys latiremis* has a highly unusual parietal morphology (Matzke, 2009) with a very long posteromedial process that reaches the squamosal. We can verify its presence based on examinations of the fossil and 3D models of AMNH FARB 5118. Given the many similarities between *Toxochelys latiremis* and TMP 1997.99.1, it is certainly possible that the same unusual process existed in the latter.

The ventral surface of posterior part of the squamosal of TMP 1997.99.1 certainly capped the cavum tympani formed by the quadrate (Figs. 4, 7A, B). However, although this surface is slightly concavely curved, it has no deeper cavity of excavation that would suggest the presence of an antrum postoticum distinct from the cavum tympani. This posterodorsal expansion of the cavum tympani is typically present in the squamosal of turtles (Gaffney, 1979), but reduced in chelonioids. A distinct antrum postoticum is already absent in *Toxochelys latiremis*, although the definition of this structure varies slightly between authors. For example, Matzke (2009) already accepts the capping of the quadrate by the squamosal as indicating the presence of a ‘true’ antrum postoticum in *Toxochelys latiremis*, even when the finger-like extension into the bone is absent.

The squamosal of TMP 1997.99.1 forms a relatively short, medially projecting, horizontally oriented sheet of bone that lies on the dorsal surface of the quadrate within the floor of the upper temporal fossa (Fig. 7C, D). This medial sheet, however, does not project over the entire quadrate surface to reach the opisthotic. We are unaware of any extant or fossil turtle that lacks this contact and the morphology of TMP 1997.99.1 specifically contrasts with the condition of extant chelonioids, in which there always is a broad squamosal–opisthotic contact within the temporal fossa. This morphology constitutes another similarity to *Toxochelys latiremis,* in which the squamosal facets on the quadrate (e.g., AMNH 1496) or the articulated squamosals (e.g., AMNH 5118) show that the contact with the opisthotic was a lot smaller than in extant chelonioids, and limited to the posterior tip of the paroccipital process, as we also propose was the case for TMP 1997.99.1. Lastly, the posterior end of the squamosal of TMP 1997.99.1 is again similar to *Toxochelys latiremis*, but differs from the morphology of extant chelonioids. In TMP 1997.99.1 and extant cheloniids, the dorsal margin of the upper temporal emargination forms a rim that curved posteromedially toward the paraoccipital process (Fig. 7E, F). This morphology defines a posterolateral surface of the squamosal. In TMP 1997.99.1 and *Toxochelys latiremis* (AMNH 5118), this surface bears a single, shallow fossa (Fig. 7A, B). In extant cheloniids, this surface is additionally crossed by at least one robust, vertically trending ridge, creating at least two fossae. These ridges and fossae are usually interpreted to be muscle attachment sites, and the simpler morphology of TMP 1997.99.1 and *Toxochelys latiremis* suggests differences in muscle attachment between these Cretaceous species and their extant relatives.

### Premaxilla

The premaxillae of TMP 1997.99.1 are fairly complete, but their labial ridge is slightly damaged, particularly that of the right one (Fig. 3A, B).

The premaxilla of TMP 1997.99.1 (Figs. 2, 3, 5) is a small, paired bone. It forms the most anterior part of the snout (Fig. 3A, B), the anterior part of the triturating surface (Fig. 2C, D), and the ventral surface of the fossa nasalis (Fig. 5). The distinct labial ridge, which is jointly formed with the maxilla, lacks even the hint of notches or processes (Figs. 2C, D, 3A, B), in contrast to *Dermochelys coriacea*, which exhibits tooth-like tomiodonts (Nick, 1912). The premaxilla of TMP 1997.99.1 meets the maxilla laterally, the vomer posteriorly, and its counterpart medially (Fig. 2). Posteriorly, the premaxilla forms the anterior rim of a thin and anteroposteriorly elongated foramen praepalatinum, the posterior margin being formed by the vomer (Fig. 2C, D). The dorsal opening of the foramen praepalatinum is associated with an anteriorly extending, dorsally open groove in the floor of the nasal cavity (Fig. 2A, B). This groove leads anteriorly into an additional, unnamed foramen at the base of the ascending part of the premaxilla that forms the bone wall ventral to the external naris (Fig. 2A, B). Paired foramina praepalatina are generally present in *Toxochelys latiremis* (Matzke, 2009), and these are dorsally also associated with a groove (e.g., AMNH 5118). Although foramina praepalatina are absent in protostegids (Evers et al., 2019a), extant cheloniids (e.g., *Lepidochelys olivacea*: SMNS 11070), and *Dermochelys coriacea* (Nick, 1912), the dorsal groove and anterior, unnamed foramina seen in TMP 1997.99.1 and *Toxochelys latiremis* (e.g., AMNH 1497, USNM 11560) are also present in these taxa with the exception of *Dermochelys coriacea*. On their dorsal side, the premaxillae of TMP 1997.99.1 jointly form a low median ridge that connects posteriorly with that of the vomer. At the anterior end of this ridge, a pocket or small cavity appears to be present that leads anteroventrally into the region between both premaxillae (Fig. 2A, B). This has also been described for *Rhinochelys pulchriceps* (Evers et al., 2019a), a protostegid, and can furthermore be observed in *Toxochelys latiremis* (AMNH 5118). Extant cheloniids also have a small gap in this area, but it is not as broad as in TMP 1997.99.1 or *Toxochelys latiremis*. The ventral side (Fig. 3C, D) of the interpremaxillary region of TMP 1997.99.1 is characterized by a broad, but shallow pit (Fig. 2C, D) that accommodates the dorsal labial structures of the dentary (see dentary for peculiarities in TMP 1997.99.1).

### Maxilla

The maxilla of TMP 1997.99.1 (Figs. 2, 3, 4, 5) is a large, paired bone that forms most of the upper jaw. It meets the premaxilla and vomer anteromedially (Fig. 2C, D), the prefrontal dorsally (Fig. 2A, B), and contacts the palatine medially between the foramen orbito-nasale and foramen palatinum posterius (Fig. 2C, D). The maxilla shows, in addition, a contact with the pterygoid and jugal at its posterior end (Fig. 2C, D), lateral to the posterior rim of the foramen palatinum posterius (Fig. 3C, D). The maxilla forms the anterior quarter of the orbital rim, and forms large parts of the floor of the orbital cavity between the palatine and the jugal (Fig. 2A, B). This part of the maxilla tapers posteriorly and contacts the pterygoid lateral to the foramen palatinum posterius. This contact, as well as the large size and oval shape of the foramen palatinum posterius are basically identical with the morphology in *Toxochelys latiremis* (AMNH 5118; partly contrasting Matzke, 2009). The maxilla of TMP 1997.99.1 forms parts of the lateral margin of the foramen orbito-nasale (Fig. 2A, B), which is otherwise formed by the prefrontal and palatine (Fig. 4). Anterior to the foramen orbito-nasale, the maxilla floors the nasal passage and the lateral third of the fossa nasalis. At its posterolateral end, the maxilla forms a posteriorly tapering process that underlaps the jugal and participates in the anterior portion of the cheek emargination (Fig. 4). The labial ridge of TMP 1997.99.1 is distinct and sharp (Fig. 2C, D), but lacks tooth-like processes or notches, as are present in *Dermochelys coriacea* (Nick, 1912). The profile of the labial ridge in lateral view is slightly downturned along the central parts of the maxilla (Fig. 4). This curvature continues onto the premaxillae. The external maxillary surface below the orbit shows the presence of numerous neurovascular foramina that probably indicate the former presence and extent of a keratinous rhamphotheca (Figs. 3, 4). These foramina connect internally with a central canal that traverses most of the maxilla anteroposteriorly, the canalis alveolaris superior. This canal is connected to a relatively large foramen alveolare superius, which is located within the lateral margin of the foramen orbito-temporale. Internally, the canalis alveolaris superior is additionally connected to a posteriorly directed canal, the canalis infraorbitalis. This canal is smaller in diameter than the canalis alveolaris superior, and also has a smaller opening within the dorsal surface of the maxilla. This foramen, the foramen supramaxillare, can only be seen in the CT scans with certainty on the right side and opens centrally on the dorsal surface of the maxilla that forms the floor of the orbital fossa (Fig. 2A, B). The maxilla forms most of the triturating surface. A low, but distinct lingual ridge is formed jointly with the palatine that runs parallel to the labial ridge (Fig. 2C, D).

### Vomer

The vomer of TMP 1997.99.1 (Figs. 2, 3, 5) is an unpaired element medially situated in the palatal area. The vomer meets the premaxillae anteriorly, the maxillae anterolaterally, the palatines laterally, the prefrontal anterodorsally, and the pterygoids posteriorly (Figs. 2C, D, 5). The latter contact is absent in protostegids, but present in the stem chelonioid *Toxochelys latiremis* (Matzke, 2009) and all extant chelonioids. For the purpose of this description, we distinguish three parts of the vomer of TMP 1997.99.1: a mediolaterally broad part anterior to the internal naris, a central part that supports the dorsal vomerine structures, and a posterior part. The vomer is mediolaterally broadest anterior to the internal nares, where it has two ventrally projecting lateral processes that contact the premaxillae anteriorly and the maxillae anterolaterally (Fig. 2C, D). The central area between these processes on the ventral surface of the vomer is mediolaterally concave. This concavity is posteriorly continuous with the deep fossa surrounding the central part of the vomer, in which the internal nares are located. This part of the vomer is very similar to *Toxochelys latiremis* (Matzke, 2009) and other taxa that lack secondary palates, but differs strongly form modern cheloniids and other chelonioids with secondary palates, in which the anterior part of the vomer generally articulates to the same bones, but in which it is developed as a horizontal plate that is integrated into the triturating surfaces of the maxilla (Brinkman et al., 2006). The anterior part of the vomer of TMP 1997.99.1 forms the posterior rim of the foramina praepalatina (Fig. 2C, D). More posteriorly, the ventrolateral processes form the medial wall and floor of the nasal passages. The vomer does not participate in the medial rim of the foramen orbito-nasale, which occurs in several chelonioids (e.g., *Lepidochelys olivacea*: SMNS 11070) but is not universally present within the group. On the dorsal surface that forms parts of the floor of the nasal cavity, the vomer of TMP 1997.99.1 forms a low median ridge that continues anteriorly onto the premaxillae.

The central part of the vomer is dorsally slightly arched with respect to the anterior part and the posterior process (Fig. 5). The dorsal surface of the central part is covered by two anterodorsally directed columnar processes that articulate with the prefrontal to form the medial wall and dorsal roof to the nasal passage (Figs. 3A, B, 5). These dorsal processes are medially separated by a narrow and deep median groove called the sulcus vomeri, which represents the ventral third of the keyhole-shaped fissura ethmoidalis (Fig. 3A, B). This mimics the morphology of most turtles, with the exception of *Dermochelys coriacea* among cryptodires, in which the dorsal processes are greatly reduced, instead forming two low ridges on either side of the sulcus vomeri. In moderns cheloniids and in *Allopleuron hofmanni*, the processes are similar in height but due to the formation of a secondary palate, the nasal passages are considerably larger than in TMP 1997.99.1, making the vomer dorsoventrally higher.

The posterior part of the vomer forms an anteroposteriorly elongated and slightly posteriorly descending posterior process that lies between the palatines and posteriorly articulates with the pterygoids (Fig. 2C, D; Brinkman et al., 2006). The dorsal surface of this part of the vomer shows a sharp median crest that is posteriorly continued by an interpterygoid ridge and that likely anchored the interorbital septum. There is no posterior extension of the sulcus vomeri as a dorsally open trough. The ventral surface of the vomer is flat and a ventral keel is absent (Fig. 2C, D). These conditions strongly differ from those found in the protostegids, which possess a posteriorly less elongated vomer with a slight keel (Evers et al., 2019a)*.* In modern chelonioids a keel is variously present (e.g., *Chelonia mydas*, *Dermochelys coriacea*; but see *Lepidochelys olivacea* for the absence of a keel), but usually restricted to the part of the vomer that connects the central column between the nasal passages with the posterior vomer process.

### Palatine

The palatine of TMP 1997.99.1 is a paired bone forming the lateral parts of the palate adjacent to the vomer (Figs. 2, 3, 4, 5). The palatine contacts the maxilla anterolaterally, the vomer medially, the pterygoid posteriorly (Fig. 2C, D), and the descending process of the prefrontal anterodorsally (Fig. 4). A lateral contact with the jugal and a median interpalatine contact are clearly absent (Fig. 2). The palatine consists of a horizontally flat sheet forming large parts of the floor of the orbital cavity and an anteroventrally projecting process that contributes to the triturating surface (Fig. 2C, D). The horizontal sheet of the palatine shows anteriorly a dorsally elongated contact with the descending process of the prefrontal and forms the dorsal roof of the meatus choanae. It also forms the medial rim of the foramen orbito-nasale (Figs. 2A, B, 4). The medial border of the horizontal sheet of the palatine strongly overlaps the vomer, reducing the dorsal exposure of the latter.

Our 3D models of TMP 1997.99.1 show slight irregularities along the palatine–pterygoid suture (Fig. 2C, D). This is because both bones are tightly interfingered and the individual struts and pockets of this articulation are difficult to separate with great precision due to the high bone porosity and fine suture lines. Nevertheless, the position and orientation of the suture is clear and can furthermore be verified in photographs (Fig. 1B). The palatine and vomer jointly meet the pterygoid in a roughly straight, transverse suture at the posterior level of the foramen palatinum posterius (Fig. 2C, D). The medial margin of the relatively large, anteroposteriorly oval foramen palatinum posterius is formed by the palatine (Fig. 2C, D; Brinkman et al., 2006), whereas the posterior border is largely formed by the pterygoid, which contacts the maxilla in this area as the bone that forms the lateral margin of the foramen. This is comparable to *Toxochelys latiremis*, in which well-preserved specimens also show the same condition (e.g., AMNH 5118; Matzke, 2009). In *Toxochelys latiremis*, there may be some variation to this morphology, as some specimens (e.g., USNM 11560; Matzke, 2009) show that the palatine have a short, laterally recurved process along the posterior border of the foramen palatinum posterius, which minimizes the pterygoid’s contribution to its margin, but which generally does not seem to reach the maxilla. The foramen palatinum posterius is completely absent in extant chelonioids and laterally open in protostegids.

The base of the ventrolateral process of the palatine of TMP 1997.99.1 is constricted between the foramen orbito-nasale and foramen palatinum posterius but broadens laterally along its contact with the maxilla in both anterior and posterior directions (Fig. 2C, D). Along this contact, it reaches anteriorly to contact the posterior tip of the anteroventral process of the vomer in the lateral rim of the internal naris (Fig. 2C, D). This vomer–palatine contact anterior to the internal naris is typical of chelonioids and also present in *Toxochelys latiremis* (Matzke, 2009), but largely absent among other cryptodires. The ventral surface of the anterolateral process of the palatine of TMP 1997.99.1 contributes narrowly, but distinctly, to the triturating surface (Fig. 2C, D). A similar contribution is also present in *Toxochelys latiremis* (Matzke, 2009) and modern chelonioids, but it is absent in some protostegids, such as *Desmatochelys lowii* (Raselli, 2018)*.* Together with the maxilla, the palatine of TMP 1997.99.1 forms a low and blunt lingual ridge that medially frames the triturating surfaces (Fig. 2C, D).

### Quadrate

The quadrate of TMP 1997.99.1 (Figs. 2, 3, 4, 6, 7) is a paired bone situated in the posterolateral skull region. It forms the cavum tympani, the incisura columellae auris, and the processus articularis (Figs. 4, 7). The ventral end of the processus articularis is finished by the facet for the mandibular articulation (Fig. 2C, D). The quadrate meets the prootic anteromedially (Figs. 2, 6, 7), the opisthotic posteromedially (Figs. 2, 7C, D), the pterygoid ventromedially (Figs. 2, 3C, D, 6), and the quadratojugal anteriorly (Fig. 4). Even if both squamosals are disarticulated, it is apparent that the quadrates contacted them posterodorsally, but also that the contact was not very tight (Fig. 7). Instead, the quadrate surface that faces the squamosal is basically flat and any type of interdigitation of these bones must have been absent. This is also observed in crown chelonioids, but not apparent in non-chelonioid outgroups. A processus epipterygoideus of the quadrate is absent (Fig. 6).

The quadrate forms the lateral half of the distinct, anteriorly directed processus trochlearis oticum (Fig. 6A). The process is well rounded and occupies a relatively small part of the mediolateral width of the otic chamber, so that it is well separated laterally from the internal surface of the cheek and medially well separated from the braincase. The processus trochlearis oticum of TMP 1997.99.1 is similar of that of *Toxochelys latiremis* (see in Matzke, 2009: Figs. 4, 5), although somewhat more prominent. This process is weaker and projects less prominently into the temporal fossa in *Allopleuron hofmanni* (see Mulder, 2003: pls. 11 and 12) and is absent in *Dermochelys coriacea* (see Nick, 1912: pls. 1 and 2), whereas the development of the process is variable among extant cheloniids.

The quadrate of TMP 1997.99.1 forms the lateral rim of the foramen stapedio-temporale (Figs. 2A, B, 7C, D), which is a very large opening as is typical for chelonioids (Rollot et al., 2021). This foramen represents the dorsal aperture of the canalis stapedio-temporale, which is otherwise formed by the prootic and traverses the otic capsule dorsoventrally and into the anterior region of the cavum acustico-jugulare. The quadrate also forms the lateral wall of the cavum acustico-jugulare and the lateral rim of its posterior opening, the fenestra postotica (Fig. 3C, D). Within the cavum acustico-jugulare, the quadrate minorly contributes to the posterolateral wall of the canalis cavernosus, which is otherwise formed by the prootic and pterygoid.

The cavum acustico-jugulare is connected to the cavum tympani via the incisurae columellae auris, which appears as a posteroventral notch in the cavum tympani (Fig. 3C, D) that funneled the stapes through the middle ear region toward the fenestra ovalis of the inner ear and which partially includes the Eustachian tube*.* The surface of the funnel-shaped cavum tympani of TMP 1997.99.1 is formed by the quadrate, but its anterior margin is formed by the quadratojugal and its dorsal margin by the squamosal (Figs. 4, 7). In the posterodorsal part of the cavum tympani, the quadrate is anterolaterally recurved to form a hook-like process that surrounds the posterodorsally open aperture of the antrum postoticum (Fig. 4A, B). This part of the quadrate was topped by the posterior part of the squamosal (Fig. 7), without the formation of a distinct antrum postoticum (see squamosal). Despite the deformation that affects specimens of *Toxochelys latiremis*, their quadrate shows a similar overall shape to that of TMP 1997.99.1, particularly in that the quadratojugal participates to the anterior rim of the cavum tympani (Matzke, 2009). In protostegids, the antrum postoticum condition is variable, and can be widely open in *Protostega gigas* to absent in some of the *Rhinochelys pulchriceps* specimens, showing intraspecific variation in the latter (Evers et al., 2019a). In *Dermochelys coriacea* (see pls. 1 and 2 in Nick, 1912), as in modern cheloniids, the antrum postoticum is absent or strongly reduced (see squamosal).

The processus articularis of TMP 1997.99.1 is located ventral to the incisura columellae auris (Fig. 3C, D). Its posterior surface is smooth and lacks an infolded ridge of the quadrate (Figs. 3C, D, 7E, F). The foramen chorda tympani quadrati is not visible in the posterior surface of the processus articularis. The processus articularis projects strongly ventrally below the level of the palate (Figs. 3C, D, 4), as in all chelonioids, and is situated at a level that is anterior to the foramen magnum (Fig. 2C, D). The condylus mandibularis of TMP 1997.99.1 shows a shallow and wide anteroposterior groove that separates the articular surface into lateral and medial subfacets (Fig. 2C, D). On the left side of the specimen, the pterygoid marginally participates in the medial facet of the condylus mandibularis (Figs. 2C, D, 5D, 6A). This contact is not preserved on the right side of the specimen, but here the process still extends far down the quadrate’s articular process, similar to the condition in the early protostegid *Rhinochelys pulchriceps* (Evers et al., 2019a). Generally, this does not seem to occur in other Cretaceous pan-chelonioids, including *Toxochelys latiremis* (Matzke, 2009), in which the pterygoid ends before reaching the ventral end of the processus articularis of the quadrate.

### Pterygoid

Both pterygoids of TMP 1997.99.1 (Figs. 2, 3, 4, 5, 6) are complete and well-preserved, allowing the identification of many internal canals and structures. The pterygoid is a large, complex, paired bone that connects the palate to the basicranium. This bone can be divided into an anterior part that is in connection with the palate and that forms the external (transverse) pterygoid process, and a posterior process that floors the basicranium. The pterygoid contacts the exoccipital, basioccipital, parabasisphenoid, prootic, quadrate, vomer, palatine, jugal, maxilla, as well as the other pterygoid (Figs. 2, 3, 4, 5, 6).

The anterior part of the pterygoid is strongly expanded medially and separated from the posterior process by a constriction in the center of the bone that forms a deep lateral notch facing the subtemporal fenestra (Fig. 2C, D). The lateral expansion of the pterygoid forms a well-defined external pterygoid process, which contributes to the posterior rim of the foramen palatinum posterius and contacts the maxilla and the jugal anterolaterally (Fig. 2C, D; Brinkman et al., 2006). The process expands deeply posterolaterally into the subtemporal fossa. The anterior morphology of the pterygoid is nearly identical to that of *Toxochelys latiremis* (Matzke, 2009), but important differences to other pan-chelonioids exist. Specifically, the transverse pterygoid process is strongly reduced to absent in cheloniids and does not occur in *Dermochelys coriacea* (Gaffney, 1979), whereas it does not contact the maxilla anterolaterally in protostegids for which this region is well known, so that it becomes a “free” process that projects laterally into the subtemporal fenestra (e.g., Evers et al., 2019a; Hirayama, 1998; Kear & Lee, 2006; Raselli, 2018). The lateral margin of the external pterygoid process of TMP 1997.99.1 is capped by a well-developed, obliquely oriented, vertical flange that is ovoid in outline (Figs. 4, 6). This condition is similar to *Toxochelys latiremis* (Matzke, 2009). Anteriorly, the pterygoid of TMP 1997.99.1 contacts the palatine and, more medially, the vomer along a broad suture (Fig. 2C, D; Brinkman et al., 2006).

The pterygoids of TMP 1997.99.1 are in contact with each other along a median suture that extends along the anterior half of the bone and that exceeds the length of the anteroposterior exposure of the parabasisphenoid (Figs. 2C, D, 5; Brinkman et al., 2006). The ventral interpterygoid suture does not form a very deep ridge, but is nevertheless slightly raised and is V-shaped in coronal cross section of the CT slices (Fig. 2C, D). Brinkman et al. (2006) considered this ridge to be homologous with that seen in more derived chelonioids, and the morphology indeed differs from that of *Toxochelys* spp., in which a midline ridge of any kind is absent. For example, *Toxochelys mooreviliensis* (FMNH PR 219) with its near perfectly preserved basicranium has a completely flat interpterygoid surface. However, we here note that sea turtles in which the presence of a median ridge is unambiguous, have ridges that are much deeper, forming often a lamina-like crest, again therefore displaying a different morphology. This can for example be exemplified by *Allopleuron hofmanni* (NHMUK R4213) and *Argillochelys antiqua* (NHMUK R41636). Although the weak ridge in TMP 1997.99.1 is somewhat similar to the derived morphology of the aforementioned presumed fossil cheloniids in the sense that a ridge is apparent, we score the respective character (ch. 104) as state “0”, as *Nichollsemys baieri* fulfills the “incipient” presence of the ridge that is detailed in the character state definition. In future versions of the matrix, it may be good to revise this character into an ordered multistate character, in which the incipient presence of the ridge is not coded with its absence, but as an intermediate state. However, as we do not revise other characters of our baseline matrix, we only suggest this change here for the future. In the posterior part of the pterygoid of TMP 1997.99.1, the bone forms a posterior process that diverges posterolaterally from the midline to accommodate the triangular ventral exposure of the parabasisphenoid and basioccipital (Fig. 2C, D). To the right and left, these two bones are bordered by low, but sharp crests (Fig. 2C, D) that are formed by the pterygoid and have sometimes been called ventral pterygoid ridges (Evers et al., 2019a). These ridges terminate near the posterior end of the pterygoids. The ventral pterygoid ridges of *Nichollsemys baieri* show a reverse “V” pattern (Brinkman et al., 2006), which is observed in many chelonioids (Gaffney, 1979). The ventral surface of the pterygoid in TMP 1997.99.1 shows also a pterygoid fossa that is moderately deep and laterally bordered by a distinct and rounded ventrolateral ridge formed by the ramus of the pterygoid that extends to the mandibular articulation process of the quadrate (Fig. 2C, D). The pterygoid has a deep extension along this process of the quadrate and closely approximates the articulation surface (Figs. 2C, D, 5). This is better preserved on the left side of the skull, where the pterygoid even seems to make a small contribution to the articulation surface itself. On the right side, this cannot be verified, as the most of the ventral extend of the pterygoid seems to be broken.

**Fig. 8:**
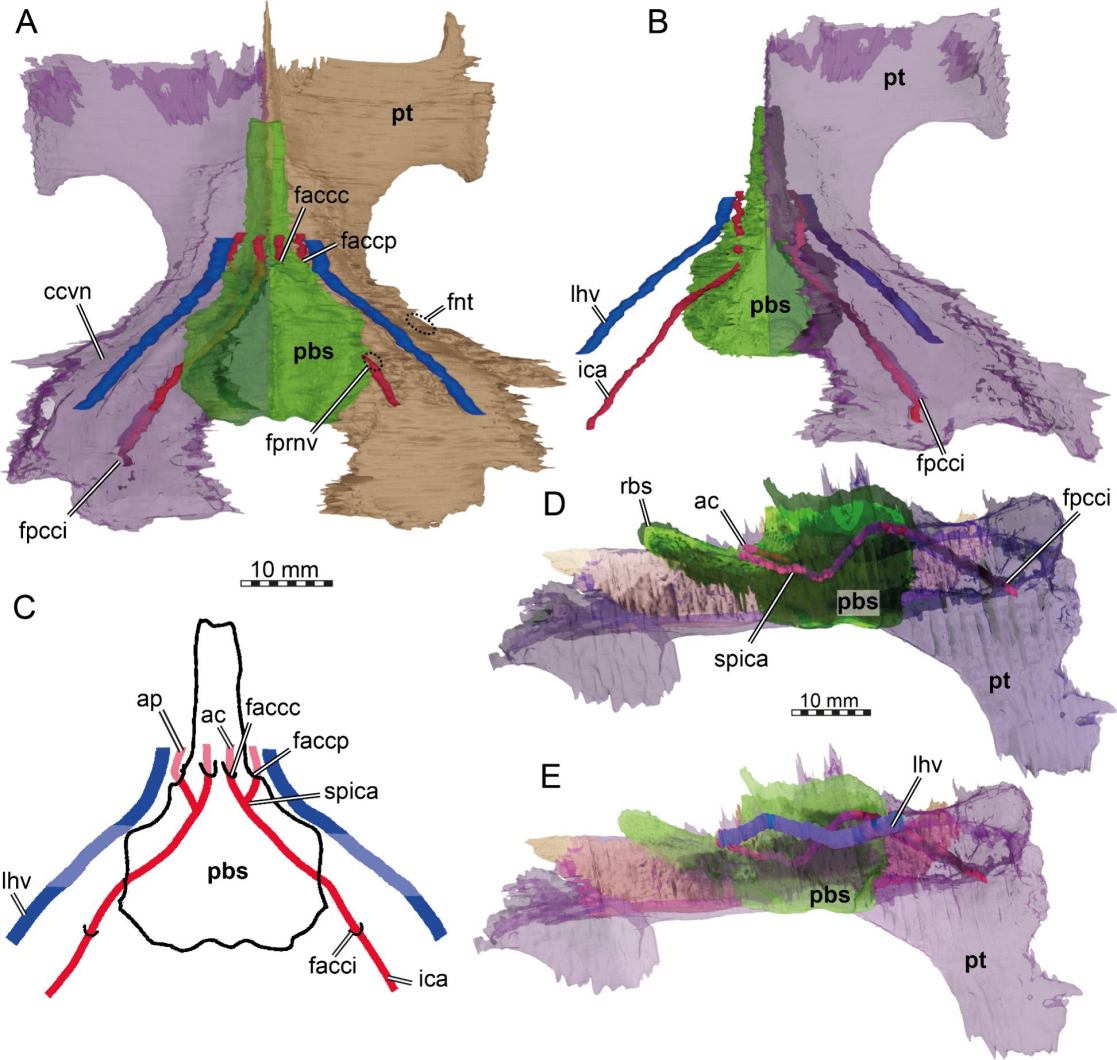
Three-dimensional reconstruction of the partial basicranium of the holotype specimen of *Nichollsemys baieri* (TMP 1997.99.1) with inferred cranial circulation based on canal endocasts. **A** dorsal view, with left side rendered transparent; **B** ventral view, with left side rendered transparent; **C** interpretative line-drawing of the dorsal view; **D** lateral left view without lateral head vein; **E** lateral left view including lateral head vein. Note that bones are labelled in bold and features labelled in regular font. *ac* cerebral artery, *ap* palatine artery, *faccc* foramen anterius canalis caroticus cerebralis, *faccp* foramen anterius canalis carotici palatinum, *fnt* foramen nervi trigemini, *fprnv* foramen pro ramo nervi vidiani, *ica* internal carotid artery, *lhv* lateral head vein, *pbs* parabasisphenoid, *pt* pterygoid, *rbs* rostrum basisphenoidale, *spica* split of the internal carotid artery

**Fig. 9:**
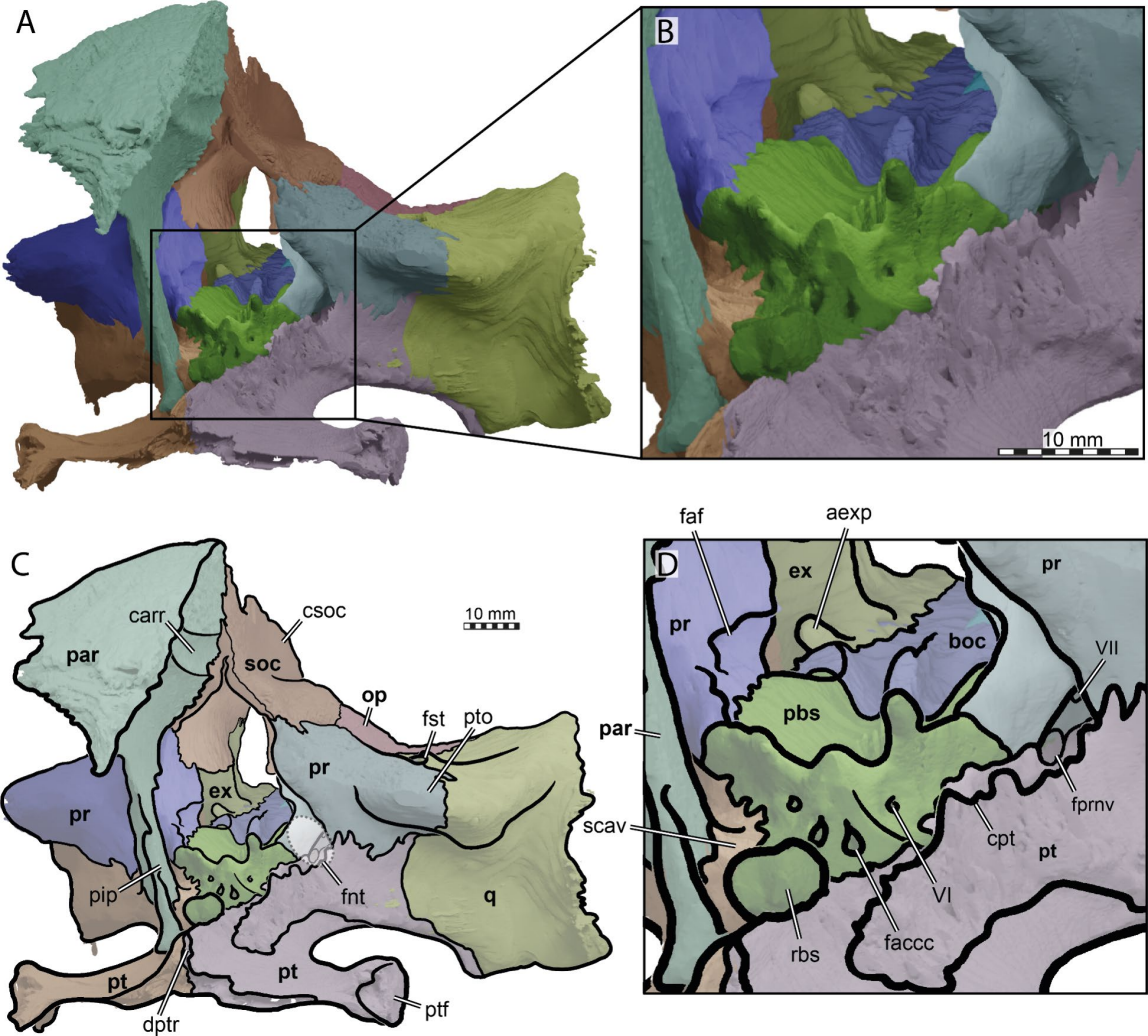
Three-dimensional renderings of the partial braincase of the holotype specimen of *Nichollsemys baieri* (TMP 1997.99.1) in anterior and slight lateral view. **A** overview of braincase morphology; **B** close-up onto the parabasisphenoid and anterior canalis cavernosus area; **C** interpretative line-drawing based on A; **D** interpretative line-drawing based on B. Note that bones are labelled in bold and features labelled in regular font. *aexp* anterior (hooked) exoccipital process, *boc* basioccipital, *carr* cartilaginous rider, *cpt* crista pterygoidei, *csc* crista supraoccipitalis, *dptr* dorsal interpterygoid ridge, *faccc* foramen anterius canalis caroticus cerebralis, *faf* fossa acustico-facialis, *fnt* foramen nervi trigemini, *fprnv* foramen pro ramo nervi vidiani, *fst* foramen stapedio-temporale, *par* parietal, *pbs* parabasisphenoid, *pip* processus inferior parietalis, *pr* prootic, *pt* pterygoid, *ptf* pterygoid flange, *pto* processus trochlearis oticum, *q* quadrate, *rbs* rostrum basisphenoidale, *scav* sulcus cavernosus, *soc* supraoccipital, *VI* foramen nervi abducentis, *VII* foramen nervi facialis

The posterior pterygoid process of TMP 1997.99.1 has a broad exposure on the posterior surface of the skull (Fig. 3C, D), where it forms the ventral margin of the fenestra postotica. Besides contacting the basioccipital, the posterior process of the pterygoid also has a very broad contact with the exoccipital medial to the fenestra postotica (Fig. 3C, D). This broad exposure is unusual, especially as it partly separates the basioccipital and exoccipital lateral to the occipital condyle (Fig. 3C, D). The broad posterior exposure of the pterygoid forms a plateau-like, flat area ventromedial to the openings for the hypoglossal nerve, which are close to the exoccipital-pterygoid suture (Fig. 3C, D). In extant chelonioids (i.e., cheloniids and *Dermochelys coriacea*), the basioccipital and exoccipital are closely occluded in this area and the pterygoid is far laterally removed from the hypoglossal foramina. As in all pan-chelonioids, the fenestra postotica of TMP 1997.99.1 is coalescent with the foramen jugulare posterius (Fig. 3C, D). The foramen posterius canalis carotici interni opens within the posterior process of the pterygoid (Brinkman et al., 2006), in a position between the posterior end of the ventral ridge that parallels the contact with the basioccipital and the margin of the fenestra postotica (Figs. 2C, D, 8). The foramen is relatively small compared to the foramen stapedio-temporale, which is approximately four times the diameter of the foramen posterius canalis carotici interni. The internal carotid canal then traverses anterodorsally through the pterygoid (Fig. 8). Slightly anterior to the level of the labyrinth, the internal carotid artery pierces through the dorsal pterygoid surface where it is overlain by the prootic (Fig. 8A) and the canal then continues anteriorly along the pterygoid-prootic suture. As the internal carotid canal approaches the parabasisphenoid suture, it forms a large internal cavity between the pterygoid, prootic, and parabasisphenoid (Fig. 9). From this cavity, there are two openings. Medially, the internal carotid artery canal enters the sutural region of the parabasisphenoid and pterygoid and continues farther anteriorly (Fig. 8), where it separates into a medial cerebral artery canal within the parabasisphenoid and an anteriorly continuing palatine artery canal. Laterally, the cavity opens into the canalis cavernosus via an unusually large foramen pro ramo nervi vidiani (the palatine artery foramen of Brinkman et al. (2006)), which is further described below (Figs. 8A, 9).

The dorsal surface of the posterior process of the pterygoid forms the majority of the floor of the cavum acustico-jugulare (Fig. 3C, D), including the recessus scalae tympani. There is no contact with the ventral tip of the processus interfenestralis of the opisthotic, so that a hiatus postlagenum remains. Thus, the pterygoid also floors the posterior parts of the cavum labyrinthicum, whereas anteriorly, the pterygoid surface is overlain by the ventral process of the prootic. At the anterior end of the cavum acustico-jugulare, the pterygoid and prootic form the posterior opening into the canalis cavernosus. The pterygoid forms the floor and lateral and medial margins of this canal along raised ridges. The medial of these ridges is unnamed and its medial side is bordered by the canalis caroticus interni. Both ridges are interrupted along their course by foramina (Fig. 9). The medial ridge bears the aforementioned, unusual, large foramen, which is much larger than the foramen posterius canalis carotici interni, but smaller than the foramen stapedio-temporale and which opens between the internal carotid canal and the canalis cavernosus (Fig. 9; Brinkman et al., 2006). The foramen is located in the prootic-pterygoid suture and is positioned just anteroventrally to the lateral foramen of the facial nerve canal within the ventral process of the prootic (see prootic) and thus just posterior to the level of the trigeminal foramen and foramen cavernosus (Fig. 9). In this area, one would usually expect the foramen (and canalis) pro ramo nervi vidiani, which carries the vidian nerve from the canalis cavernosus into the canal for the internal carotid artery (Gaffney, 1979; Rollot et al., 2021). As no separate foramen pro ramo nervi vidiani is evident in TMP 1997.99.1, we propose that the vidian nerve passed through the large foramen, essentially making it an enlarged foramen pro ramo nervi vidiani. A similarly enlarged foramen pro ramo nervi vidiani is absent in modern cheloniids (Rollot et al., 2021), and in the protostegid *Rhinochelys pulchriceps* (Evers et al., 2019a). However, a large foramen between the canalis cavernosus and carotid arterial system is also present in *Allopleuron hofmanni* (NHMUK R4213) as evident from CT-scan derived 3D models that we have of this specimen (Evers, 2021)*,* although this morphology, indeed this area, has not previously been described for *Allopleuron hofmanni* (Mulder, 2003). As the foramen of TMP 1997.99.1 is extraordinarily large for a nerve foramen, the possibility that other structures also passed through the foramen (e.g., the mandibular artery or an unnamed artery) cannot be excluded and is potentially supported by strange, burrow-like structures that extend through parts of the fossil and that may represent partial “soft tissue” preservation (see below). Brinkman et al. (2006) identified the large foramen as the passage for the palatine artery. However, given the identification of an alternative palatine artery canal (see below), and given that the mandibular artery of extant cheloniids branches off the palatine artery in a position anterior to the processus inferior parietalis (personal observation on diceCTed specimen of *Chelonia mydas*, UF-herp-51413), we herein favor an interpretation in which the large foramen only serves as the passage for the vidian nerve, while we cannot provide a satisfactory explanation of the large size of the foramen pro ramo nervi vidiani. Possibly, the close spacing of the canalis cavernosus and internal carotid artery canal led to a broad coalescence of these canals in the form of a ‘window’, similar to the coalescence of some other openings such as the foramen jugulare posterius and fenestra postotica in chelonioids. In TMP 1997.99.1, the vidian nerve enters the internal carotid canal by the foramen pro ramo nervi vidiani and, likely, follows the path of the carotid artery, before entering the canal for the palatine artery. The absence of a separate exiting foramen for the vidian nerve suggests the absence of a canalis nervus vidianus, as in all other chelonioids (Rollot et al., 2021).

The pterygoid ridge to the lateral side of the canalis cavernosus of TMP 1997.99.1 is deeply notched dorsally to form the ventral margin of the trigeminal foramen (Figs. 6, 9). This foramen is dorsoventrally elongated in TMP 1997.99.1 (Fig. 6) and ventrally extends nearly to the floor of the sulcus cavernosus. This posteroventral expansion of the foramen has been interpreted to be an osteological correlate for the mandibular artery leaving the trigeminal foramen (Evers & Benson, 2019). Anterior to the trigeminal foramen, the crista pterygoidea is formed as an extremely low, nearly absent ridge (Figs. 6, 9). Instead, the ventral process of the parietal is very deep and is weakly attached to the dorsal pterygoid surface (Figs. 5, 6, 9). There is no trace of an epipterygoid. The sulcus cavernosus extends anteroposteriorly between the medial surface of the ventral process of the parietal and the rostrum basisphenoidale of the parabasisphenoid (Fig. 9). The sulcus becomes anteriorly strongly constricted, such that the parietal nearly has a contact with the rostrum basisphenoidale, limiting the pterygoid exposure to a thin band between the two (Fig. 9).

The exiting foramen of the palatine branch of the carotid artery (foramen anterius canalis caroticus palatinum of Rabi et al., 2013; foramen anterius canalis carotici lateralis of Rollot et al., 2021) opens anteriorly into the floor of the sulcus cavernosus (Figs. 6, 8), in a small foramen between the pterygoid and the parabasisphenoid. This foramen was unnoticed in the original description (Brinkman et al., 2006). The position of the foramen is approximately at the same level as the exiting foramen for the cerebral branch of the carotid artery (foramina anterius canalis caroticus cerebralis sensu Rabi et al., 2013; foramina anterius canalis caroticus basisphenoidalis sensu Rollot et al., 2021) (Fig. 8). This condition differs from that of *Toxochelys mooreviliensis* (FMNH PR 219 and 27338), in which the foramen anterius canalis caroticus palatinum is situated at the midlength of the foramen anterius canalis carotici cerebralis and the anterior limit of the crista pterygoidei, so that is situated farther anteriorly than in TMP 1997.99.1. All *Toxochelys* spp. specimens for which we could evaluate this feature show several supernumerary foramina in the area of the foramen anterius canalis carotici palatinum, but CT scans of FMNH PR 219 show that these smaller foramina are not connected to the internal carotid arterial system. Despite some differences, the internal carotid artery canal branching system of TMP 1997.99.1 is a lot more similar to *Toxochelys latiremis* and *Toxochelys mooreviliensis* (not known for *Toxochelys browni*, which has been suggested to represent a different species) than originally described (Brinkman et al., 2006).

The anterior end of the rostrum basisphenoidale of TMP 1997.99.1 rests on the suture of the right and left pterygoids, which forms a raised dorsal median ridge (Figs. 5, 6, 9). The morphology of *Toxochelys* spp. is again slightly different in that the rostrum is not formed as a rod-like process and in that the interpterygoid suture forms more of an elevated platform than an elevated median ridge (e.g., Brinkman et al., 2006; Matzke, 2009). The dorsal surface of the pterygoid of TMP 1997.99.1 lateral to the contact with the parietals show no anterior exiting foramina for the vidian nerve, suggesting that these canals could not be identified in the CT scan or that the nerve course is fully associated with that of the palatine artery.

### Epipterygoid

An epipterygoid is absent in TMP 1997.99.1. There are several indications that this absence is genuine, as opposed to the epipterygoid being fused or falsely not identified by us. The first indication is that the CT scan of TMP 1997.99.1 generally shows all sutures relatively clearly, but an epipterygoid suture is undetectable in the area where the bone is expected to be if it were present. This could, in theory, be explained by fusion of the epipterygoid with the surrounding bones. However, the epipterygoid of presumably closely related taxa, particularly *Toxochelys mooreviliensis*, is banana-shaped, and lies on the surface of the pterygoid at the base of the secondary lateral braincase wall (e.g., FMNH PR 219). In *Toxochelys mooreviliensis*, the central aspect of the crescentic element overlaps the processus inferior parietalis with its dorsal margin (FMNH PR 219). Thus, assuming a similar epipterygoid shape for TMP 1997.99.1 the descending process of the parietal would either be expected to have anteroventrolateral and posteroventrolateral processes that expand onto the pterygoid surface or the pterygoid would be expected to form a dorsally rounded overlap onto the lateral surface of the descending process of the parietal. Neither of these morphologies are observed. TMP 1997.99.1 also lacks an epipterygoid process of the quadrate or clearly developed fossa cartilaginis epipterygoidei. Thus, we interpret the evidence as indicating the true absence of the epipterygoid. The absence of the epipterygoid is insofar interesting, as many fossil chelonioids do have epipterygoids, including Cretaceous species (e.g., *Toxochelys latiremis*, *Ctenochelys* spp., *Allopleuron hoffmanni*) and much younger Eocene stem cheloniids (e.g., *Argillochelys cuneiceps*, *Eochelone brabantica*), indicating that the epipterygoid may have been lost in TMP 1997.99.1 independently to the phylogenetically shallower loss in cheloniids.

### Supraoccipital

The ventral part of the supraoccipital that is integrated into the braincase is complete in TMP 1997.99.1, but the dorsal and posterior regions of the crista supraoccipitalis are broken (Figs. 2, 3, 4, 5, 9). The supraoccipital is an unpaired bone that dorsally roofs the posterior braincase. This bone is prolonged dorsally and posteriorly by a prominent sagittal crest called the crista supraoccipitalis. The length of this structure in TMP 1997.99.1 is unclear due to the aforementioned breakage. Nevertheless, it seems evident that the crest must have been quite long posteriorly, as the base of the crest at the break line is quite massive and as the preserved anterior portion of the crest already overlaps the full occipital condyle and expands beyond the level of the posterior squamosal tips (Fig. 2C, D). Thus, the crest was probably similar to that of *Toxochelys latiremis* (Matzke, 2009). Anteriorly, the supraoccipital contacts the parietals along a strait vertical suture that expands to the top of the crista supraoccipitalis (Fig. 5), which is completely overlain by the parietals and thus seem to lack any expression in the dorsal skull roof (Fig. 2A, B). The ventral part of the supraoccipital forms two ventrally directed lateral processes that roof the braincase in the shape of a narrow, reversed U (Fig. 3C, D, 5, 9). On both sides, the ventral rim of the lateral processes contacts the prootics anteriorly and the opisthotics more posteriorly (Figs. 2A, B, 6, 9). In cross section, the braincase has the shape of a reversed keyhole at the level of the supraoccipital–opisthotic contact, the dorsal constriction being formed by the supraoccipital. At their facet for the contact with the prootic and the opisthotic, these ventral processes show a small recess that house the dorsal ends of anterior and posterior semicircular canals of the labyrinth. At their posterior ventral ends the lateral processes contact the exoccipitals (Fig. 3C, D). The supraoccipital participates to the foramen magnum, and its posteroventral border is forming the dorsal rim of this aperture (Fig. 2C, D).

### Exoccipital

Both exoccipitals of TMP 1997.99.1 are complete (Figs. 3, 5). The exoccipital is a paired bone that forms the lateral rim of the foramen magnum (Fig. 3C, D). The exoccipital meets the supraoccipital dorsally, the opisthotic dorsolaterally, the posterior process of the pterygoid ventrolaterally, and the basioccipital medioventrally (Fig. 2C, D). A contact with the parabasisphenoid is clearly absent (Fig. 5). A median point contact with its counterpart is apparent above the condylus occipitalis.

The anterior surface of the exoccipital posteriorly encloses the recessus scalae tympani. The exoccipital contributes to both the lateral opening of the recessus scalae tympani, the fenestra postotica, the medial opening toward the cavum cranii, and the foramen jugulare anterius (Fig. 5). The ventral exoccipital process that abuts the para basisphenoid is anteriorly expanded in this region to form the ventral margin of the foramen jugulare anterius. This anterior process of the exoccipital just about contacts the processus interfenestralis of the opisthotic. The exoccipital forms a lateral expansion that contributes to the medial rim of the fenestra postotica (Fig. 3C, D). The exoccipital is further laterally expanded along the dorsal margin of the fenestra postotica and extends along the paroccipital process that is formed by the opisthotic (Fig. 3C, D).

The exoccipital forms a dorsal process that extends along the posterior surface of the opisthotic and forms the lateral wall of the foramen magnum. Dorsally, it reaches the supraoccipital (Fig. 3C, D). The medioventral part of the exoccipital laterally overlaps the basioccipital and forms a posteriorly directed process that forms the dorsolateral part of the condylus occipitalis (Fig. 2C, D; Brinkman et al., 2006). The exoccipital parts of the occipital condyle project further posteriorly than the medioventral basioccipital part (Fig. 2C, D). Although the basioccipital is expressed in the floor of the foramen magnum itself, the exoccipitals come to touch each other in a point contact along the dorsal surface of the occipital condyle (Fig. 3C, D; contra Brinkman et al., 2006). On the posterolateral surface, near the contact with the pterygoid, the exoccipital has two foramina nervi hypoglossi (Fig. 2C, D).

### Basioccipital

The basioccipital of TMP 1997.99.1 is complete (Figs. 2, 3, 5, 6). It is a small, median, unpaired, roughly triangular bone that contributes to the braincase posteroventrally (Fig. 2C, D). It consists of a main body, that floors the posteromedial braincase, and a posterior process that forms the condylus occipitalis. The main body of the basioccipital contacts the para basisphenoid anteriorly and the pterygoids laterally (Fig. 2C, D). The posterior process of the basioccipital contacts the exoccipitals along its lateral surface. The dorsal exposure of the main body of the basioccipital is roughly triangular and floors the posterior part of the braincase (Fig. 5). Together with the parabasisphenoid, it forms a high median ridge, the crista dorsalis basioccipitalis, that separates the base of the cavum cranii into two deep lateral pits (Fig. 6). The basis tuberculi basalis is located at the middle of the crista. Laterally, the basioccipital and parabasisphenoid jointly form the ventral margin of the hiatus acusticus. On the ventral surface, the basioccipital forms indistinct, rounded bulges that we interpret as the basioccipital tubercles (Fig. 2C, D). The tubercles are low and separated by a shallow, but wide median pit that is situated on the ventral surface of the basioccipital. This condition reminds of that of modern cheloniids, which, however, possess much deeper pits. In *Dermochelys coriacea*, the basioccipital tubercles are higher. The posterior process of the basioccipital of TMP 1997.99.1 contacts the exoccipitals dorsolaterally and forms the median third of the smooth and rounded occipital condyle (Fig. 3C, D). The dorsal border of the posterior process forms the ventral rim of the foramen magnum.

### Prootic

The prootic of TMP 1997.99.1 (Figs. 2, 3, 5, 6, 7, 9) is a paired bone situated in the anteromedial part of the otic capsule. This bone contacts the parietal anterodorsomedially, the supraoccipital posterodorsomedially, the quadrate posterolaterally, the opisthotic posteriorly, the pterygoid ventrally, and the parabasisphenoid medially (Fig. 6). The prootic has an anterolateral process that overlaps the anterior surface of the quadrate at the anterior end of the otic capsule, which, together with the quadrate forms the anteriorly bulged processus trochlearis oticum (Fig. 6). Posterior to the processus trochlearis oticum, the prootic forms the medial part of the foramen stapedio-temporale (Figs. 2A, B, 7C, D). The latter represents the opening of the canalis stapedio-temporale. This large canal, which is jointly formed with the quadrate, is deeply incised within the lateral surface of the prootic and is only capped laterally by the quadrate (Fig. 7C, D). The canal ends ventrally in a foramen, the aditus canalis stapedio-temporalis, which is jointly formed by the prootic and quadrate as the opening for the stapedial artery from within the cavum acustico-jugulare.

The prootic extends dorsomedially towards the parietal, contacting the processus inferior parietalis of the latter posterior to the trigeminal foramen (Fig. 6). This large, ovoid opening is formed posteriorly by the prootic, anteriorly by the parietal, and ventrally by the pterygoid (Fig. 6).

Posterior to the trigeminal foramen, the prootic forms the dorsal half of the foramen cavernosum (Fig. 6C, D), which constitutes the anterior opening of the canalis cavernosus that holds the lateral head vein (see pterygoid section), and which is medially, laterally, and dorsally framed by the prootic. The medial wall of the canalis cavernosus is part of the medioventral process of the prootic that contacts the parabasisphenoid and the pterygoid ventrally (Fig. 6). In the pterygoid–prootic contact, there is a large foramen that opens into the canal for the carotid artery (Fig. 9). As described in the pterygoid section above, we interpret this as an enlarged foramen pro ramo nervi vidiani. Dorsally above this enlarged foramen pro ramo nervi vidiani, there is the lateral opening of the facial nerve canal (Fig. 9), which projects mediolaterally through the prootic towards the medial wall of the prootic (Fig. 6). This medial wall of the ventral prootic process contributes to the lateral wall of the braincase and its surface shows a small yet distinct fossa called the fossa acustico-facialis (Figs. 5, 6). This fossa preserves two foramina (Fig. 5A, B). The anterior foramen, the foramen nervi facialis, connects to the canalis cavernosus via the aforementioned facial nerve canal. The posterior foramen within the fossa acustico-facialis, the foramen nervi acusticus, connects to the cavum labyrinthicum. Turtles generally are expected to show two additional acoustic foramina (e.g., Evers et al., 2019b; Ferreira et al., 2022) to accommodate the three nerve rami, but these are absent in the specimen TMP 1997.99.1. Their absence can likely be explained by a taphonomic or a segmentation artifact, as the foramina are often small and their separating bone struts are small and fragile.

Posterior to the fossa acustico-facialis, the prootic contributes to the hiatus acusticus, an irregular opening between the cavum labyrinthicum and the cavum cranii (Fig. 5). The prootic forms a deep cavity posteriorly that forms the anterior region of the inner ear cavities, which are otherwise formed by the opisthotic and supraoccipital. Laterally, toward the cavum acustico-jugulare, the prootic forms the anterior margin of the fenestra ovalis, to which the stapes would have articulated. The fenestra ovalis remains open posteroventrally, as the prootic and opisthotic have no contact in its ventral margin, which is thus exposed toward the pterygoid. Between the fenestra ovalis and internal, posterior opening into the canalis cavernosus, the prootic forms a posteriorly oriented surface that is part of the anterior wall of the cavum acustico-jugulare. This surface is excavated by a posterior prootic recess (e.g., Evers & Joyce, 2020), which can be easier appreciated on the right side.

### Opisthotic

The opisthotic of TMP 1997.99.1 (Figs. 2, 3, 5, 7) is a paired bone situated in the posteromedial part of the otic capsule. Anteriorly, the opisthotic contacts the prootic, laterally the quadrate, dorsally the supraoccipital (Fig. 2A, B), and posteriorly the exoccipital (Fig. 3C, D). The opisthotic does not contact the pterygoid ventrally. The opisthotic posterolaterally encloses the braincase and roofs the posterior region of the cavum acustico-jugulare (Fig. 3C, D).

The anterior wall of the opisthotic shows a deep and large recess (Fig. 6), which constitutes the posterior half of the cavum labyrinthicum. The opisthotic additionally forms an anteriorly recurved ventral process, the processus interfenestralis (Fig. 5), which separates the cavum labyrinthicum anteriorly from the recessus scalae tympani posteriorly. The fenestra perilymphatica forms a large foramen between both of these spaces in the medial part of the processus interfenestralis. The part of the opisthotic that is medial to the fenestra perilymphatica forms the anterior margin of the foramen jugulare anterius (Fig. 5). This part of the opisthotic does not contact the basioccipital ventrally. Laterally, the processus interfenestralis forms the posterior margin of the fenestra ovalis, which remains open ventrally. The processus does not contact the pterygoid ventrally, leaving a large hiatus postlagenum between the labyrinth and recessus scalae tympani. The base of the processus interfenestralis is laterally pierced by the foramen nervi glossopharyngei.

Posterolaterally, the opisthotic is prolonged into a paraoccipital process (Figs. 3C, D, 7E, F), that extends posteriorly to contact the quadrate and, likely, the squamosal (see squamosal). The paroccipital process contributes to the dorsal wall of the fenestra postotica (Fig. 3C, D), but is also strongly overlapped by the exoccipital, which somewhat reduces its surficial contribution to this large opening.

### Parabasisphenoid

The parabasisphenoid of TMP 1997.99.1 (Figs. 2, 3, 4, 5, 6, 8, 9) is an unpaired median bone that forms the anteroventral floor of the braincase. It contacts the pterygoid anterolaterally, the prootic laterally, and the basioccipital posteriorly (Fig. 6). The parabasisphenoid is composed of two principal structures, a central part with a dorsal surface called the parabasisphenoid cup that is bowl-like, and a rod-like and anteriorly directed process called rostrum basisphenoidale (Fig. 6). Ventrally, the parabasisphenoid has a small triangular exposure between the pterygoids and basioccipital (Brinkman et al., 2006).

The parabasisphenoid cup of TMP 1997.99.1 is relatively deep, as its surrounding walls are high. There is a thin dorsal crest, the crista tuberculi basalis, that traverses the parabasisphenoid cup in the midline, and which continues posteriorly on the basioccipital (Fig. 6; Brinkman et al., 2006). The rostrum basisphenoidale of TMP 1997.99.1 projects anteriorly from the parabasisphenoid cup as a long rod with a nearly circular cross-section (Figs. 5, 6, 8, 9; Brinkman et al., 2006). An internal suture of the rostrum basisphenoidale was reported by Brinkman et al. (2006), but we cannot confirm the presence of this suture based on the new CT scans. The rostrum basisphenoidale projects slightly dorsally with its anterior end such that its posterior half is embedded between the pterygoids, whereas its anterior half rest upon the interpterygoid contact dorsally (Figs. 5, 6). The rostrum basisphenoidale extends slightly anterior to the level of the anterior margin of the processus inferior parietalis (Fig. 4) and can thus be seen in lateral view. The trabeculae are only developed as extremely shallow, indistinct ridges on the dorsolateral aspect of the rostrum and are slightly stronger developed anteriorly (Fig. 6). The sella turcica usually is a fossa that invades the base of the dorsum sellae and rostrum basisphenoidale, in which the anterior foramina for the cerebral artery are situated and which holds the pituitary gland (e.g., Ferreira et al., 2022; Gaffney, 1979). In TMP 1997.99.1, the sella turcica is not developed as a fossa or depression (Fig. 6D). Instead, the anterior foramina for the cerebral artery (foramina anterius canalis caroticus cerebralis sensu Rabi et al., 2013; foramina anterius canalis caroticus basisphenoidalis sensu Rollot et al., 2021) are located at the intersection of the rostrum basisphenoidale and the dorsum sellae on an anterodorsally facing surface (Fig. 6D). Right and left foramina are separated by a thin strut of bone that projects slightly anteriorly as a weak ridge on the anterior surface of the dorsum sellae and are thus only minorly spaced across the midline (Figs. 6D, 9). Dorsally, the ridge between the anterior cerebral artery foramina continues as a median spike on the dorsal margin of the dorsum sellae (Figs. 6D, 9). The dorsum sellae itself is a steep and high, vertical wall of bone between the clinoid processes. These are developed as thin, steeply dorsolaterally projecting rods (Figs. 6, 9). Retractor bulbi pits are absent. Instead, the anterior foramina for the abducens nerve just open in the shallowly concave surface ventral to the base of each clinoid process (Figs. 6, 9). Posteriorly, the abducens canal traverses through the anterior wall of the parabasisphenoid and opens in the parabasisphenoid cup (Fig. 6D).

The rostrum basisphenoidale and sella turcica show much variation among pan-chelonioids. The sella turcica plesiomorphically is a relatively deep and broad fossa at the base of a broad, plate-like rostrum basisphenoidale. The trabeculae are quite distinct plesiomorphically and highlight the sella turcica further, and the anterior cerebral foramina are widely spaced and situated within the sella turcica. This plesiomorphic combination of traits can be observed in *Toxochelys latiremis* (e.g., AMNH 1042; Matzke, 2009). A broad, plated rostrum basisphenoidale is present in *Dermochelys coriacea*, but the trabeculae are reduced, and the cerebral foramina strongly modified. Extant cheloniids universally have a rod-like rostrum basisphenoidale and the sella turcica is reduced to a shallow, anteroposterior elongate but narrow groove, in which the anterior cerebral foramina are closely spaced or even convergent. In many ways, the rostrum basisphenoidale morphology of TMP 1997.99.1 thus appears to be derived with regard to *Toxochelys* spp., particularly in the clear formation of a rod-like rostrum, the reduction of the trabeculae, and the narrow midline spacing of the anterior cerebral foramina (Fig. 6; Brinkman et al., 2006). The strong reduction of a sella turcica may be autapomorphic to *Nichollsemys baieri*, based on the specimen TMP 1997.99.1.

The parabasisphenoid internally houses parts of the internal carotid arterial system (see also pterygoid and prootic sections) (Figs. 5, 6, 8; Brinkman et al., 2006). The internal carotid canal first comes in contact with the parabasisphenoid at the position of the large cavity between pterygoid, prootic, and parabasisphenoid that is described above (see pterygoid section) (Fig. 8). From here, the canal extends within the pterygoid-parabasisphenoid suture. Slightly more anteriorly, just posteriorly to the level of the clinoid process base, the internal carotid canal splits in two subordinate canals (Fig. 8). The more medial of these two canals corresponds to the canalis caroticus basisphenoidalis of Rollot et al. (2021), through which the cerebral branch of the carotid artery extends. This canal enters the parabasisphenoid and exits dorsally in the sella turcica at the foramen anterius canalis carotici basisphenoidalis, which is described above. The second canal extends further anteriorly within the pterygoid-parabasisphenoid suture and corresponds to the palatine artery canal (the canalis caroticus lateralis of Rollot et al., 2021) (Fig. 8). This is slightly smaller than the canal for the cerebral artery, as in *Toxochelys mooreviliensis* (FMNH PR 219). The palatine artery canal of TMP 1997.99.1 exits dorsally into the sulcus cavernosus as a foramen formed in the pterygoid-parabasisphenoid suture (foramen anterius canalis carotici palatinum of Rabi et al., 2013; foramen anterius canalis caroticus lateralis of Rollot et al., 2021), which is situated at the same level as the foramen anterius canalis carotici basisphenoidalis (see pterygoid section) (Figs. 6, 8). Thus, TMP 1997.99.1 has a fully developed internal carotid artery split into palatine and cerebral arteries (as in extant chelonioids), but this arterial system is fully embedded in bone. This embedding contrasts the condition of extant chelonioids, in which the undivided internal carotid artery exits into the sulcus cavernosus before it splits into the cerebral and palatine arteries, the latter of which is never covered by bone (Evers & Benson, 2019; Evers et al., 2019b; Zangerl, 1953b). Although this situation has been known since Zangerl (1953b), it was never well documented. Here, we can confirm that this arterial pattern is observable in extant cheloniids, as evidenced by a dice-CT scan of *Chelonia mydas* (UF herp 51413), which nicely shows the arterial pattern. The arterial pattern of TMP 1997.99.1 with a fully enclosed carotid split is the plesiomorphic condition, as it can also be observed in chelydrids (Albrecht, 1976; Rollot et al., 2021), as well as in *Toxochelys moorevillensis* (FMNH PR219). A covered split is further observable in in *Allopleuron hofmanni* (NMUK R4213), protostegids (Evers et al., 2019a), as well as Eocene cheloniids such as *Eochelone brabantica* (Evers & Benson, 2019; Evers et al., 2019a), such that the uncovered carotid split of extant cheloniids seems to be a derived feature that evolved fairly late within chelonioid evolution.

### Stapes

**Fig. 10:**
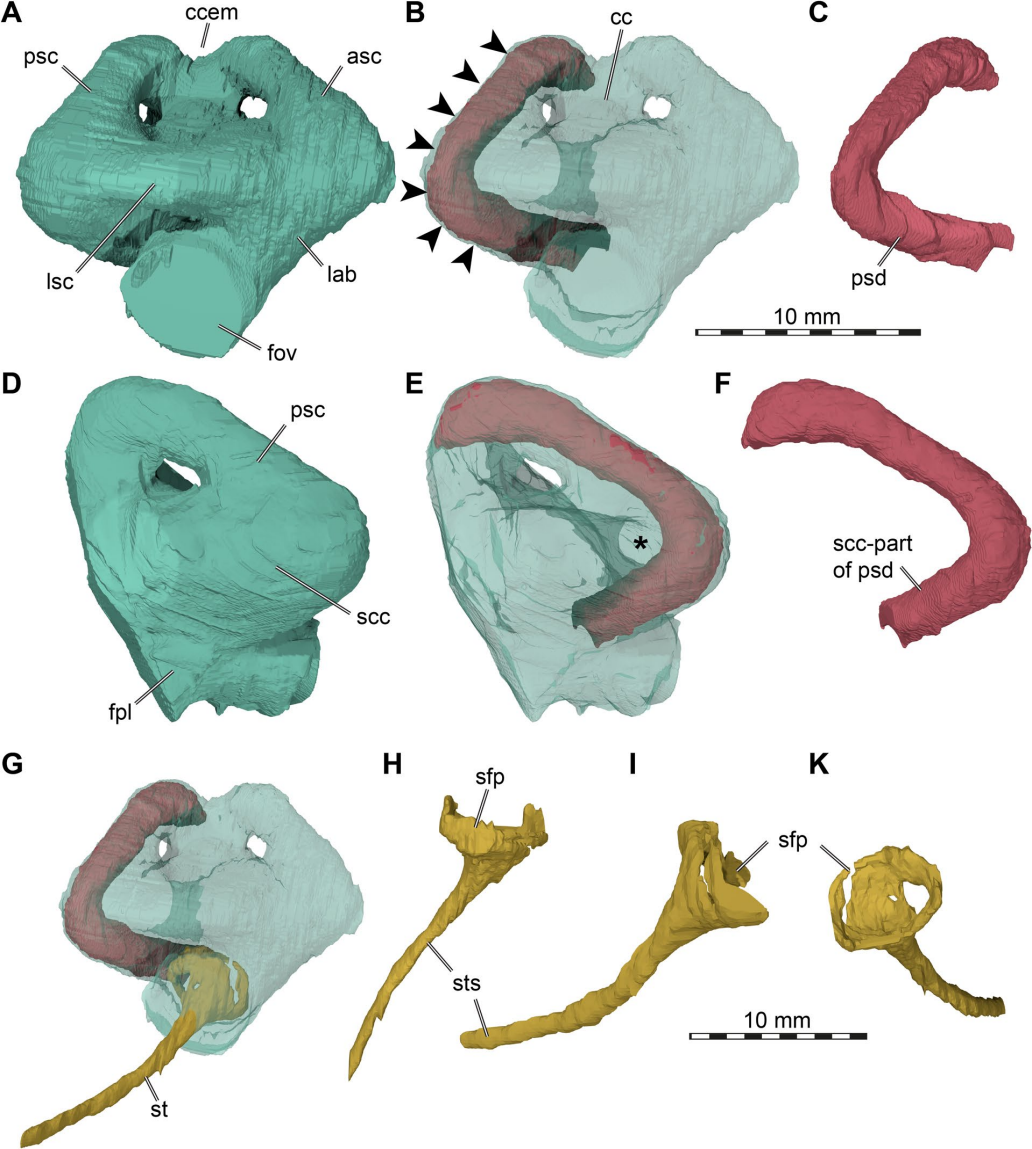
Three-dimensional renderings of right endosseous labyrinth, the stapes, and “soft tissue” preservation of the posterior semicircular duct of the holotype specimen of *Nichollsemys baieri* (TMP 1997.99.1). **A** lateral view of the endosseous labyrinth; **B** lateral view of the endosseous labyrinth rendered transparent with the preserved posterior semicircular duct in solid; **C** lateral view of the posterior semicircular duct; **D** posterior view of the endosseous labyrinth; **E** posterior view of the endosseous labyrinth rendered transparent with the preserved posterior semicircular duct in solid; **F** posterior view of the posterior semicircular duct; **G** lateral view of labyrinth structures rendered transparent to see the position of the stapes, which is slightly medially displaced through the fenestra ovalis; **H** stapes in dorsal view; **I** stapes in anterior view; **K** stapes in medial view. Arrows in **B** show the attachment of the posterior semicircular duct to the outer (dorsal) wall of the semicircular canal. Asterisk in **E** shows the level of the lateral semicircular canal to highlight how the "soft tissue" bends ventrally around it. *Asc* anterior semicircular canal, *cc* common crus, *ccem* common crus embayment, *fov* fenestra ovalis, *fpl* fenestra perilymphatica, *lab* endosseous labyrinth, *lsc* lateral semicircular canal, *psc* posterior semicircular canal, *psd* posterior semicircular duct, *scc* secondary common crus, *sfp* stapedial footplate, *st* stapes, *sts* stapedial shaft

The medial portions of both stapes of TMP 1997.99.1 are preserved (Fig. 10G–K). They are in approximately their original position though slightly pushed into the labyrinth cavity on each side (Fig. 10G). As in other turtles, the stapes has the shape of a trumpet with a medially placed stapedial footplate attached to a thin shaft that connects with the tympanic membrane via the cartilaginous extrastapes (e.g., Foth et al., 2019; Rollot et al., 2024) (Fig. 10). In TMP 1997.99.1, the stapedial footplate is nearly circular and medially excavated by a deep recess (Fig. 10K). The rim of the stapedial footplate is delicate, but slightly thicker in the dorsal aspect (Fig. 10H) compared to the anterior, ventral, and posterior region. The bone forming the stapedial footplate is very thin, which makes it hard to segment it consistently, as in some slices the bone is only a few voxels thick. In TMP 1997.99.1, as currently segmented, both stapedial footplates are penetrated by irregular but fairly large openings (Fig. 10K). It is unclear if any of these represent genuine morphology, or if they are segmentation or taphonomic artefacts of the thin bone layer forming this part of the stapes.

Laterally, the stapes continues as a very thin rod-like shaft, which is notably curved. (Fig. 10H–I). Here, the curvature is such that the stapedial rod bows downwards in the central area of the cavum acustico-juglare. On each side, the stapedial shaft is incomplete, and broken before entering the incisura columellae auris.

### Endosseous and partial membranous labyrinth

We segmented the right endosseous labyrinth of TMP 1997.99.1. In addition, we noticed a dark halo is apparent inside parts of the endosseous labyrinth cavity, which seems to extend through parts of the posterior semicircular canal. Interestingly, segmentation of this halo suggests that the structure in question might represent “soft tissue” preservation of the membranous posterior semicircular duct, as we argue below.

As in most turtles, the endosseous labyrinth cavity of TMP 1997.99.1 is comprised of a series of intersecting canals formed by the prootic, opisthotic, and supraoccipital (Evers et al., 2019b; Ferreira et al., 2022). The vertical semicircular canals are nearly symmetrical (Fig. 10A), as is typical for turtles (Evers et al., 2022a). Again, as in other turtles, many details of the underlying membranous labyrinth morphology are poorly reflected in the endosseous endocast (Evers et al., 2019b, 2022a; Ferreira et al., 2022), as the ampullae of TMP 1997.99.1 are poorly visible and as the intersection of the posterior and lateral semicircular canal form a large confluent cavity, the secondary common crus (Fig. 10D). Only the lateral ampulla is visible as a thickening toward the anterior end of the lateral semicircular canal (Fig. 10A). The lateral semicircular canal is gently bowed outwards along its course, whereas the vertical semicircular canals are relatively straight in their mid-sections (Fig. 10A). Toward the common crus, the anterior and posterior semicircular canals become strongly curved ventrally, such that their intersection in the common crus describes a strong dorsal embayment (Fig. 10A). Such embayments have been observed and described for a range of different extant and fossil turtles (e.g., Evers et al., 2019b, 2021; Joyce et al., 2021c; Lautenschlager et al., 2018; Martín-Jiménez & Péréz-García 2023a, 2023b, 2023c; Rollot et al., 2021; Smith et al., 2023), although they are not always as deep as in TMP 1997.99.1 and also not universally present among turtles (e.g., Evers et al., 2019b, 2022a; Lautenschlager et al., 2018). Interestingly, the protostegid *Rhinochelys pulchriceps* has a similarly deep dorsal embayment (Evers et al., 2019b), whereas embayments are entirely absent in most fossil and extant crown chelonioids (e.g., *Argillochelys cuneiceps*, *Lepidochelys olivacea*, *Dermochelys coriacea*, *Allopleuron hofmanni*; Evers et al., 2019b, 2022a), or much shallower (e.g., *Puppigerus camperi*, *Eochelone brabantica*; Evers et al., 2022a). However, another early protostegid, *Bouliachelys suteri*, lacks a deep common crus embayment (Evers et al., 2022a).

The fenestra ovalis of TMP 1997.99.1 has a lateral orientation (Fig. 10A) that is seen in most turtles, including chelydrids and protostegids. This contrasts with the derived condition of crown chelonioids, in which the fenestra ovalis is posteroventrally oriented (e.g., Evers et al., 2019b). The fenestra perilymphatica shows as a relatively small, roundish opening at the posterior end of the endosseous labyrinth and below the semicircular canals (Fig. 10D).

The structure that might represent “soft tissue” preserved remnants of the posterior semicircular duct extends through the entire endosseous posterior semicircular canal of TMP 1997.99.1, including the common crus region dorsally, and into the secondary common crus ventrally (Fig. 10B, C, E, F). Within the secondary common crus region, the duct curves posteroventrally below the horizontal plane of the lateral semicircular canal (Fig. 10E, F). This course matches the expectation of the posterior semicircular duct, which curves posteroventrally around the lateral semicircular duct in extant turtles (Evers et al., 2019b, 2022a; Ferreira et al., 2022). The posterior semicircular duct of TMP 1997.99.1 has a smaller internal diameter than the semicircular canal, whereby the outer aspect of the duct membrane seems attached to the outer wall of the semicircular canal (Fig. 10B), creating perilymphatic spaces toward the inner perimeter of the posterior semicircular canal (Fig. 10B, E). This, again, is expected for a membranous semicircular duct, as these are suspended from the outer semicircular canal walls in extant reptiles (Baird, 1970), which has also been observed and figured for extant turtles (Evers et al., 2019b). The internal diameter of the posterior semicircular duct of TMP 1997.99.1 is unusually large, and much larger than the internal diameters that have been reported for membranous turtle labyrinths (Evers et al., 2019a, 2019b; Ferreira et al., 2022), including sea turtles (Evers et al., 2022a). The functional interpretations of this are discussed below.

### Potential arterial “soft tissue” preservation

The sedimentary matrix of the nodule in which TMP 1997.99.1 is preserved is not homogeneous, but rather traversed by likely burrows of varying internal diameters and varying grey value differences to the surrounding matrix in the CT slices. Some of the potential burrows show as lighter colored structures, whereas other are darker. Most of these potential burrows have a seemingly random spatial distribution and traverse the internal hollows of the fossil from all directions. However, we noticed that a few of these structures follow the expected course of arterial structures. Particularly, there is a continuous system of structures within the bony canals of the internal carotid artery system that potentially represent “soft tissue” preservation of the carotid arteries. This inference is supported by the approximate right-left symmetry of structures to either skull side (Supplementary File 1: Fig. S2). However, it is also possible that these structures arise from bioturbators that follow the path of the arterial canals. Besides tracing the internal carotid artery canal, the palatine artery canal and the cerebral artery canal (Supplementary File 1: Fig. S2), the proposed “soft tissue” system shows an additional, posterior branch of the internal carotid artery, which extends through the floor of the cavum acustico-jugulare (Supplementary File 1: Fig. S2). This posterior branch is asymmetric on both skull sides, as the left sided structure has an additional subbranch we could not find in the right one (Supplementary File 1: Fig. S2). Yet, the proposed “soft tissue” system additionally shows an arterial branch in the area of the enlarged foramen pro ramo nervi vidiani (Supplementary File 1: Fig. S2). On the right side, this branch is clearly exiting this foramen as well as the trigeminal foramen into the adductor fossa (Supplementary File 1: Fig. S2). On the left side, this branch is shorter and less clearly associated with the foramen pro ramo nervi vidiani (Supplementary File 1: Fig. S2). Although we are careful in inferring any novel arterial conclusions from these observations, they at least provide tentative additional evidence for the “soft tissue” preservation we infer for the membranous labyrinth.

### Dentary

**Fig. 11:**
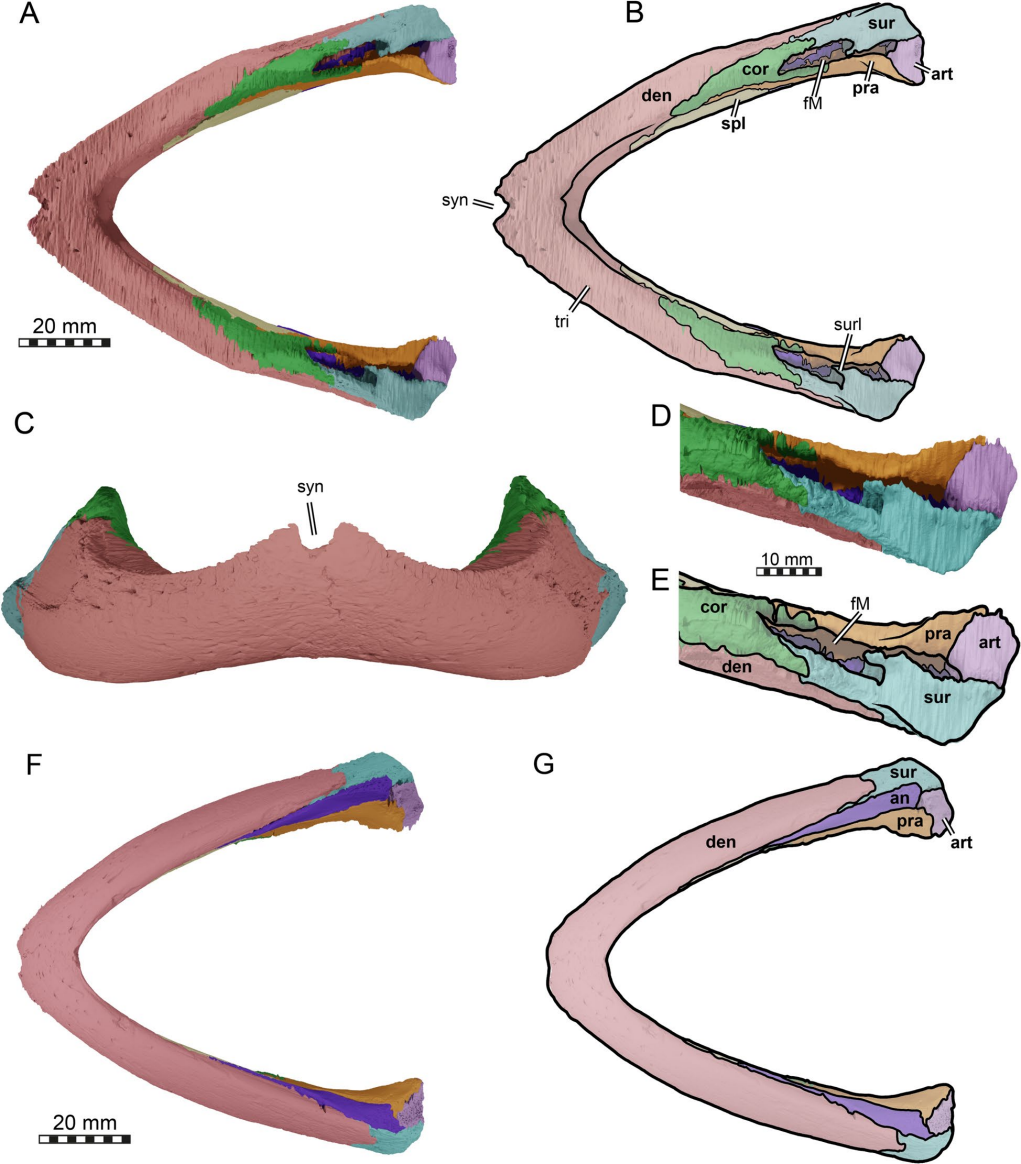
Three-dimensional renderings of the mandible of the holotype specimen of *Nichollsemys baieri* (TMP 1997.99.1). **A** dorsal view; **B** interpretative line-drawing; **C** anterior view;** D** close-up onto the posterior part of the left mandibular ramus in dorsal view; **E** interpretative line-drawing; **F** ventral view; **G** interpretative line-drawing. Note that bones are labelled in bold and features labelled in regular font. *An* angular, *art* articular, *cor* coronoid, *den* dentary, *fM* fossa Meckelii, *pra* prearticular, *spl* splenial, *sur* surangular, *surl* surangular lamina, *syn* symphyseal notch, *tri* triturating surface

**Fig. 12:**
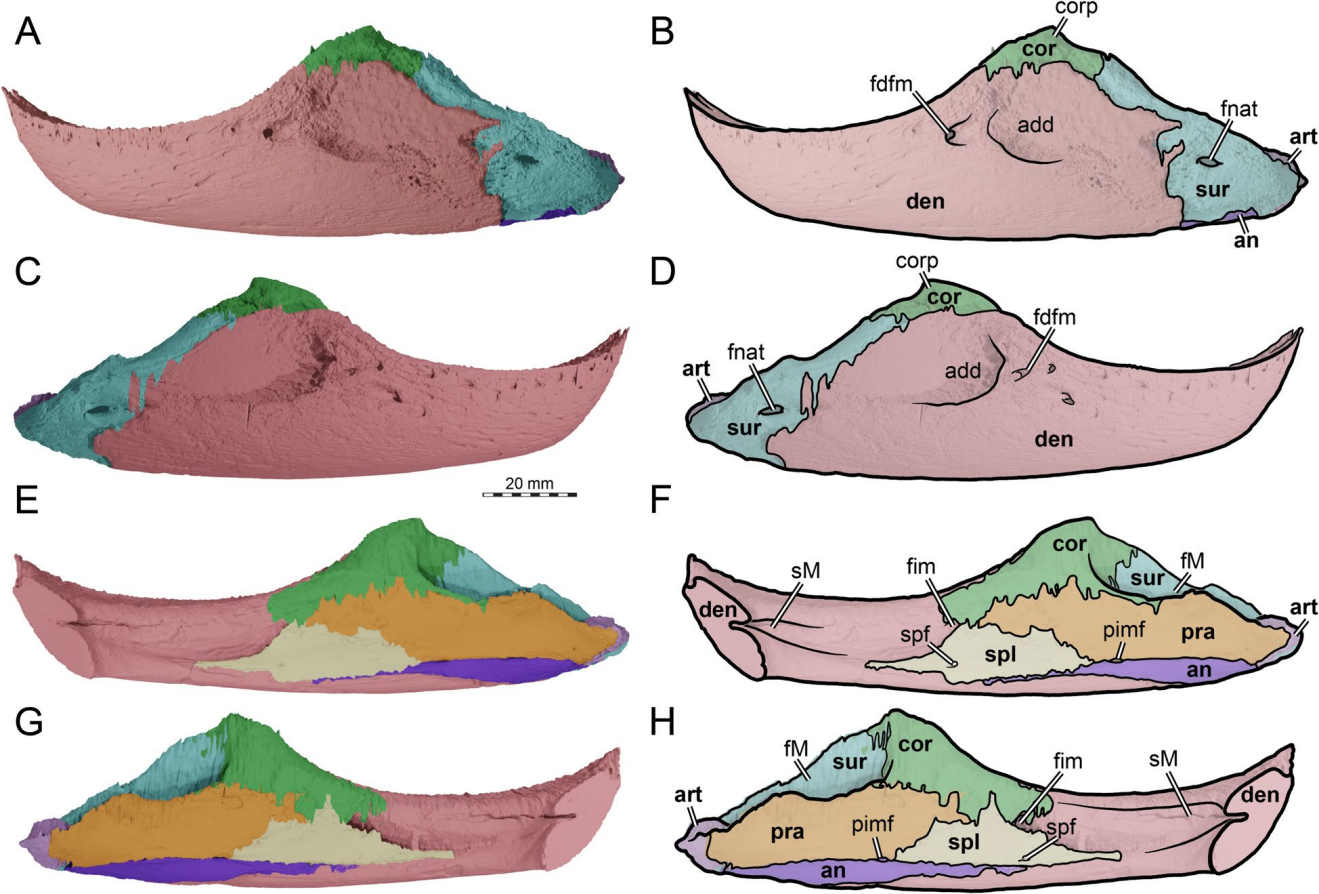
Three-dimensional renderings of the mandible of the holotype specimen of *Nichollsemys baieri* (TMP 1997.99.1). **A** left lateral view; **B** interpretative line-drawing; **C** right lateral view; **D** interpretative line-drawing; **E** medial view onto right mandibular ramus; **F** interpretative line-drawing; **G** medial view onto left mandibular ramus; **H** interpretative line-drawing. Note that the dentary was artificially cut at the symphysis to get undisturbed views onto the medial mandibular sides. Also note that bones are labelled in bold and features labelled in regular font. *an* angular, *art* articular, *cor* coronoid, *corp* coronoid process, *den* dentary, *fdfm* foramen dentofaciale majus, *fim* foramen intermandibulare medius, *fM* fossa Meckelii, *fnat* foramen nervi auriculotemporalis, *pra* prearticular, *sM* sulcus Meckelii, *spf* splenial foramen, *spl* splenial, *sur* surangular

The dentary of TMP 1997.99.1 (Figs. 11, 12) is well preserved. The dentary is a large, unpaired bone resulting from the fusion of the left and right dentaries, which is seen in all cryptodires (e.g., Evers et al., 2022b; Gaffney, 1979). The dentary of TMP 1997.99.1 contacts the coronoid, the surangular, the angular, and the splenial. Contacts with the prearticular and articular are absent (Figs. 11, 12). The dentary represents the main part of the lower jaw and dominates the mandible in lateral view, overlapping most of the coronoid and surangular, such that only a small band of the latter is visible dorsally along the lateral margin of the dorsal foramen into the fossa Meckelii (Fig. 12A–D). Posteriorly, the dentary overlaps the surangular up to a level shortly before the foramen auriculotemporalis. This jaw configuration is similar in *Toxochelys latiremis* (Matzke, 2009) but in contrast to that of modern cheloniids, in which the surangular has a large lateral exposure and an anteriorly directed process that separates the dentary into a posterodorsal and posteroventral process (Evers et al., 2022b).

The dentary rami of TMP 1997.99.1 are robust and anteroposteriorly elongated processes like in all pan-chelonioids. In contrast to extant cheloniids (Evers et al., 2022b), but similar to *Toxochelys latiremis* (Matzke, 2009), the dentary rami of TMP 1997.99.1 retain approximately the same width across their length and show no expansion of the triturating surfaces toward the symphysis (Fig. 11A, B; Brinkman et al., 2006). The anterior tip of the dentary in the symphyseal area is deeply notched dorsally, whereas the notch is laterally framed by a small spike-like projection to either side (Fig. 11A–C). This morphology is highly unusual and not seen in any other turtle that we are aware of, but we are confident that it is genuine, as there are no external signs of damage, as the spike-like projects are symmetric, and as the mandible was found in articulation with the cranium. There is no trace of a median symphyseal ridge in TMP 1997.99.1 (Fig. 11A–B). The triturating surfaces are moderately narrow and decorated by a low, but sharp labial ridge (Fig. 11A–C; Brinkman et al., 2006). The lingual ridge is absent, but the lingual margin is nevertheless raised enough for the triturating surfaces to form a slightly depressed dorsal platform (Fig. 11A, B; Brinkman et al., 2006). The lingual margin is rounded toward the medial (= lingual) surface of the dentary (Fig. 12E–H). The triturating surface bears a line of anteroposteriorly aligned, large neurovascular foramina (Fig. 11A, B). In addition, such foramina of various sizes cover the lateral surface of the dentary anterior to the coronoid process and adductor fossa. They are particularly dense close to the labial margin of the jaw (Figs. 11C, 12A–D).

In lateral view, the dentary is anteriorly high at the symphysis, becomes lower in central parts of the dentary ramus and then becomes slightly higher again toward the low coronoid process (Fig. 12A–D). The adductor fossa is well imprinted onto the lateral dentary surface below the externally visible articulation area with the coronoid (Brinkman et al., 2006). The adductor fossa is ventrally bound by a relatively strong ridge (Fig. 12A–D; Brinkman et al., 2006). The foramen dentofaciale majus is located just anterior to the distinct adductor fossa (Figs. 12A–D). Although still a large opening, the foramen dentofaciale majus of TMP 1997.99.1 is smaller than in many extant cheloniids (Evers et al., 2022b). This is possibly because there is a smaller, additional foramen just anterior to the ‘major’ foramen dentofaciale majus, which also opens into the canalis alveolaris inferior. The canalis alveolare inferior has a very large diameter in TMP 1997.99.1, and can be traced anteriorly until the symphyseal notch, where it penetrates the surrounding bone. Posteromedially, the canal opens into the large foramen alveolare inferius, which is concealed in medial view by the prearticular and splenial where it opens into the fossa Meckelii. The fossa Meckelii anteriorly becomes a shallow sulcus cartilaginis Meckelii on the lingual surface of the dentary (Fig. 12E–H). It becomes shallower toward the symphysis, where the left and right sulci meet below the lingual margin of the symphysis. In addition, there is a fine, narrow, yet deep groove directly ventral to the lingual margin. The groove is coalescent between right and left dentary rami, and continues posteriorly until approximately half the anteroposterior length of each dentary ramus (Figs. 12E–H).

### Surangular

The surangular of TMP 1997.99.1 is a paired bone that is situated at the posterior end of the lateral side of the mandible (Figs. 11, 12). It has two principal structures: an anterodorsally directed process that contributes to the dorsal foramen into the fossa Meckelii, and a posterior part that is included in the jaw articulation surface. The surangular contacts the dentary anteriorly, the angular ventromedially, the articular posteriorly, the coronoid anterodorsally, and possibly the prearticular medially along its recurved lamina (Figs. 11, 12).

The anterodorsal processes of the surangular forms the lateral margin of the dorsal foramen into the fossa Meckelii (Fig. 11A–E). This lateral margin of the foramen extends dorsally much higher than its medial margin (as formed by the prearticular), resulting in a broad medial exposure of the surangular and a dorsomedial orientation of the foramen into the fossa Meckelii (Fig. 12E–H). This is unusual for chelonioids, as the lateral and medial margins are equally high in extant cheloniids (Evers et al., 2022b). At the posterior end of the fossa Meckelii and the base of its anterodorsal process, the surangular of TMP 1997.99.1 shows a strong anteromedially directed recurved lamina (Evers & Benson, 2019) (Fig. 11D, E). In the fossil, the lamina comes close to the lateral wall of the fossa Meckelii as formed by the prearticular, but an actual contact is just about absent (Fig. 11D, E). However, given how close both bones come, the contact may well be inferred based on external observation of the specimen. The recurved lamina of TMP 1997.99.1 encases a deep anteromedial recess of the surangular, which extends to the fossa Meckelii posterolaterally. At the posterior surface of the fossa, there is a large auriculotemporal canal, which extends posterolaterally through the surangular and opens on the lateral surface below the level of the articular surfaces as a small and anteroposteriorly elongated foramen auriculotemporalis (Fig. 12A–D). A dorsal surangular foramen is absent. The foramen auriculotemporalis is absent in *Dermochelys coriacea* and extant cheloniids (Evers et al., 2022b), but appears in protostegids (e.g., *Rhinochelys pulchriceps*; Evers et al., 2019a; *Protostega gigas*: FMNH P27385) and in *Toxochelys latiremis* (Matzke, 2009)*.*

Posteriorly to the fossa Meckelii, the surangular becomes laterally expanded, forming a roughly crescentic, posterodorsally exposed surface that forms the anterolateral part of the area articularis mandibularis (Fig. 11D, E). The lateral expansion is caused by a moderately wide ectocondylar flange (Evers et al., 2022b), which is well rounded. This flange overhangs the foramen auriculotemporalis laterally. The articular surface of the surangular is nearly flat, but angled slightly laterally, so that the medial margin that is facing the prearticular and articular bones is gently raised. This creates a subtle division of ecto- (lateral) and endocondylar (medial) subfacets (Fig. 11D, E), which are common in cryptodires (Evers et al., 2022b).

### Coronoid

The coronoid of TMP 1997.99.1 (Figs. 11, 12) is a paired bone situated at the dorsal end of the coronoid process. It contacts the dentary anteroventrally and laterally, the splenial anteromedially, the prearticular posteromedially, and the surangular posterolaterally (Figs. 11, 12).

The coronoid consists of a central part that is dorsally raised to a low, rounded coronoid process (Fig. 12A–D). The process only extends dorsally slightly beyond the dentary, so that most of the coronoid is concealed in lateral view. Posteriorly, the coronoid forms a medial and a lateral process which arches over the fossa Meckelii to contact the prearticular and surangular, respectively (Fig. 11D, E). The coronoid thus forms the anterior rim of the dorsal foramen into the fossa Meckelii. Whereas the posterolateral process is relatively short, the posteromedial one extends for nearly half the length of the foramen along the side of the prearticular (Fig. 11D, E).

The coronoid of TMP 1997.99.1 has a well-developed, relatively long anteroventral process (Fig. 12E–H), which extends along the posteromedial surface of the dentary above the level of the fossa Meckelii, thereby minutely contributing to the triturating surfaces. Together with the splenial and dentary, the coronoid forms the foramen intermandibularis medius, i.e., the anterior opening of the fossa Meckelii (Fig. 12E–H). A coronoid foramen is absent in TMP 1997.99.1.

### Angular

The angular of TMP 1997.99.1 (Figs. 11, 12) is a paired, elongate bone situated ventrally at the posterior half of the medial wall of the mandibular ramus. It meets the splenial anterodorsally, the prearticular dorsally, the surangular posterolaterally, the dentary ventrolaterally, and the articular posteriorly (Figs. 11, 12). The angular floors the fossa Meckelii, thereby prohibiting a dentary–prearticular contact (Fig. 11D, E). The posterior part of the angular is mediolaterally broader than the anterior parts of the bone, which becomes extremely thin and rod-like (Figs. 11F, G, 12E–H). The posterior end of the bone is more or less horizontally oriented (Fig. 11F, G), underlapping the ventral margins of the surangular, articular, and prearticular. The contact with the articular is small, so that much of the latter bone was ventrally exposed and not covered by the angular. As the angular begins to taper anteriorly, it becomes medially twisted until it lies vertically within the medial wall of the mandibular ramus (Fig. 12E–H). In this area, it is underlapped by the dentary, so that it has no ventral exposure (Fig. 11F, G). Close to the triple junction with the splenial and prearticular, the angular forms the ventral margin of a relatively small posterior intermandibular foramen, which is dorsally closed by the prearticular (Fig. 12E–H). Posterior intermandibular foramina are completely reduced in most extant cheloniid species (Evers et al., 2022b), but are clearly present in *Toxochelys latiremis* (e.g., AMNH 14221).

### Prearticular

The prearticular of TMP 1997.99.1 (Figs. 11, 12) is a paired, vertically oriented sheet of bone in the posterior part of the medial side of the mandibular ramus. The prearticular contacts the splenial anteroventrally, the coronoid anterodorsally, the articular posteriorly, the angular ventrally, and likely the surangular (Figs. 11, 12). The prearticular has no contact with the dentary. The anterior end of the bone forms parts of the medial wall of the fossa Meckelii (Fig. 11D–E). As a splenial is present in TMP 1997.99.1, the prearticular only has a single, anterodorsally directed process (Evers et al., 2022b) (Fig. 12E–H). Its dorsal margin forms parts of the medial margin of the dorsal foramen into the fossa Meckelii, but its contribution to this margin is reduced by a moderately long posteromedial process of the coronoid (Fig. 11D–E). Along the contact with the angular, the prearticular forms the posterior intermandibular foramen (Fig. 12E–H). A prearticular foramen is absent. Just anterior to the articular surface of the mandible, the prearticular nearly contacts the surangular along its vertical, recurved lamina (see surangular) (Fig. 11D–E). Posterior to this near contact, the prearticular and surangular enclosed a posterior, pocket-like expansion of the fossa Meckelii. This cavity is posteriorly closed by the articular, which is wedged between the prearticular and surangular. The articular of TMP 1997.99.1 lacks an anterior articular process, which is present in many turtles although it seems to ossify very late in ontogeny (Evers et al., 2022b). It is thus possible that the posterior prolongation of the fossa Meckelii housed the cartilaginous anterior end of the articular of TMP 1997.99.1. The prearticular participates in the formation of the anteroventral part of the articular facet of the mandible in the form of a small flange that is medially expanded and becomes wider posteroventrally (Fig. 11D–E).

### Splenial

The splenial of TMP 1997.99.1 (Figs. 11, 12) is a paired, vertically oriented, sheet-like bone that forms the anterior part of the medial wall of the fossa Meckelii. The splenial meets the coronoid dorsally, the prearticular posterodorsally, the angular posteroventrally, and the dentary anteroventrally (Figs. 11, 12). The posterior part of the splenial is sheet-like, filling the entire space between angular and prearticular. Anterior to the prearticular, the splenial has a short contact with the coronoid (Fig. 12E–H), which differs from the long splenial–coronoid contact of chelids (Evers et al., 2022b), plesiochelyids (Gaffney, 1976), pleurosternids (Evers, 2023), baenids (Gaffney, 1982), helochelydrids (Joyce et al., 2014), but also from the plesiomorphic condition of turtles in which this contact is entirely absent (*Proganochelys quenstedtii*: Gaffney, 1990). Anterior to its coronoid contact, the splenial of TMP 1997.99.1 gradually reduces its height (Fig. 12E–H). The resulting anterodorsal border of the splenial forms the posteroventral margin a the large and anteriorly directed foramen intermandibularis medius, which is the opening from the fossa Meckelii into the sulcus Meckelii. The anterior splenial process continues anteriorly in the ventral margin of the sulcus Meckelii, where it contacts the dentary ventrally (Fig. 12E–H). There is a clearly developed splenial foramen near the base of the anterior process (Fig. 12E–H). This foramen is also seen in those specimens of *Toxochelys latiremis* which clearly preserve a splenial (e.g., AMNH 1496; Supplementary file 1: Fig. S3). Splenials are absent in extant chelonioids and have also not been reported to occur in *Toxochelys latiremis* (Matzke, 2009). However, we identified several specimens in which splenials are undoubtedly present (AMNH 1496, AMNH 14221, AMNH 5118, YPM 3609, YPM 3611, Supplementary file 1: Fig. S3). It seems possible that previous descriptions of *Toxochelys latiremis* (i.e., Matzke, 2009) have overlooked the suture between angular and splenial. For instance, AMNH 5119 is figured in Matzke (2009: Fig. 13), and the angular in the interpretative line drawing increases its height anteriorly, which is unusual for angulars generally (Evers et al., 2022b). Then, the anterior end becomes a thin process, and there is a clearly developed foramen associated with the base of this process. The morphology of the anterior part of the angular in Matzke (2009) thus mirrors the splenial morphology of TMP 1997.99.1. Thus, it seems likely that splenials are plesiomorphically present within pan-chelonioids. Besides in TMP 1997.99.1 and *Toxochelys latiremis*, splenials also occur in early protostegids (e.g., *Rhinochelys pulchriceps*: Evers et al., 2019a; *Santanachelys gaffneyi*: pers. obs. SWE), and in *Allopleuron hofmanni* (Mulder, 2003), which is usually interpreted as a Cretaceous crown group chelonioid.

### Articular

The articular of TMP 1997.99.1 (Figs. 11, 12) is a small, posteriorly situated, paired bone that mostly forms the area articularis mandibularis, i.e., the articular facet with the quadrate. The left articular is complete, but the right one is abraded posteroventrally. The articular contacts the surangular anterolaterally, the prearticular anteromedially, and the angular anteroventrally (Figs. 11, 12). The articular is positioned between the posterior processes of the prearticular and surangular (Fig. 11F–G), where it closes the posterior extension of the fossa Meckelii that lies between these bones. The articular of TMP 1997.99.1 lacks an anterior process, which may have been formed as a cartilaginous process within this recess. The articular is a bone that ossifies late in ontogeny, resulting in the common absence of an ossified anterior process in many turtles (Evers et al., 2022b).

The area articularis mandibularis is the dorsally exposed surface of the articular (Fig. 11D–E). In TMP 1997.99.1, this surface of the articular contributes to roughly one fourth of the total articulation area, with one half formed by the surangular and another fourth by the prearticular (Fig. 11D–E). Along the contact with the surangular, the articular is slightly raised, resulting in a posterior tubercle that finishes the articular surface posteriorly, and separates it from the small, lip-like retroarticular process. This is as in most pan-chelonioids, in which an elongate retroarticular process is absent, with the exception of some Late Cretaceous protostegids such as *Terlinguachelys fischbecki* (Lehman and Tomlinson, 2004). Posterior chorda tympani foramina are absent in the articular (or elsewhere) of TMP 1997.99.1.

### Cornu branchiale I

**Fig. 13:**
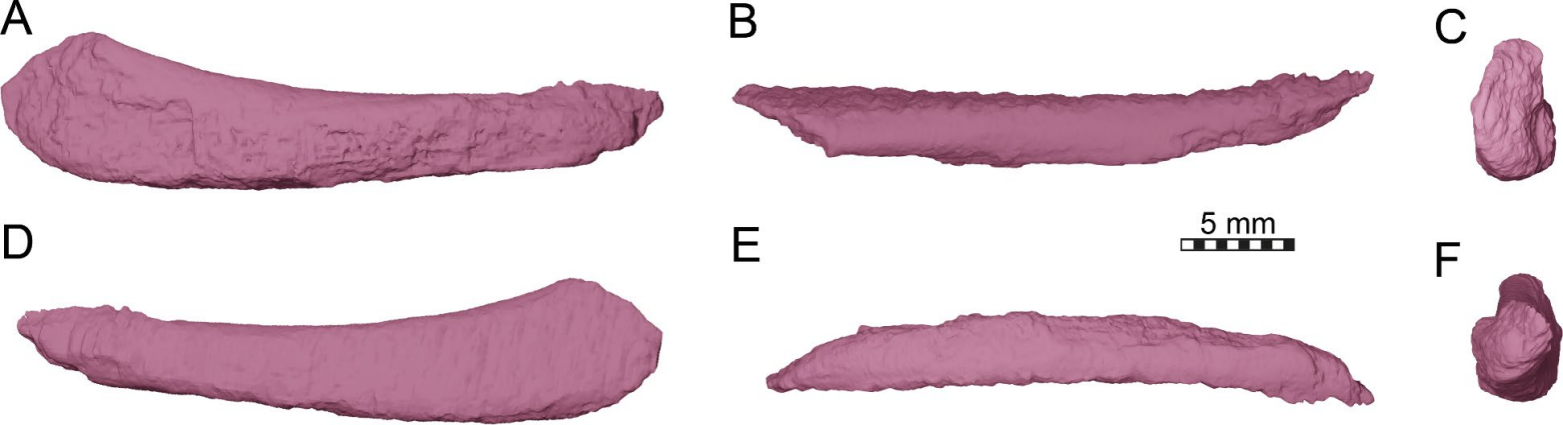
Three-dimensional renderings of the left cornu branchiale I of the holotype specimen of *Nichollsemys baieri* (TMP 1997.99.1). **A** external lateral view; **B** dorsal view; **C** anterior view; **D** medial lateral view; **E** ventral view;** F** posterior view

The preserved record of the hyoid apparatus of TMP 1997.99.1 consists only of a fragment of one hyobranchial element still floating in the matrix (Figs. 1C, 13). The position of this element close to the left quadratojugal could mean that it formerly belonged to the cornu branchiale I, probably its anterior part. However, it is also possible that this element is not preserved in its original anatomical position. For the purpose of this description, we use directional terms (e.g., “lateral”) with regard to the currently preserved position of the element.

The element consists of a slightly curved rod (Fig. 13), which has a thinner posterior end with a near circular cross-section, and a dorsoventrally broader but mediolaterally thinner anterior end with an elliptical cross-section. This anterior expansion is often observed in cornu branchiale I of turtles (e.g., Matzke, 2009; Mulder, 2003; Siebenrock, 1898). A specimen of *Toxochelys latiremis* (AMNH 5118) that preserves the first hyobranchial was previously reported (Matzke, 2009; Zangerl, 1953a) and seems to show a similar morphology as in TMP 1997.99.1. However, due to the deformation that this specimen has undergone, it is difficult to confirm. Another record for this element occurs in NHMM 000001, a specimen of *Allopleuron hofmanni*, and shows a more squarish lateral profile for its anterior tip (see pl. 19 in Mulder, 2003).

## Phylogeny

**Fig. 14:**
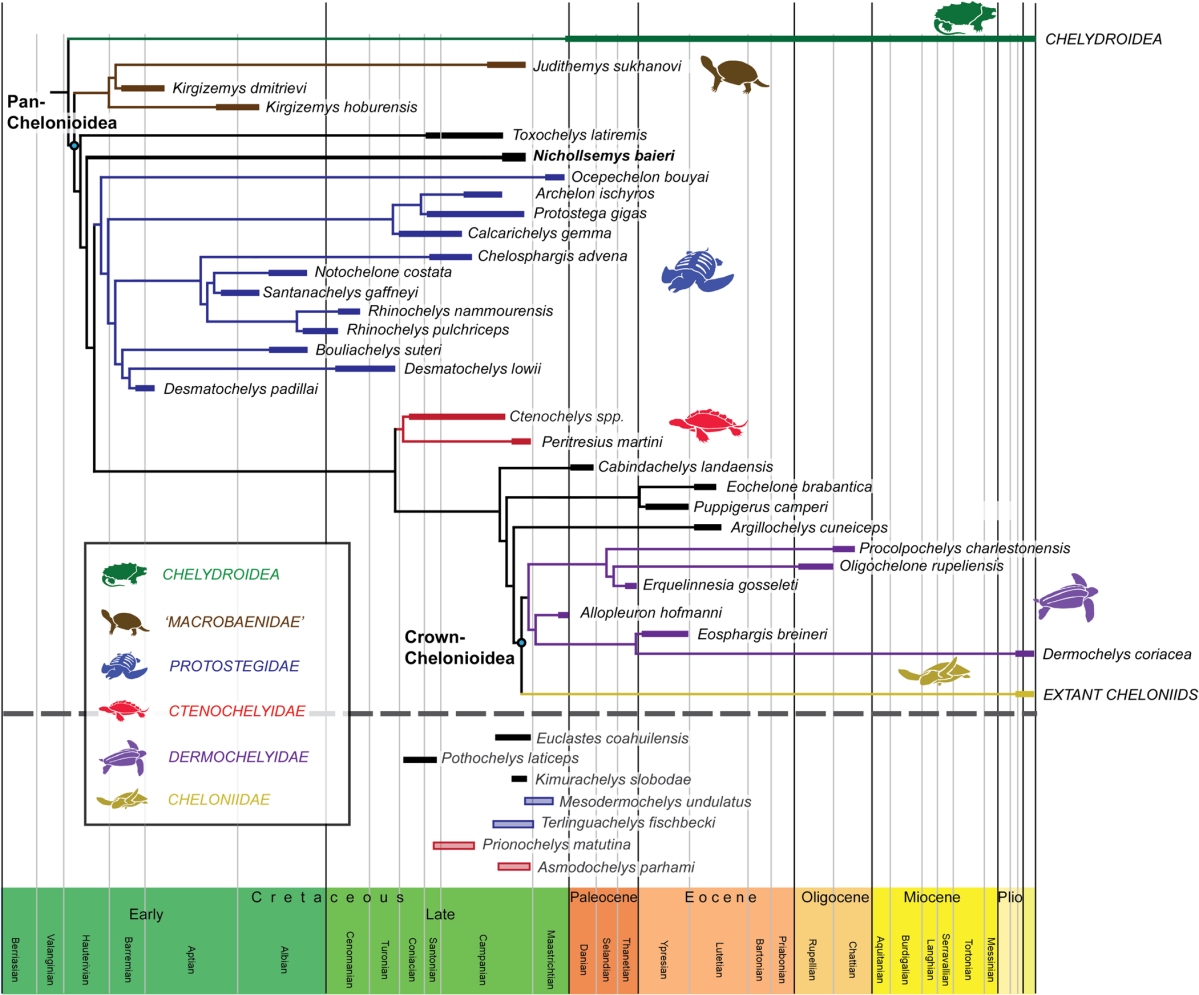
Strict consensus tree obtained from 2 MPTs from our implied weighting analysis using ordered characters based on our fully revised matrix plotted through time. Note that only the pan-chelonioid part of the tree is shown (full tree available as Supplementary file S5). Thick bars on terminal branches represent stratigraphic occurrences of focal taxa. Note that some additional taxa not included in our phylogeny are also plotted below the dotted gray line to highlight the diversity of Late Cretaceous sea turtles. Silhouettes by Juliette Menon and Walter Joyce

For our first phylogenetic analysis, we use the version of the matrix of Joyce et al. (2021a) for which only the scorings of *Nichollsemys baieri* had been updated. This analysis, which was performed with equal weighting and without ordering characters, resulted in 432 MPTs of 1720 character-state transitions (Supplementary file S4). The analysis of the second matrix, in which scorings of more taxa were corrected, and which was analysed using implied weighting and with ordering characters that form morphoclines, resulted in 2 MPTs with a tree length of 69.4 (Supplementary file S5). This implies 1727 character-state transitions. Maybe unexpectedly, both analyses agree on the principal topological results (Supplementary file S1: Figs S4–S5), which are (Fig. 14): (1) *Nichollsemys baieri* is recovered as a stem chelonioid (contrary to Joyce et al., 2021a); (2) *Nichollsemys baieri* is not the sister-taxon of *Toxochelys latiremis* (contrasting Brinkman et al., 2006), but instead it is placed one node more crownward than *Toxochelys latiremis*; (3) protostegids are found to be stem chelonioids in a more crownward position than *Nichollsemys baieri* (as in Joyce et al., 2021a); (4) some “macrobaenids” (*Kirgizemys* spp., *Judithemys sukhanovi*) are found as the earliest-branching stem chelonioids (as in Joyce et al., 2021a), although in different compositions among our two analyses (Supplementary file S1: Figs S4–S5). Thus, regarding early sea turtle relationships, our study primarily differs from our baseline matrix of Joyce et al. (2021a) in the placement of *Nichollsemys baieri* itself, which is removed from the chelonioid crown group. However, our analyses imply strong topological differences to Joyce et al. (2021a) in more crownward parts of the tree than protostegids. Specifically, both our analyses agree in (5) retrieving ctenochelyids as stem chelonioids one node more crownwardly than protostegids (contrasting previous studies that find them as stem cheloniids, e.g., Joyce et al., 2021a, Gentry et al., 2018; but as in Gentry, 2018, 2019) (Fig. 14); (6) recovering Eocene fossils from central Europe that are classically viewed as stem cheloniids (*Argillochelys, Puppigerus, Eochelone*; e.g., Moody, 1974; Gaffney, 1979; Evers & Benson, 2019; Gentry et al., 2019) as stem chelonioids in a node more crownward than ctenochelyids (Fig. 14); (7) finding “*Euclastes*-like turtles”, typified in our sample by the Eocene *Erquelinnesia gosseleti* as early branching members of the stem lineage of *Dermochelys coriacea* (Fig. 14); (8) finding *Allopleuron hofmanni* as a dermochelyid, placed between turtles of the *Erquelinnesia*-group and *Eosphargis breineri* (as in Gentry et al., 2019) (Fig. 14). Our two analyses differ primarily in the ingroup relationships of protostegids (Supplementary file S1: Figs S4–S5), which are not our primary concern here. Regarding the aforementioned “macrobaenids”, our two analyses differ in the interpretation of *Dracochelys bicuspis*, an Early Cretaceous taxon from Asia (Supplementary file S1: Figs S4–S5). This species is frequently recovered as a sinemydid turtle (e.g., Joyce et al., 2021a; Zhou & Rabi, 2015), or at least closely related to *Sinemys* spp. in a clade that contains xinjiangchelyids, sinemydids, and “macrobaenids” (e.g., Joyce et al., 2016). “Macrobaenids” are phylogenetically defined by *Macrobaena mongolica*, which has so far not been included in phylogenetic analyses. Nevertheless, various associations of turtles have frequently been referred to as “macrobaenids”, and these usually minimally include *Judithemys sukhanovi* and *Kirgizemys* spp. These turtles are also consistently found in close-by phylogenetic nodes in our two analyses (Supplementary file S1: Figs S4–S5). The implied weighting analysis of the more heavily modified matrix finds these turtles in a clade (i.e., a monophyletic “Macrobaenidae”; Fig. 14, Supplementary file S1: Fig. S4). In the equally weighted analysis of the matrix that includes only revised scorings for *Nichollsemys baieri*, these turtles do not form a clade in all MPTs (Supplementary file S4), but form various paraphyletic grades on the early stem lineage of chelonioids, consistently in a more stemward position than *Toxochelys* (Supplementary file S1: Fig. S4). However, in addition to this, some MPTs recover *Dracochelys bicuspis* as another taxon among this grade, whereas yet other MPTs find it as a sinemydid, as in the implied weighting tree of the more heavily modified matrix version (Supplementary file S4). As in Joyce et al. (2021a), both of our analyses do not find a close relationship between sinemydids (defined by the species *Sinemys lens*), which are found to be stem turtles, and “macrobaenids”.

Overall, although our two analyses use different methodologies, results outside of protostegids and “macrobaenid” ingroup relationships strongly agree. This indicates that the downweighing of homoplasy (i.e., implied weighting) and ordering of morphoclines has no strong impact on large parts of the chelonioid tree, which is also evident by the small change in character-state changes that implied weighting and ordering characters implies (1727) when compared to equal weighting without ordering (1720). This is despite some surprising topological results, which we consider to be evolutionarily unlikely scenarios that are instead probably driven by homoplasy (e.g., *Erquelinnesia* as a dermochelyid, Eocene fossils as stem chelonioids). In the following, we use our character optimizations to outline the morphological support for various clades of interest and to scrutinize our results. For this, we use the implied weighting topology, which recovers a monophyletic “Macrobaenidae” consisting only of *Judithemys sukhanovi* and *Kirgizemys* spp. (i.e., not *Dracochelys bicuspis*). Full synapomorphy lists for both of our analytical procedures can be found in Supplementary files S6–7.

Our *Pan-Chelonioidea* (including macrobaenids; Fig. 14) is supported by 25 synapomorphies, 13 of which are unambiguous (Supplementary file S7). In addition, there are ten ACCTRAN and three DELTRAN optimizations (see supplementary list of apomorphies). The unambiguous synapomorphies include features that are generally quite variable in turtles and which appear homoplastically also in various other groups, such as a dorsolateral orientation of the orbits (ch. 14.1), the absence of a jugal contact with palatine (29.0), the squamosal forming the posterodorsal margin of the cavum tympani (41.0), a pterygoid contribution to the foramen palatinum posterius (ch. 97.0), a large external pterygoid process with anterolateral contacts to the maxilla (ch. 101.0), ventrally exposed hypoglossal nerve (CN XII) foramina (ch. 118.0), or a coalescent foramen jugulare posterius with the fenestra postotica (ch. 165.1). However, the list also includes characters that are less homoplastic, such as a squamosal-postorbital contact (36.0) that is present despite a relatively large temporal emargination (19.1), or the T-shape of the entoplaston (ch. 246.2).

*Toxochelys latiremis* and more crownwardly positioned pan-chelonioids (Fig. 14) are united by 38 synapomorphies, of which 22 are unambiguous, ten resulting from ACCTRAN and six from DELTRAN optimizations (Supplementary file S7). This node does not only have high numerical support, but many of the unambiguous synapomorphies provide robust morphological support, because many are only weakly homoplastic and many represent adaptations to a marine lifestyle. For example, the group is supported by a wide-open squamosal-quadrate contact (40.1), the presence of a V-shaped crest in the ventral parabasisphenoid surface (137.1), a high dorsum sellae (ch. 141.2), a close spacing of the foramina anterius canali carotici cerebralis (ch. 144.1). For the postcranium, many shell characters are related to the reduction of ossification that is typical of sea turtles (even if it homoplastically appears in other lineages, e.g., among trionychids): the presence of costo-peripheral fontanelles (212.1), the presence of a central plastral fontanelle (236.1), a reduced hyo-hypoplastral contact (240.0), the presence of strong serrations along hyo- and hypoplastra facing other bones (241.1), the absence of an ossified axillary buttress (252.1). Additionally, the part of the pan-chelonioid tree that includes *Toxochelys latiremis* and more crownward sea turtles is supported by characters related to flipper evolution (e.g., Evers et al., 2019a; Joyce et al., 2021a; Motani & Vermeiji, 2021), including a distal displacement of the lateral process along the humeral shaft (332.2), a humerus that is longer than the femur (ch. 337.1), the absence of movable interdigital articulations in the third to fifth manual digits (ch. 342.1), the flattening of carpal and tarsal elements (ch. 350.1), the positioning of the radius anterior to the ulna (ch. 356.1). Among the ACCTRAN characters, there are also some additional traits are that are typical for extant marine turtles, and which may have evolved as early as this node. In the postcranium, these include the presence of a raised pedestral on the visceral side of the nuchal for the articulation of the eighth cervical vertebra (ch. 196.1), the adult retention of a posterior plastral fontanelle (ch. 237.1), and the secondary reduction of recurved postzygapophyses on the eighth cervical vertebra (ch. 295.0).

*Nichollsemys baieri* and more crownwardly positioned pan-chelonioids (Fig. 14) are united by 18 synapomorphies, two of which are unambiguous, whereas 14 are recovered under ACCTRAN and one under DELTRAN (Supplementary file S7). Among these characters, the unambiguous synapomorphy of having a rod-like rostrum basisphenoidale (ch. 140.2) can be seen as a relatively strong morphological support, because it appears in nearly all chelonioids (and also in protostegids). Although nasals are generally absent in cryptodires, the plesiomorphic presence of nasals in pan-chelonioids is well documented by *Toxochelys latiremis*, which clearly possesses nasals and which clearly is an early stem chelonioid. *Nichollsemys baieri* is more advanced than *Toxochelys latiremis* in this regard, as it has already lost its nasals. In our optimization, the absence of nasal (ch. 1.1) is returned as an ACCTRAN synapomorphy, and this is caused by the more crownward position of protostegids, which do have nasals. Similarly, chelonioids lack elongate squamosal processes, which are plesiomorphically present in *Toxochelys latiremis*. *Nichollsemys baieri* documents the loss of these processes, but the respective character state transition (ch. 39.0) is not found as an unambiguous synapomorphy but as an ACCTRAN transition due to the presence of long processes in *Ocepechelon bouyai*, which is recovered as an early protostegid in our tree. Most of the other ACCTRAN synapomorphies for the node that includes *Nichollsemys baieri* and more crownward pan-chelonioids are postcranial characters that currently cannot be evaluated for *Nichollsemys baieri*.

Protostegids and more crownwardly positioned pan-chelonioids (Fig. 14) are united by 25 synapomorphies, five of which are unambiguous, five of which are ACCTRAN and 15 of which are DELTRAN (Supplementary file S7). Features that unite these turtles include those related to the reduction of emarginations, such as an absent or weak upper temporal emargination (ch. 19.0), and the absent or weakly developed lower temporal (= cheek) emargination (ch. 35.0) that is common to most pan-chelonioids and protostegids. Another unambiguous synapomorphy is the absence of foramina praepalatina (ch. 50.0), which is generally rare among turtles but otherwise found in other secondarily marine turtles with secondary palates, such as sandownids (Evers & Joyce, 2020). Some of the unambiguous synapomorphies returned for this clade are susceptible to high homoplasy, such as the presence of a dentary symphyseal ridge (176.1), which is variably absent or present among both protostegids (Evers et al., 2019a; Wieland, 1900) and crown chelonioids (e.g., Chatterji et al., 2021; Jones et al., 2012). The ACCTRAN optimizations include, for example, the absence of a splenial (ch. 187.1), which is present in protostegids and but absent in most chelonioids (but see *Allopleuron hofmanni*).

Ctenochelyids and more crownwardly positioned pan-chelonioids (Fig. 14) are united by 20 synapomorphies, ten of which are unambiguous, six of which are ACCTRAN and four of which are DELTRAN (Supplementary file S7). Some of the unambiguous synapomorphies of this node are related to the formation of various degrees of a “secondary palate”, and include a large proportion of the triturating surface being composed of the palatine (ch. 56.1) and the vomer (ch. 69.1). In addition, the dentary experiences an enlargement of the foramen dentofaciale majus (ch. 178.1), and rib-free peripherals are present in the carapace (ch. 216.1).

Crown group chelonioids (Fig. 14) are united by nine synapomorphies, two of which are unambiguous, three of which are ACCTRAN and four of which are DELTRAN (Supplementary file S7). This number is relatively low in comparison to some of the other discussed nodes. This can probably be explained by the taxonomically restricted crown group obtained in our analyses, according to which many fossil taxa traditionally interpreted as crown chelonioids are excluded. The number could well be larger, if ctenochelyids and European Eocene “cheloniids” are in the future found as crown group chelonioids again. Currently, the two unambiguous synapomorphies of the crown group are a median contact of the exoccipitals in the floor of the foramen magnum (ch. 117.0; absent in *Chelonia mydas*) and the presence of an anterior surangular process that extends into the dentary (ch. 179.1).

Dermochelyidae (i.e., “*Erquelinnesia*”-group, *Allopleuron*, *Eosphargis*, *Dermochelys*; Fig. 14) are united by seven synapomorphies, two of which are unambiguous, four of which are ACCTRAN and one of which is DELTRAN (Supplementary file S7). Our taxonomic composition of Dermochelyidae is highly unusual, certainly unexpected, and requires caution. Specifically, the “*Erquelinnesia*” group includes turtles like the poorly known *Oligochelone rupeliensis*, the Oligocene *Procolpochelys charlestonensis*, which has been described as a stem cheloniid (e.g., Weems & Brown, 2017), and *Erquelinnesia gosseleti*. The latter is one of the best-known fossil taxa of a presumed group of turtles that are characterized by highly developed secondary palates, and which otherwise include taxa summarized in the waste-basket name “*Euclastes*”. Thus, parts of our Dermochelyidae include various taxa that are in part poorly understood and commonly interpreted as stem cheloniids. Maybe unsurprisingly or reassuringly, the unambiguous synapomorphies of this clade can be considered as relatively weak morphological support of this topology. This is because both characters are highly variable, even among sea turtles (e.g., Hirayama, 1992): The composition of the triturating surface with only a labial ridge (ch. 59.0) and the ectepicondylar opening being a fully enclosed canal (ch. 330.0; rather than a groove). In addition, having only a labial triturating ridge is also present in many cheloniids, such that this character does not seem to be particularly relevant although being returned as an unambiguous synapomorphy according to our topology.

Nevertheless, our Dermochelyidae also includes a clade composed of *Eosphargis breineri* as the immediate sister taxon to *Dermochelys coriacea*, and *Allopleuron hofmanni* as the next more stemwardly placed dermochelyid (Fig. 14). This topology has been found before, for example in Gentry et al. (2019). Whereas *Eosphargis breineri* is always found as a dermochelyid, the position of *Allopleuron hofmanni* is potentially more contested, with the few previous studies that included it finding it in as a stem cheloniid (e.g., Evers & Benson, 2019; Evers et al., 2019a; Joyce et al., 2021a). The clade is united by twelve synapomorphies, five of which are unambiguous, six of which are ACCTRAN and one of which are DELTRAN (Supplementary file S7). Among the unambiguous and ACCTRAN synapomorphies, there are several characters that provide relatively strong support for this topology, because these characters are rarely observed in other turtles. Some of these are only recovered as ACCTRAN, because the exact state is unknown from *Eosphargis breineri*. Among the cranial characters, we consider the presence of a jugal-squamosal contact (ch. 26.0; ACCTRAN), the absence of a postorbital-quadratojugal contact (ch. 43.1; ACCTRAN) and the strong reduction of a supraoccipital crest (ch. 112.0; ACCTRAN) as solid morphological character support. The presence of a jugal-squamosal contact does not imply the absence of a postorbital-quadratojugal contact, because this contact can occur on the medial surface of the jugal and squamosal. In addition, the strong size reduction of the foramen dentofaciale majus in the dentary is also a conspicuous character, especially as this foramen is hypertrophied in size in cheloniids (Evers et al., 2022b). Regarding the postcranium, the *Allopleuron*-including node is supported by hexagonal posterior costals (ch. 215.1; unambiguous) and a continuous keel on the neurals (ch. 191.1; ACCTRAN), which both appear in *Allopleuron hofmanni* and *Eosphargis breineri*, but are later lost entirely in subsequent dermochelyid evolution. In addition, these turtles share the absence of a cervical scute on the nuchal (ch. 224.1; unambiguous). However, the clade is also supported by two characters that arguably have a wider distribution, and two characters that result from non-sensical character optimization due to inapplicability scores. The first of the character that provide only weak morphological support is that the dorsal orbital margin is continuously concavely curved and lacks the “eyebrow” process seen in many cheloniids (ch. 45.0; umambiguous), whereas the second one is a size-reduced processus trochlearis oticum (ch. 80.1). The recovered unambiguous synapomorphy of having an anteromedial coronoid process (ch. 188.1) results from this observation in *Allopleuron hofmanni* alone, as the mandible for *Eosphargis breineri* is unknown and as the mandible of *Dermochelys coriacea* lacks a coronoid (Evers et al., 2022b; Nick, 1912) such that this character is scored as inapplicable. In the postcranium, the ACCTRAN synapomorphy of having a pygal notch is affected by the same type of problem with optimizing inapplicable characters, as *Dermochelys coriacea* lacks a pygal (Nick, 1912). The only DELTRAN optimization for this node is the absence of plastral scutes (263.1), the condition of which is unknown for the three turtles included in our *Erquelinnesia*-group.

## Discussion

### The timing of sea turtle origins and the ‘protostegid problem’

Although many aspects of pan-chelonioid phylogeny are instable, there is a broad consensus that *Toxochelys* spp. are stem chelonioids. This is strongly supported by their morphology, which displays a mixture of plesiomorphic and derived chelonioid features, and is reflected in phylogenetic studies, which universally recover this result (e.g., Anquetin, 2012; Anquetin et al., 2015; Cadena & Parham, 2015; Danilov & Parham, 2008; Evers & Benson, 2019; Gaffney & Meylan, 1988; Gentry et al., 2018; Joyce, 2007; Joyce et al., 2016; Sterli, 2010; Sterli et al., 2013; Zhou & Rabi, 2015). *Toxochelys* spp. are known from numerous specimens and two (Nicholls, 1988) or three nominal species (Hart Carrino, 2007; see Methods), and all known material is from the Santonian to Campanian of North America (Hay, 1905; Matzke, 2008, 2009; Nicholls, 1988; Zangerl, 1953a). This puts a temporal constraint on early sea turtle evolution, which is broadly consistent with molecular divergence time estimates for chelonioids, which suggest that the crown group originated sometime between the early Campanian (Late Cretaceous) and the early Eocene (Ypresian) (Joyce et al., 2013; Near et al., 2005; Pereira et al., 2017; Thomson et al., 2021).

A common problem of pan-chelonioid phylogeny, however, is the inclusion of additional, older (Early Cretaceous or even older) groups of turtles into the stem lineage of chelonioids, particularly protostegids (Evers et al., 2019a; Gentry et al., 2019; Joyce et al., 2021a; Raselli, 2018), which have sometimes even been found as part of the crown group (Cadena & Parham, 2015; Evers & Benson, 2019; Hirayama, 1995; Tong & Meylan, 2013). Protostegidae is a globally distributed clade of marine turtles, which is commonly interpreted to include early forms from the Early Cretaceous (Valanginian–Aptian) and derived, highly pelagic forms from the Late Cretaceous (Campanian) (e.g., Cadena & Combita-Romero, 2023; Cadena & Parham, 2015; Collins, 1970; Evers et al., 2019a; Elliott et al., 1997; Hirayama., 1998; Hooks, 1998; Kear & Lee, 2006; Raselli, 2018; Scavezzoni & Fischer, 2018; Zangerl & Sloan, 1960). In addition, “macrobaenids”, sinemydids, xinjiangchelyids, sandownids, or plesiochelyids are also sometimes retrieved as stem chelonioids (albeit in more stemward positions than *Toxochelys* spp.) (e.g., Anquetin et al., 2015; Cadena & Parham, 2015; Danilov & Parham, 2008; Gentry et al., 2019; Joyce et al., 2021a; Sterli, 2010; Tong & Meylan, 2013; this study). Any potential expansion of the chelonioid stem group by Early Cretaceous or older taxa results in long ghost lineages within crown Cryptodira and complicated biogeographic scenarios (Evers & Benson, 2019; Gentry et al., 2019; Zhou & Rabi, 2015). Despite these temporal inconsistencies, morphological support for a pan-chelonioid affinity of protostegids is usually strong and not primarily caused by convergence of ‘marine’ characters (Evers & Benson, 2019). Given that the position of protostegids is highly debated, given it is possible that they are an independent radiation of marine turtles (e.g., Anquetin, 2012; Anquetin et al., 2015; Danilov & Parham, 2008; Joyce, 2007; Sterli, 2010; Sterli et al., 2013), and given that their old age strongly suggest that they would be a very stemward taxon on the pan-chelonioid lineage in case they were pan-chelonioids (Gentry et al., 2019), *Toxochelys* spp. and closely related taxa are the key to understanding the early morphological evolution of modern sea turtles.

### *Nichollsemys baieri* as a transitional fossil in early sea turtle evolution

**Fig. 15:**
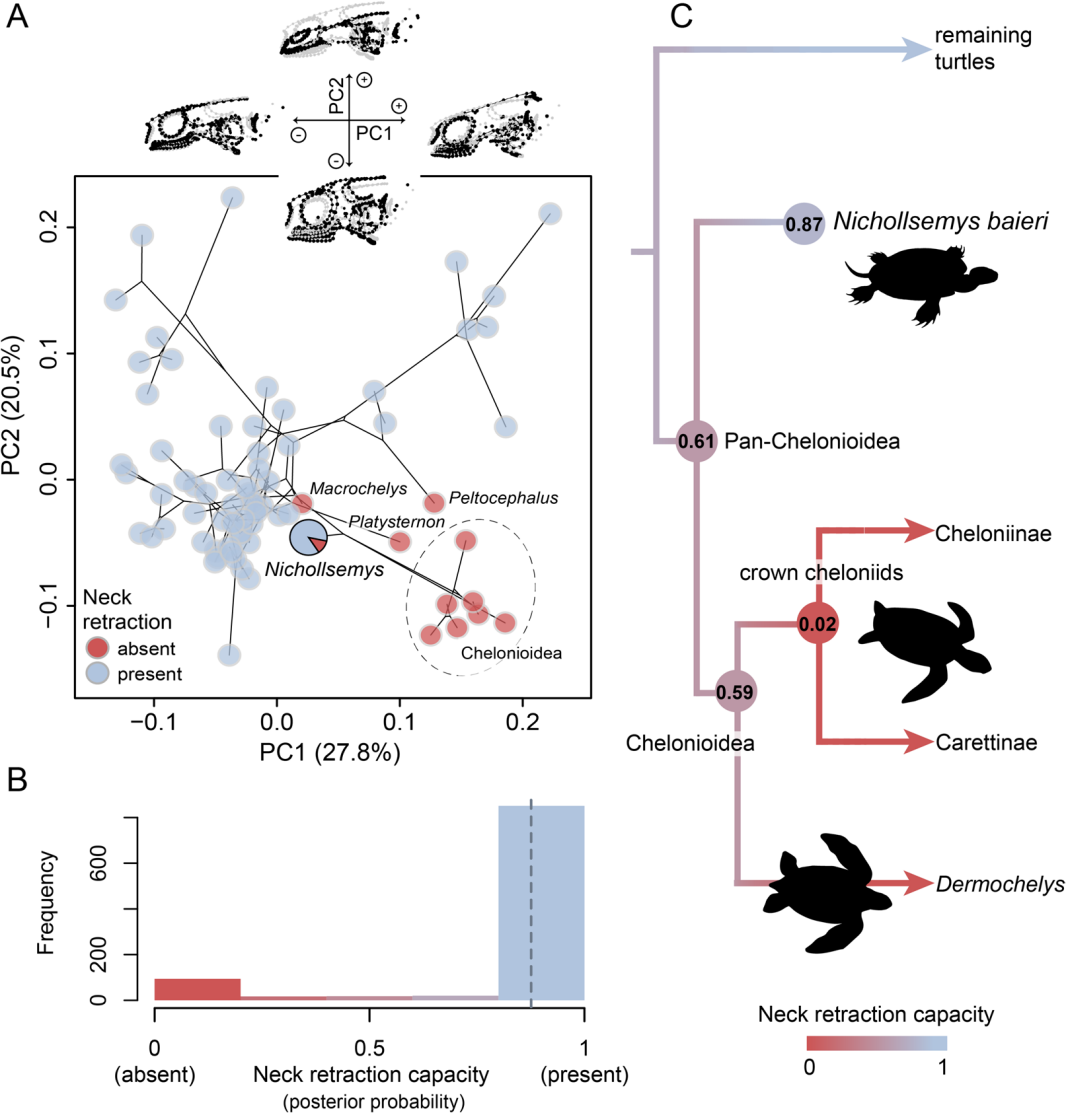
Morphospace position of *Nichollsemys baieri* and predictions of neck retraction. **A** Cranial morphospace of turtles, showing first two axes of a PCA. *Nichollsemys* is highlighted as a pie chart, indicating the probability of being capable of neck retraction predicted by pfda. Constellation of points above the plot indicates the consensus shape (gray) and extremes of the first two PCA axes (black) in left lateral view. **B** Histogram of posterior probabilities for the presence of neck retraction in *Nichollsemys baieri* across the set of pfda replicates. Dashed line indicates the mean value (0.86). **C** Ancestral reconstructions of neck retraction mapped onto a simplified phylogenetic tree of pan-chelonioid turtles. Numbers at nodes represent the ancestral probability of neck retraction capacity for pan-chelonioids (0.61), crown chelonioids (0.59) and crown cheloniids (0.02). *Chelonia mydas* silhouette by Edwin Price, and *Dermochelys coriacea* silhouette by Guillaume Dera, both retrieved from phylopic.org (under CC0 1.0)

Chelonioids are cryptodires and thus share many anatomical features with other representatives of this group, such as the presence of a processus trochlearis oticum, the contact of the vomer with the prefrontal, or the posterior expansion of the pterygoid (Evers & Benson, 2019; Gaffney, 1979; Joyce, 2007). However, chelonioids are also derived in many anatomical features, including differences in palate architecture, emargination depth, details of the basicranial anatomy, and features of the inner ear (e.g., Chatterji et al., 2022; Evers et al., 2019b, 2022a; Gaffney, 1979; Hirayama, 1995; Jones et al., 2012). *Toxochelys* spp. show a mosaic of generalized cryptodiran and derived chelonioid features that reinforces its phylogenetic position as an early stem chelonioid (e.g., Brinkman et al., 2006). Unfortunately, the fossil record of this taxon includes many strongly flattened or otherwise deformed or poorly preserved cranial specimens (Matzke, 2009), and attempts to CT scan skull material of *Toxochelys* spp. reveal that the rock composition of these fossils does not return good contrast between fossil bone and matrix (Evers, 2021). As a consequence, some details of the internal morphology remain unknown for *Toxochelys* spp. Our re-investigation of the holotype of *Nichollsemys baieri* firmly establishes this Campanian species from Canada as an additional early stem chelonioid (Brinkman et al., 2006; but contra phylogenetic results in Evers et al., 2019a; Joyce et al., 2021a). We show that it is positioned in a similar part of the pan-chelonioid tree as *Toxochelys* spp., but we find it in a slightly more crownward position than *Toxochelys* spp. This result makes sense relative to its morphology, because *Nichollsemys baieri* not only shares with *Toxochelys* spp. many symplesiomorphic features found in cryptodiran outgroups (Brinkman et al., 2006), but also a relatively large number of derived chelonioid features that are not apparent in *Toxochelys* spp. PCA ordination of skull landmarks supports the intermediate position of *Nichollsemys baieri* between non-sea turtles and chelonioids, as it plots at the edge of chelonioid morphospace and overlapping with other cryptodires (Fig. 15A). The mosaic of plesiomorphic and derived anatomical features, and the relative differences to *Toxochelys* spp. provide novel insights into successive character evolution on the paraphyletic stem lineage of chelonioids.

For example, *Toxochelys* spp. and *Nichollsemys baieri* both share with non-chelonioid cryptodires the presence of foramina praepalatina, the presence of a large foramen palatinum posterius, the ‘regular’ embedding of the internal carotid arterial split, and the presence of posterior intermandibular foramina. Some plesiomorphic features of *Toxochelys* spp. and *Nichollsemys baieri* have functional importance. These include the presence of a simple vertical ridge and single fossa on the posterior squamosal process, the presence of deep cheek and upper temporal emarginations and likely the presence of an elongate crista supraoccipitalis, the presence of a large processus pterygoideus externus with a well-developed maxillary contact and a large lateral flange, a large posterolateral expansion of the dentary within each mandibular ramus, and a narrow symphysis. These are all related to feeding biomechanics as well as neck mobility and retraction (Werneburg, 2011, 2015; Jones et al., 2012; Werneburg, 2015; Ferreira et al., 2020; Hermanson et al., 2022). The morphology seen in *Toxochelys* spp. and *Nichollsemys baieri* suggests that their head and neck musculature may be more similar to outgroups than to extant chelonioids. As a consequence, it seems likely that these early stem chelonioids could still retract their necks, which is indicated by the deep emarginations (e.g., Hermanson et al., 2022; Werneburg, 2015). We tested this by including *Nichollsemys baieri* into the 3D landmark dataset of Hermanson et al. (2022), which can assess the probability of neck retraction capacity for fossils based on an extant training dataset. The phylogenetic Flexible Discriminant Analysis returned a very high posterior probability for the presence of neck retraction, both using the complete set of landmarks (PP = 0.87; Fig. 15B) and the reduced version, not including the posterodorsal emargination region (PP = 0.95) (see methods). The accuracy rate of predicting neck retraction in extant turtles was 1: all turtles were correctly classified according to their neck retraction capacity, regardless of the landmark scheme used, which increases the confidence with which even relatively low posterior probabilities can be interpreted (Fabbri et al., 2022). Ancestral reconstruction of the probability of neck retraction of Pan-Chelonioidea is 0.61 (Fig. 15C), and thus lower than the ancestral value for Cryptodira (PP = 0.84). This likely acknowledges the theoretical possibility that neck retraction of *Nichollsemys baieri* could have evolved independently, and the presence of non-neck retracting turtles in the sister clade of chelydroids (i.e., *Macrochelys temminckii*). Nevertheless, posterior probabilities above 0.5 in the light of the extremely high accuracy of predictions can be interpreted to indicate the presence of neck retraction. Thus, our analysis places high confidence in the presence of neck retraction for *Nichollsemys baieri*, but also suggest that the trait was ancestrally present in Pan-Chelonioidea, albeit with lower certainty. This can hopefully be further tested by the evaluation of cervical morphology in future fossil finds. Simultaneously, derived features related to feeding biomechanics of chelonioids (e.g., reduced upper temporal emargination, robust symphysis) are absent in *Toxochelys* spp. and *Nichollsemys baieri*, suggesting feeding behavior that is less reliant on strong bite forces (e.g., Jones et al., 2012).

Among the features that *Nichollsemys baieri* shares with *Toxochelys* spp., the presence of a splenial is particularly noteworthy. Splenials are generally absent in cryptodires (e.g., Dryden, 1988; Evers et al., 2022b; Gaffney, 1979), but they occur in *Toxochelys* spp. (Supplementary File 1: Fig. S3; contra Matzke, 2009). Current phylogenetic hypotheses thus suggest a secondary reappearance of the splenial (or, the evolution of a non-homologous neomorphic element). Interestingly, splenials are present in protostegids (e.g., Evers et al., 2019a), which may be one feature that associates them with stem chelonioids in our phylogeny. A similarly unexpected feature of *Toxochelys* spp. is the presence of the nasal bone. Matzke (2009) observed the presence of nasals in several specimens of *Toxochelys latiremis* (specifically, AMNH 1496 and AMNH 5118). However, the shapes of the nasals in both specimens appear to be quite different in the provided dorsal figures. Our re-investigations of both specimens confirm the presence of symmetrical nasals by well-defined sutures to the adjacent prefrontals and maxillae. The apparent differences in shape results from the anterior collapsing of the nasals ventrally into the nasal capsule in AMNH 5118, given the nasals a smaller appearance in dorsal view than actually true. In both specimens, the nasals have a median contact to one another, a more or less straight suture posteriorly to the prefrontal, and a short ventrolateral process that contacts the maxilla. Like splenials, nasals are plesiomorphically present in turtles and also present in protostegids, but lost in cryptodires (e.g., Gaffney, 1990; Joyce, 2007; Miller et al., 2023), including in *Nichollsemys baieri* and crown chelonioids.

*Toxochelys* spp. and *Nichollsemys baieri* both share with more derived pan-chelonioids a reduction in the size of the antrum postoticum. The resulting smaller middle ear volume is consistent with an aquatic ecology (Foth et al., 2019), corroborating postcranial evidence for *Toxochelys* spp. that indicates these taxa have marine adaptations (e.g., Evers et al., 2019a; Hay, 1908; Hirayama, 1995; Wieland, 1905; Zangerl, 1953a, 1980).

Although turtles generally have relatively thick (i.e., large internal diameter) endosseous semicircular canals, this is hypertrophied in protostegids and crown chelonioids (Evers et al., 2019b), which show much thicker canals than their sister lineage (chelydroids; Evers et al., 2019b). TMP 1997.99.1 also exhibits extremely thick and bulky semicircular canals (Fig. 10), which are seemingly thicker than in protostegids and even crown chelonioids, with the exception of *Dermochelys coriacea* (Evers et al., 2019b). Thick endosseous semicircular canals have been observed and described in a range of secondarily marine reptiles (e.g., Neenan et al., 2017; Schwab et al., 2020), which has been interpreted as a potential aquatic adaptation. For example, among sauropterygians, more pelagic species exhibit thicker semicircular canals (Neenan et al., 2017). Although a high degree of aquatic adaptation cannot be the only reason for increasing semicircular canal thickness, as thick semicircular canals also occur in highly terrestrial tortoises among turtles (Evers & Al Iawati, 2024; Evers et al., 2019b), the thick canals of TMP 1997.99.1 indicate that *Nichollsemys baieri* shares morphological innovations of the labyrinth endocast with sea turtles to the exclusion of chelydrids as the sister lineage. On the other hand, *Nichollsemys baieri* also exhibits plesiomorphic inner ear features, as the fenestra ovalis has a lateral orientation (Fig. 10A) as in most turtles, including chelydrids and protostegids, and not the derived condition of crown chelonioids, in which the fenestra ovalis is posteroventrally oriented (e.g., Evers et al., 2019b). The functional significance of both features is currently unclear (e.g., Schwab et al., 2020). Increases in the cross-sectional area of the semicircular duct reduces the response time of the vestibular system (Rabbitt et al., 2004), and it is conceivable how tunings of this could be useful for various clades, including marine animals which move in a three-dimensional space as opposed to a terrestrial plane. However, a direct functional relationship of increased semicircular canal thickness with labyrinth response time would require a corresponding increase in membranous duct thickness. So far, comparisons of membranous and endosseous labyrinths of turtles suggest that thick bony canals of sea turtles do not correlate with enlarged duct diameters, but instead accommodate an increased perilymphatic space (Evers et al., 2022a). The “soft tissue” preservation of TMP 1997.99.1 provides additional insights into this question. Interestingly, the posterior semicircular duct of TMP 1997.99.1 has a large internal diameter that fills an unexpectedly large proportion of the bony canal (Fig. 10B, E). The cross-section of the duct of TMP 1997.99.1 is nearly circular (as expected for a semicircular duct), with the major axis being 2.16 mm and the minor being 1.98 mm, resulting in an area of 3.36 mm^2^. The semicircular canal is both larger and more eccentric than the semicircular duct, with the major axis being 2.93 mm and the minor axis being 2.34 mm, resulting in an area of 5.37 mm^2^. Thus, the posterior semicircular duct fills 62% of the cross-sectional space of the posterior semicircular canal of TMP 1997.99.1. This is insofar unexpected that previous measurements for the extant *Trachemys scripta* found the duct to fill only 16–26% of the cross-sectional canal area (Evers et al., 2019b), and that visualizations of duct-to-canal systems also suggest strong size discrepancies between ducts and canals for other extant turtles (Ferreira et al., 2022), including for sea turtles (Evers et al., 2022a). This raises three possible explanations: (1) the semicircular ducts in TMP 1997.99.1 are simply thicker than in any sampled extant turtle, (2) the semicircular duct diameter is taphonomically somehow increased, or (3) the measured structure is not a semicircular duct in the first place, but rather a taphonomic artifact. It is hard to reconcile the second scenario with the geometrical duct course that fits the expectations for a semicircular duct, including the peripheral suspension from within the canal wall and the near-circular outline in cross-section. Regardless of the functional interpretation of the potential “soft tissue” preservation, the inner ear morphology of *Nichollsemys baieri* displays both plesiomorphic (fenestra ovalis orientation) and derived (thick endosseous semicircular canals) features that underscore its transitional morphology.

Some of the most important morphological innovations that *Nichollsemys baieri* shares with later chelonioid sea turtles, but which are absent in *Toxochelys* spp., is the modification of the rostrum basisphenoidale of the parabasisphenoid to a rod-like process and the absence of an epipterygoid. Absences of epipterygoids are sometimes hard to establish, because the bone may have indistinct sutures with surrounding bones. However, our high-resolution CT data provide excellent internal views on this region, such that we are confident that the absence of an epipterygoid is a true feature, as in extant chelonioids. It is hard to conceive if this change had any particular functional implications. The loss and appearance of the epipterygoid is fairly common among turtles (Brinkman, 2003; Gaffney, 1979, 1982; Meylan, 1987; Miller et al., 2023) but seems to have no functional consequences (Miller et al., 2023). The change in rostrum morphology, although distinct, is also hard to interpret in a functional perspective. Nevertheless, the documented changes with regard to the morphology of *Toxochelys* spp. provides evidence that these taxa are distinct species, and that *Nichollsemys baieri* is slightly more derived, which is consistent with its younger stratigraphic occurrence.

### Campanian sea turtle diversity

Besides *Toxochelys* spp. and *Nichollsemys baieri*, there are additional taxa of marine turtles known from both within the Western Interior Seaway, and nearby seas during the Late Cretaceous. This includes *Porthochelys laticeps* as another potential ‘toxochelyid’ (Hay, 1905; Williston, 1903; Zangerl, 1953a), but also more enigmatic forms that are likely more derived and potentially belong to the crown group of chelonioids. For instance, ctenochelyids are coeval with *Toxochelys* spp. (e.g., Brinkman et al., 2006; Hay, 1905; Matzke, 2007; Zangerl, 1953a) and have recently been interpreted to be stem cheloniids (Gentry, 2016, 2018; Gentry et al., 2018). *Mexichelys coahuilaensis* from the Late Campanian of Mexico with its broad secondary palate is potentially related to the “*Euclastes*-group” (Brinkman et al., 2009; Parham & Pyenson, 2010), which likely also are crown chelonioids (e.g., Gentry et al., 2019; Parham, 2005). Regardless of the exact phylogenetic position of any of these turtles, they document a relatively large taxonomic and morphologic diversity of pan-chelonioids during this period. This could either mean that pan-chelonioids quickly diversified within the Western Interior Seaway shortly after their origin, but it could alternatively also indicate that the common ancestry of all of these turtles extends further into the past than currently documented by fossils and predicted by molecular divergence time estimates. The pan-chelonioid diversity could potentially even include protostegids, which display large size variation (e.g., *Calcarichelys gemma*; *Archelon ischyros*; Farina et al., 2023; Hooks, 1998; Wieland, 1896; Zangerl, 1953b). Since protostegids already have a near cosmopolitan distribution early during their evolutionary appearance (Cadena & Parham, 2015; Collins, 1970; Evers et al., 2019a; Gaffney, 1981; Hirayama, 1995, 1998; Kear & Lee, 2006; Lydekker, 1889), this hypothesis is also hard to reconcile with biogeographic origination scenarios (Evers & Benson, 2019; Gentry et al., 2019), which place the divergence of the Americhelydian subgroups of chelonioids and chelydroids into North America (Joyce et al., 2013, 2016; Pereira et al., 2017).

### Future directions and outstanding problems

Although our phylogenetic results about the position of *Nichollsemys baieri* as an early stem chelonioid that is more derived than *Toxochelys* spp. makes morphological sense, some of our other topological results are highly dubious beyond the already discussed controversy about protostegids. This includes the composition of dermochelyids, specifically the inclusion of the Eocene *Erquelinnesia gosseleti* and the Oligocene putative stem cheloniids *Procolpochelys charlestonensis* (Weems & Brown, 2017) and *Oligochelone rupeliensis* (Zangerl, 1980). Another unexpected and unlikely result is the recovery of Eocene putative stem cheloniids (e.g., *Puppigerus camperi*; Moody, 1974; Evers & Benson, 2019) as part of an extended chelonioid stem lineage. These topological results are found even with minimal modifications (i.e., 28 character states out of 34.532 cells) to the baseline matrix of Joyce et al. (2021a), suggesting that it is the relative positional change of *Nichollsemys baieri* that causes these topological differences. Interestingly, variations in character ordering (i.e., applying ordering or not) as well as weighting (i.e., equal weighting or implied weighting with a concavity constant of K = 12) have almost no bearing on these relationships.

Given that these different treatments of the dataset seem to converge on similar results, we inspected the underlying dataset for potential explanations of our unexpected phylogenetic results. Because *Nichollsemys baieri* is only known from cranial material, we computed the amount of missing cranial data for our dataset. For the unexpected ‘dermochelyids’ *Erquelinnesia gosseleti* (62% missing cranial data), *Procolpochelys charlestonensis* (34.8%) and *Oligochelone rupeliensis* (100%), high amounts of unknown character states could explain their dubious topological positions. Besides the absence of skulls altogether (i.e., *Oligochelone rupeliensis*), these high numbers can be explained by the absence of cranial CT scans as well (i.e., *Erquelinnesia gosseleti* and *Procolpochelys charlestonensis*), as many cranial characteristics are internal and cannot be easily determined even from good external descriptions, such as those available for *Procolpochelys charlestonensis* (Weems & Brown, 2017) and *Erquelinnesia gosseleti* (Zangerl, 1971). The stemward positions for Eocene putative cheloniids in our topology are insofar curious as these taxa are characterized among our matrix by scoring fully segmented skull models (Evers & Benson, 2019). Thus, their skull anatomy is exceptionally well characterized within the matrix, resulting in low values of missing data (*Puppigerus camperi*: 8.6%; *Eochelone brabantica*: 8.0%, *Argillochelys cuneiceps*: 8.6%; similar to the level of *Nichollsemys baieri*: 9.6%). For comparison, the lowest values among chelonioids are two of the extant taxa with 7.5% missing cranial data (*Natator depressus*, *Eretmochelys imbricata*). There are also a few morphological characters that can likely explain the position of Eocene putative stem cheloniids. For example, *Puppigerus camperi* (IRSNB R0073) and *Eochelone brabantica* (IRSNB R0061) share with *Toxochelys* spp. (Zangerl, 1953a) to the exclusion of extant chelonioids a relatively strongly ossified plastron with xiphiplastra that contact one another over their entire midline length and hypoplastra that are at least partially in tight contact with one another posterior to the central plastral fontanelle. Also, the anterior surangular process that is present extant chelonioids (including *Dermochelys coriacea*, Evers et al., 2022b) is absent in *Puppigerus camperi* (Moody, 1974) and *Argillochelys antiqua* (NHMUK R38955), and incipient in *Eochelone brabantica* (IRSNB R0001). Characters like these provide clues about the potential explanation for the topology.

For future studies, we envision several avenues that can bring forward the study of chelonioid phylogeny. On one hand, taxon sampling needs to be further expanded, particularly to include a broad sample of taxa from the “*Euclastes*”-group (e.g., *Pacifichelys urbinai*, *Eulcastes wielandi*, *Euclastes acutirostris*, *Mexichelys coahuilaensis*; Fastovsky, 1985; Brinkman, 2009; Brinkman et al., 2009; Jalil et al., 2009; Parham & Pyenson, 2010) to test their position among pan-chelonioids. A broader sample of taxa that are quite likely closely related to extant cheloniids (e.g., *Ashelychelys plameri*, *Carolinochelys wilsoni*; Weems & Sanders, 2014; Weems & Brown, 2017) can further establish the variation among pan-cheloniids. A broader sample of ctenochelyids (e.g., *Prionochelys matutina*, *Peritresius ornatus*; Baird, 1964; Gentry, 2018) could help to understanding if the cheloniid stem truly extends into the Cretaceous. Simultaneously, character definitions and scorings need to be further scrutinized. Also, our work highlights that cranial CT scans are important to evaluate the morphology of externally well-known skulls such as the one from *Nichollsemys baieri*. Lastly, specific topological arrangements are often unexpected because of their unrealistic biogeographic or stratigraphic implications. Thus, methods that include stratigraphic or biogeographic data as part of the phylogenetic reconstruction may be a good alternative to ‘standard’ parsimony analysis. Bayesian fossilized-birth death models (Heath et al., 2014) that specifically include stratigraphic information have only recently been used for turtle datasets (e.g., Evers et al., 2023; Farina et al., 2023; Holley et al., 2020; Sterli et al., 2018), but find higher stratigraphic fit for topological results (e.g., Evers et al., 2023).

### Supplementary Information

Additional file 1.

Additional file 2.

Additional file 3.

Additional file 4.

Additional file 5.

Additional file 6.

Additional file 7.

Additional file 8.

Additional file 9.

Additional file 10.

## Data Availability

All data generated or analyzed during this study are available. The CT scan of specimen TMP 1997.99.1 is available on MorphoSource (https://www.morphosource.org/concern/media/000600370), and the 3D files are also available under this repository (https://www.morphosource.org/projects/000600361). All additional data are provided within the manuscript of as supplementary information files.
