# Supplementary material for: New insights into the early morphological evolution of sea turtles by re-investigation of Nichollsemys baieri, a three-dimensionally preserved fossil stem chelonioid from the Campanian of Alberta, Canada
Source: Swiss J Palaeontol. 2024 Jul 12;143(1):27. doi: 10.1186/s13358-024-00323-8 (PMC11245440; doi:10.1186/s13358-024-00323-8)
Supplement: Supplementary file 1 — Additional file 1. [file 13358_2024_323_MOESM1_ESM.docx]

Supplementary information for Menon et al. 2014: **A three-dimensionally preserved fossil stem chelonioid from the Campanian of Alberta, Canada, provides insights into the evolutionary origin of sea turtles**

**Content**

1. Character list

2. Supplementary figures

**1. Character list**

**CRANIUM**

**Character 1.** Nasals: 0 = present; 1 = absent.

**Character 2.** Nasal, medial contact of nasals: 0 = nasals contact one another medially along their entire length; 1 = medial contact of nasals partially or fully hindered by long an- terior frontal process.

**Character 3**. Nasal, medial contact of nasals: 0 = nasals contact one another medially along their entire length; 1 = medial contact of nasals partially or fully hindered by long anterior frontal process.

**Character 4.** Prefrontals, medial contact of prefrontals on the dorsal skull surface: 0 = absent; 1 = present, absence of contact between the nasal or apertura narium externa and the frontal.

**Character 5.** Prefrontal, prefrontal-vomer contact: 0 = present; 1 = absent.

**Character 6**. Prefrontal, prefrontal-palatine contact: 0 = present; 1 = absent.

**Character 7.** Prefrontal, dorsal prefrontal exposure: 0 = present, large; 1 = reduced; 2 = absent or near absent.

**Character 8.** Prefrontal, cranial scutes on the prefrontal: 0 = one pair; 1= two pairs or more.

**Character 9.** Prefrontal, sculpturing: 0 = heavily sculptured; 1 = sculpturing absent.

**Character 10**: Prefrontal, preorbital bulge formed between the prefrontal and premaxilla: 0 = absent; 1 = present.

**Character 11.** Lacrimal: 0 = present; 1 = absent. JY1 & Sterli & de la Fuente (2013: ch 9, Lacrimal A).

**Character 12.** Frontal, frontal contribution to orbit: 0 = absent, contact between prefrontal and postorbital; 1 = present.

**Character 13**. Frontals, both frontals medially fused: 0 = absent; 1 = present.

**Character 14.** Frontal, direction of the orbits in dorsal view of the skull: 0 = laterally facing, with a very narrow to almost complete absent dorsal exposure of the maxilla and jugal; 1 = dorsolateral facing, with portions of the maxilla and jugal dorsally exposed.

**Character 15.** Frontals, development of crista cranii: 0 = crista cranii on ventral surface of frontals very shallow, sulcus olfactorius developed is a low trough; 1 = crista cranii developed as moderately deep parasagittal ridges on the ventral surface of each frontal, forming a ventrally open, median trough (the sulcus olfactorius) that extends from the anterior margin of the cavum cranii posteriorly to the fissura ethmoidalis anteriorly; 2 = crista cranii very deep anteriorly, forming extended processes that meet along the midline of the cranium and are sutured to one another, forming an ossified olfactory canal.

**Character 16.** Parietal, parietal-squamosal contact: 0 = present, upper temporal emargination absent or poorly developed; 1 = absent, upper temporal emargination well developed.

**Character 17.** Parietal, posterodorsal margin of the temporal fossa roofed by an overhanging process of the skull roof: 0 = absent; 1 = present.

**Character 18.** Parietal, contribution to the processus trochlearis oticum: 0 = absent; 1 = present.

**Character 19**. Parietals, foramen stapedio-temporalis: 0 = absent or weak, foramen stapedio- temporale concealed in dorsal view; 1 = moderate foramen stapedio-temporale, partial exposition of the processes trochlearis in dorsal view; 2 = strong, entire exposition of the processus trochlearis in dorsal view.

**Character 20.** Parietal, pineal foramen located medially between parietals: 0 = absent; 1 = present.

**Character 21.** Parietal, processus inferior parietalis: 0 = weak or absent, parietal does not contact the pterygoid, epipterygoid, and/or palatine; 1 = present and well developed, the parietal contacts the pterygoid, epipterygoid, and/or palatine.

**Character 22.** Parietal, closure of foramen nervi trigemini and the length of the anterior extension of the lateral braincase wall: 0 = foramen nervi trigemini anteriorly open, anterior extension of lateral braincase wall absent; 1 = foramen nervi trigemini anteriorly closed, processus inferior parietalis only produces a narrow strut anterior to the foramen nervi trigemini, usually absence of contact with palatine; 2 = foramen nerivi trigemini anteriorly closed, processus inferior parietalis produces an ex- tended process anterior to the foramen nervi trigemini, contact with palatine commonly present.

**Character 23.** Parietal, posterior ramus of processus inferior parietalis forming the posterior margin of the trigeminal foramen: 0 = absent; 1 = present.

**Character 24.** Posterior ramus of processus inferior parietalis of the parietal: 0 = short; 1 = long, excludes the prootic from the trigeminal foramen. This character is scored inapplicable for taxa that lack the process altogether.

**Character 25.** Parietal, ridge on lateral surface of processus inferior parietalis: 0 = absent; 1 = present, a ridge between the ventral surface of the parietal and the lateral surface of the descending process marks the border between the temporal and orbital fossae.

**Character 26.** Jugal, jugal-squamosal contact: 0 = present; 1 = absent.

**Character 27.** Jugal, jugal participation in the margin of the upper temporal emargination: 0 = absent; 1 = present, upper temporal emargination extensive.

**Character 28.** Jugal, medial process of jugal ventral to orbit: 0 = weakly developed or absent, jugal contacts only the maxilla; 1 = present and well developed, jugal contacts the maxilla as well as the palatine and/or pterygoid.

**Character 29.** Jugal, contact with the palatine: 0 = absent; 1 = present. This character is scored as inapplicable when the jugal lacks a medial process (ch 27.0).

**Character 30**. Jugal, contact with the pterygoid: 0 = absent; 1 = present. This character is scored as inapplicable when the jugal lacks a medial process (ch 27.0).

**Character 31.** Jugal, jugal-parietal contact: 0 = absent; 1 = present.

**Character 32.** Quadratojugal: 0 = present; 1 = absent.

**Character 33**. Quadratojugal, quadratojugal-maxilla contact: 0 = absent; 1 = present, jugal does not contribute to lower temporal emargination.

**Character 34.** Quadratojugal, quadratojugal-squamosal contact below the cavum tympani: 0 = absent; 1 = present.

**Character 35.** Quadratojugal, lower temporal emargination: 0 = weak to no emargination, the margin of the lower temporal emargination is formed by the quadratojugal or quadratojugal and jugal; 1 = moderate emargination, the margin of the lower temporal emargination is principally formed by the quadratojugal and jugal, but the maxilla is included in the anterior section of the margin and/or the quadrate is included in the posterior section of the margin; 2 = large emargination, the postorbital and/or squamosal and parietal are included in the margin of the lower temporal emargination.

**Character 36.** Squamosal, squamosal-postorbital contact: 0 = present; 1 = absent.

**Character 37**. Squamosal, squamosal-supraoccipital contact: 0 = absent; 1 = present.

**Character 38.** Squamosal, posterolateral protuberances developing horns: 0 = absent; 1 = present.

**Character 39.** Squamosal, very long posterior process, formed exclusively by the squamosal and protruding beyond condyles occipitalis: 0 = absent; 1 = present.

**Character 40.** Squamosal, squamosal-quadrate contact: 0 = tightly sutured; 1 = wide open.

**Character 41.** Squamosal, posterodorsal margin of cavum tympanum: 0 = the squamosal forms the posterodorsal margin of the cavum tympanum; 1 = the squamosal is excluded from the posterodorsal margin of the cavum tympanum.

**Character 42.** Postorbital, postorbital-palatine contact: 0 = absent; 1 = present, foramen palatinum posterius situated posterior to the orbital wall.

**Character 43.** Postorbital, contact with the quadratojugal: 0 = present; 1 = absent.

**Character 44.** Postorbital, postorbital-maxilla contact preventing the jugal from entering the orbital margin: 0 = absent; 1 = present.

**Character 45.** Postorbital, dorsal margin of orbit: 0 = continuously and concavely curved margin between frontals and jugal; 1 = frontal margin relatively narrow, with lateral bulge of postorbital.

**Character 46**. Supratemporal: 0 = present; 1 = absent.

**Character 47.** Teeth in premaxilla, maxilla, and dentary: 0 = present; 1 = absent.

**Character 48.** Premaxilla, subdivision of the apertura narium externa by an internarial process of the premaxilla: 0 = present; 1 = absent.

**Character 49.** Premaxilla, fusion of premaxillae: 0 = absent; 1 = present.

**Character 50.** Premaxilla, foramen praepalatinum: 0 = absent; 1 = present.

**Character 51.** Premaxilla, foramen intermaxillaris: 0 = absent; 1 = present.

**Character 52.** Premaxilla, exclusion of the premaxillae from the apertura narium externa: 0 = absent; 1 = present.

**Character 53.** Premaxilla, distinct, median premaxillary hook along the labial margin of the premaxillae: 0 = absent; 1 = present.

**Character 54.**  Premaxilla, cusps developed on the labial ridge in conjunction with maxilla: 0 = absent; 1 = present.

**Character 55.** Palatine, contribution to the anterior extension of the lateral braincase wall: 0 = absent; 1 = present.

**Character 56.** Palatine, contribution to the upper triturating surface: 0 = absent or less than 30% of the total width of the triturating surface; 1 = present, at least 30% or more of the total width of the triturating surface.

**Character 57**. Palatine, secondary palate: 0 = absent; 1 = present, complete separation of the narial cavity from the oral cavity.

**Character 58.** Palatine, vomer-palatine contact anterior to internal naris (apertura narium interna): 0 = absent; 1 = present.

**Character 59.** Maxilla, triturating surface definition: 0 = triturating surface with labial ridge only; 1 = triturating surface with labial and lingual ridge; 2 = triturating surface with labial, lingual, and accessory ridge(s).

**Character 60.** Maxilla, accessory ridge(s): 0 = accessory ridge(s) on maxilla present along the triturating surface; 1 = accessory ridge(s) only in some sectors of the triturating surface. Thic character is scored as inapplicable when no accessory ridges are present (ch 58.0 or ch 58.1).

**Character 61.** Maxilla, median contact between right and left maxilla on the palate: 0 = absent; 1 = present.

**Character 62.** Vomer, number of vomer(s): 0 = paired; 1 = single, but large; 2 = single and greatly reduced or absent.

**Character 63.** Vomer, vomer-pterygoid contact in palatal view: 0 = present; 1 = absent, medial contact of palatines present.

**Character 64.** Vomer, vomerine and palatine teeth: 0 = present; 1 = absent.

**Character 65.** Vomer, vomer-premaxilla contact in ventral view: 0 = present; 1 = absent.

**Character 66.** Vomer, ventral median crest: 0 = absent, ventral surface of vomer is smooth; 1 = present, shallow ridge extends along the ventral surface posterior to ventral process of the vomer, ridge becomes shallower posteriorly; 2 = narrow and tall ventral crest present all along the vomer.

**Character 67.** Vomer, shape of the palate roof: 0 = flat; 1 = domed.

**Character 68.** Vomer, shape of anterior end contacting the maxillae and praemaxillae: 0 = flat, near horizontal contact with maxillae; 1 = the anterior end of the vomer is anteroventrally directed and laterally expanded; 2 = the anterior end of the vomer is ventrally expanded to form a horizontal footplate with a flat ventral surface.

**Character 69.** Vomer, contribution to the upper triturating surface: 0 = absent, triturating surface narrow to absent; 1 = present.

**Character 70.** Vomer, median trough on dorsal surface posterior to sulcus vomeri: 0 = absent, dorsal surface of vomer flat or transversely convex; 1 = present, dorsal surface bears a median trough that extends posteriorly from the sulcus vomeri.

**Character 71***.* Foramen orbito-nasale: 0 = formed as true foramen that is surrounded by bone from all sides; 1 = foramen orbito-nasale is not completely surrounded by bone and coalescent with the passage between the fossa orbitalis and the fossa nasalis.

**Character 72.** Foramen orbito-nasale, contribution of vomer: 0 = absent; 1 = present.

**Character 73***.* Foramen orbito-nasale, contribution of the maxilla: 0 = absent; 1 = present.

**Character 74.** Quadrate, precolumellar fossa: 0 = absent; 1 = present.

**Character 75.** Quadrate, development of the cavum tympani: 0 = shallow, but not developed anteroposteriorly; 1 = shallow, but anteroposteriorly developed; 2 = deep and anteroposteriorly developed.

**Character 76***.* Quadrate, anterior margin of the cavum tympanum: 0 = formed entirely by the quadrate; 1 = formed by the quadratojugal, which overlaps the lateral surface of the quadrate, reaching the anterior margin of the cavum tympanum.

**Character 77.** Quadrate, antrum postoticum: 0 = absent; 1 = incipient, the antrum postoticum is completely formed within quadrate (irrespective of the elements involved in forming the margin of the cavum tympanum); 2 = antrum postoticum fully developed, and extending posterodorsally into the squamosal (i.e. there is a large posterodorsal fenestra in the quadrate that leads to a pocket within the squamosal). This character is scored as inapplicable for turtles without a cavum tympanum.

**Character 78.** Quadrate, incisura columellae auris: 0 = absent, stapes extends posteroventrall to quadrate body; 1 = present, but open posteroventrally; 2 = present and closed, but only enclosing the stapes; 3 = present and closed, enclosing stapes and the Eustachian tube.

**Character 79***.* Quadrate, formation of incisura columella auris: 0 = formed exclusively by quadrate; 1 = formed by quadrate and squamosal and/or quadratojugal.

**Character 80.** Quadrate, processus trochlearis oticum: 0 = absent; 1 = present, very reduced; 2 = present, large forming a well defined musculatory facet.

**Character 81.** Quadrate, contribution to the musculatory facet of the processus trochlearis oticum: 0 = extensive contribution; 1 = small contribution, facet formed principally by the protic and/or parietal. This character is scored as inapplicable when a processus trochlearis oticum is absent (ch 79.0).

**Character 82***.* Quadrate, width of processus trochlearis oticum: 0 = the otic process spans all the mediolateral space between the braincase wall and the lateral surface of the skull; 1 = the otic process is limited to the medial part of the otic chamber, and there is a deep recess laterally. This character is scored as inapplicable when a processus trochlearis oticum is absent (ch 79.0).

**Character 83.** Quadrate, quadrate-basisphenoid contact: 0 = absent; 1 = present.

**Character 84***.* Quadrate, infolding ridge on the posterior surface of the quadrate ventral to the incisura columella auris: 0 = absent; 1 = present.

**Character 85.** Quadrate, direction of cranial articular process: 0 = ventrolaterally directed; 1 = with strong posterior inclination.

**Character 86***.* Posterior quadrate fossa: 0 = absent; 1 = present.

**Character 87.** Stapes, lateral articulation: 0 = stapes articulates with medial surface of the quadrate, quadrate has stapedial pit; 1 = stapes articulates with tympanic membrane, pit on medial surface of quadrate is absent.

**Character 88.** Epipterygoid: 0 = present; 1 = absent.

**Character 89.** Epipterygoid, shape: 0 = rod-like element; 1 = laminar element. This character is scored as inapplicable if an epipterygoid is absent (ch 87.1).

**Character 90.** Pterygoid, pterygoid teeth: 0 = present; 1 = absent.

**Character 91.** Pterygoid, basipterygoid process and basipterygoid articulation: 0 = basipterygoid process present with a movable basiptergoid articulation; 1 = basipterygoid process present with a sutured basipterygoid articulation; 2 = basipterygoid process absent and sutured basipterygoid articulation.

**Character 92.** Pterygoid, pterygoid-basioccipital contact: 0 = absent; 1 = present.

**Character 93**: Basioccipital, anterolateral edge of basioccipital with knob-like processes fitting into sockets on the posterior processes of the pterygoids: 0 = absent; 1 = present.

**Character 94.** Pterygoid, processus trochelaris pterygoideus: 0 = absent; 1 = present.

**Character 95.** Pterygoid, foramen palatinum posterius: 0 = present; 1 = present, but open laterally; 2 = absent.

**Character 96.** Pterygoid, medial contact of pterygoid: 0 = present, pterygoids in a very long medial contact with one another, longer than the basisphenoid total length in midline; 1 = present, pterygoids in medial contact with one another, contact length equal or shorter than the basisphenoid total length in midline; 2 = absent, contact of the basisphenoid with the vomer and/or palatines present.

**Character 97.** Pterygoid, pterygoid contribution to foramen palatinum posterius: 0 = present; 1 = absent. This character is scored inapplicable when the foramen palatinum posterius is absent (ch. 66.0).

**Character 98.** Pterygoid, contact with the exoccipital: 0 = absent; 1 = present.

**Character 99**. Pterygoid, fossa podocnemidoidea or cavum pterygoidei: 0 = absent; 1 = present.

**Character 100.** Pterygoid, lateral margin: 0 = a processus pterygoideus externus is developed as a process that projects into the subtemporal fenestra; 1 = the lateral margin of the pterygoid is gently expanded laterally and/or expanded dorsoventrally; 2 = absent, i.e. the lateral margin of the pterygoid forms a straight or concave outline that forms the medial margin of the subtemporal fenestra. Scored inapplicable for pleurodires.

**Character 101.** Pterygoid, processus pterygoideus externus: 0 = forming an extensive process that contacts the maxilla anterolaterally at the posteromedial end of the triturating surface, is anteriorly sutured to the anterior palate, and has a posterior projection into the subtemporal fenestra; 1 = forming a large lateral wing that projects as a free process into the subtemporal fenestra; 2 = forming a pointed triangular process that projects laterally into the subtemporal fenestra. Scored inapplicable for taxa that lack a processus pterygoideus externus (i.e. ch 98.1 or 98.2)

**Character 102.** Pterygoid, vertical flange on anterolateral margin of the pterygoid: 0 = absent; 1 = present. Zhou *et al.* (2014) & Joyce (2007: ch 67) (Pterygoid I).

**Character 103.** Pterygoid, level of the position of the pterygoid respect to basisphenoid: 0 = both bones are at the same level on ventral surface; 1 = two different levels, creating a step between the two bones.

**Character 104.** Pterygoid, ventral median ridge: 0 = incipient to absent; 1 = present, ridge spans nearly the full length of the pterygoids, sometimes reaching the most posterior portion of the vomer. This character is scored as inapplicable for taxa in which the pterygoids lack a midline contact.

**Character 105.** Pterygoid, extending laterally almost reaching the mandibular condyle facet: 0 = absent; 1 = present, the pterygoid contacts the medial edge of the mandibular condyle when is seem in ventral view; 2 = present, the pterygoids extends not only laterally to reach the outline of the mandibular condyle facet, but also posteriorly far from the level of the condyles.

**Character 106.** Pterygoid, ventral ridge on the palatal surface lateral to skull midline. 0 = absent; 1 = present, each pterygoid has a parasagittal ridge on its ventral surface.

**Character 107***.* Pterygoid, extent of ventral ridge on the palatal surface lateral to skull midline: 0 = each ridge extends along most of the ventral surface of the pterygoid, from the anteromedial margin of the pterygoid fossa to the processus pterygoideus externus; 1 = each ridge extends only along the posterior part of the pterygoid, along the level of the parabasisphenoid. This character is scored inapplicable in turtles in which ventral pterygoid ridges are absent (ch 104.0).

**Character 108.** Pterygoid/Quadrate, flooring of cavum acustico-jugulare and recessus scalae typmani: 0 = absent; 1 = present, formed primarily by the posterior part of the pterygoid; 2 = present, produced by the ventral process of the quadrate or prootic or a posterolateral expansion of the parabasisphenoid.

**Character 109.** Pterygoid, posterior process: 0 = posterior process of pterygoid is absent, the cranioquadrate space or posterior foramen for the canalis cavernosus is not covered by the pterygoid; 1 = posterior process of pterygoid present but very short, process extends posteriorly to cover the posterior foramen for the canalis cavernosus (i.e. the modified cranioquadrate space), but the cavum-acustico jugulare remains largely exposed ventrally; 2 = posterior process of the pterygoid present and developed as an extensive sheet that projects posteriorly and covers large parts of the cavum acustico-jugulare.

**Character 110.** Pterygoid, development of a posteromedial wing covering partially to completely the basisphenoid and sometime the basioccipital too, seen in ventral view of the skull: 0 = absent; 1 = present.

**Character 111.** Pterygoid, pterygoid fossa: 0 = weakly developed; 1 = developed as a deep concavity between the articular process of the quadrate and the basicranium.

**Character 112.** Supraoccipital, crista supraoccipitalis: 0 = poorly developed; 1 = protruding significantly posterior to the foramen magnum.

**Character 113.** Supraoccipital, large supraoccipital exposure on dorsal skull roof: 0 = absent; 1 = present.

**Character 114.** Supraoccipital, horizontal crest in the crista supraoccipitalis: 0 = absent or poorly developed anteriorly; 1 = present, along the entire crista supraoccipitalis.

**Character 115.** Supraoccipital, fossa on the posterodorsal surface of the floor of the supratemporal fossa: 0 = absent; 1 = present, fossa is formed on the lateral surface of the suparoccipital, dorsal to the contact area between exoccipital, opisthotic, and supraoccipital.

**Character 116.** Exoccipital, medial contact of exoccipitals dorsal to foramen magnum: 0 = absent; 1 = present.

**Character 117.** Exoccipital, median contact of exoccipitals in the floor of the foramen magnum, excluding the basioccipital from the latter: 0 = absent; 1 = present.

**Character 118.** Foramen nervi hypoglossi (XII), ventral covering: 0 = exposed in ventral view; 1 = covered in ventral view by an extension of the pterygoid and the basioccipital; 2 = covered in ventral view an extension of the basioccipital; 3 = covered in ventral view by an expansion of the exoccipital and basioccipital.

**Character 119.** Exoccipital, foramina nervi hypoglossi: 0 = the foramina nervi hypoglossi exit the exoccipital on the occipital surface posteriorly to the margin of the fenestra postotica; 1 = at least one foramen nervi hypoglossi opens within the recessus scalae tympani anterior to margin of the fenestra postotica, and the others exit the exoccipital on the occipital surface; 2 = all foramina nervi hypoglossi open within the recessus scalae tympani anterior to the margin of the fenestra postotica.

**Character 120**. Basioccipital, morphology of the anteriormost part of the basioccipital: 0 = with two or one ventral tubercle; 1 = tubercle absent.

**Character 121**. Basioccipital, deep C-shaped concavity between basioccipital tubera: 0 = absent; 1 = present.

**Character 122.** Basioccipital, basal tubera: 0 = the basal tubera are completely formed by the basioccipital; 1 = the exoccipitals and the basioccipital form the basal tubera; 2 = the pterygoids and the basioccipital form the basal tubera; 3 = the basal tubera are formed by the exoccipitals, basioccipital and pterygoids.

**Character 123**. Prootic, dorsal exposure: 0 = large; 1 = very reduced or absent.

**Character 124.** Prootic, lateral semicircular canal enclosure by bone: 0 = canal only formed by bone of the opisthotic, the prootic portion of the canal is not ossified and is instead medially confluent with the recessus labyrinthicus prooticus; 1 = prootic and opisthotic both contribute to the formation of the lateral semicircular canal.

**Character 125.** Prootic, ventral process: 0 = ventral process is short and without extensive posterior contact with the pterygoid; 1 = ventral process is large, with a broad contact with the pterygoid along a posteriorly expanded footplate, forming parts of the floor of the inner ear cavity; 2 = ventral process is extensive, and forms parts of the floor of the basicranium so that it is visible on the ventral surface of the skull. This character is scored as inapplicable for taxa that lack an anatomically modern middle ear chamber.

**Character 126.** Prootic, unnamed foramen exiting into the subtemporal fossa from the canalis cavernosus: 0 = absent; 1 = present.

**Character 127.** Prootic/pterygoid, posteroventral elongation of the trigeminal foramen: 0 = absent, the trigeminal foramen is approximately circular or oval, but the ventral margin of the foramen is positioned above the level of the canalis/sulcus cavernosus; 1 = the trigeminal foramen is oval, anterodorsally-posteroventrally elongate and anteroventrally-posterodorsaly narrow, and the ventral margin is level with the canalis/sulcus cavernosus. This character is scored inapplicable for turtles without a trigeminal foramen (i.e. without a neomorphic secondary lateral wall of the braincase formed by the parietal and pterygoid).

**Character 128.** Prootic, recess on posterior surface of the element anterodorsolaterally to the fenestra ovalis: 0 = absent; 1 = present.

**Character 129.** Prootic, position of the geniculate ganglion and the split of the facial nerve into the hyomandibular and palatine branches: 0 = the geniculate ganglion is positioned with the canalis cavernosus; 1 = the geniculate ganglion is positioned within the facial nerve canal; 2 = the geniculate ganglion is positioned in the canalis carotici interni.

**Character 130.** Prootic/opisthotic, enclosure of fenestra ovalis: 0 = the fenestra ovalis is ventrally enclosed by the prootic and opisthotic; 1 = the prootic and opisthotic do not have a contact ventrally to the fenestra ovalis.

**Character 131.** Opisthotic, wide transverse occipital plane with depression for the nuchal musculature: 0 = absent; 1 = present.

**Character 132.** Opisthotic, processus interfenestralis: 0 = developed as a robust ridge that does not form a ventrally projecting process and does not reach closely to the floor of the basicranium; 1 = incipient, developed as a ventrally low and mediolaterally broad, robust structure that separates the cavum labyrinthicum anteriorly from an incipient recessus scalae tympani posteriorly; 2 = present, developed as a ventrally directed process that separates the cavum labyrinthicum anteriorly from the recessus scalae tympani posteriorly.

**Character 133.** Opisthotic, development of the processus interfenestralis: 0 = developed as a ventrally directed process that separates the cavum labyrinthicum anteriorly from the recessus scalae tympani posteriorly, the process is not expanded at its ventral end, and almost or just about reaches the floor of the basicranium, but a small gap (hiatus postlagenum) usually remains; 1 = developed as a ventrally directed process that separates the cavum labyrinthicum anteriorly from the recessus scalae tympani posteriorly, but the process has a horizontally expanded footplate at its ventral end that is sutured to elements of the basicranium; 2 = developed as a ventrally directed process that separates the cavum labyrinthicum anteriorly from the recessus scalae tympani posteriorly, and the ventral surface of the process is integrated into the basicranium to form parts of the ventral surface of the cranium. This character is scored as inapplicable if an anatomically modern processus interfenestralis is absent (i.e. ch 131.0 or 131.1).

**Character 134.** Opisthotic, posterior surface of paroccipital process: 0 = the occipital side of the paroccipital process is developed as a posteriorly exposed, broad, planar or gently concave surface; 1 = the occipital side of the paroccipital process is dorsoventrally flattened and forms a posterior ridge that traverses the paroccipital process mediolaterally; 2 = the paroccipital process has a dorsoventrally convex surface.

**Character 135**. Fenestra perilymphatica: 0 = large; 1 = reduced in size to that of a small foramen.

**Character 136.** Parabasisphenoid, paired pits on ventral surface of basisphenoid: 0 = absent; 1 = present.

**Character 137.** Parabasiphenoid, ventral surface: 0= flat to slightly convex, with posterior margin straight or slightly concave; 1= V-shaped crest, with posterior margin forming the basipterygoid process projected posterolaterally.

**Character 138.** Parabasiphenoid, rough surface between basisphenoid and basioccipital: 0 = absent; 1 = present.

**Character 139***.* Parabasisphenoid, posterolateral processes lapping onto the ventral surface of the basioccipital: 0 = absent; 1 = present.

**Character 140.** Parabasisphenoid, rostrum basisphenoidale: 0 = flat; 1 = flat base, but with trabeculae contact one another medially forming a short rod at the anterior end of the parabasisphenoid; 2 = singular median, rod-like, thick and rounded process.

**Character 141.** Parabasisphenoid, dorsum sellae: 0 = deep, i.e. the dorsal surface of parabasisphenoid between the clinoid processes is a transversely concave floor forming a trough between the posterior part of the dorsal surface of the parabasisphenoid, which is usually cup-like, and the anterior portion of the parabasisphenoid forming the rostrum basisphenoidale and sella turcica; 1 = low, i.e. the dorsum sellae is formed as a transverse ridge between the clinoid processes that projects anteriorly at a low angle from posterodorsal surface of the parabasisphenoid; 2 = high, i.e. a transverse ridge or wall of bone between the clinoid processes is present that projects dorsally at a high angle from the posteriorly positioned cup, separating the cup very clearly from of the anteriorly positioned rostrum basisphenoidale and sella turcica.

**Character 142.** Parabasisphenoid, anterior surface of dorsum sellae: 0 = anterior surface of the dorsum sellae is flat and smooth; 1 = vertical median ridge on anterior surface of dorsum sellae between the clinoid processes is present, ridge may have a small anterodorsal projection.

**Character 143.** Parabasisphenoid, retractor bulbi pits on anterolateral surface: 0 = absent; 1 = present.

**Character 144.** Parabasisphenoid, foramina anterius canalis carotici cerebralis: 0 = widely separated; 1 = close together; 2 = right and left cerebral arteries converge within the parabasisphenoid and run within a short joint canal, and exit anteriorly into the cavum cranii via a single foramen within the sella turcica.

**Character 145.** Parabasisphenoid, prootic foramen in primary lateral wall of the braincase: 0 = present, the clinoid process of the parabasisphenoid and parts of the prootic, an ossified pila antotica, the laterosphenoid, and/or parts of the parietal form a foramen medially to the cavum epiptericum; 1 = the prootic foramen is reduced, and the clinoid processes are free structures.

**Character 146.** Parabasisphenoid, crista tuberculi basalis developed on posterior part of dorsal surface: 0 = absent, parabasisphenoid dorsal surface is relatively flat or gently concavely excavated; 1 = present as median, dorsally high projecting tubercle or ridge.

**Character 147.** Carotid artery/Pterygoid, interpterygoid vacuity: 0 = large opening, triangular in shape; 1 = reduced to an interpterygoid slit; 2 = entirely closed, no foramina posterius canalis carotici palatinum present. Scored inapplicable for taxa with a bony canal for the palatine artery (ch 146.1)

**Character 148.** Embedding of the palatine division of carotid artery: 0 = the palatine artery is not encased in a bony canal (artery enters the skull through the interpterygoid cavuity, interpterygoid slit, or possibly other skull openings); 1 = the palatine artery is encased in a bony canal (irrespective of the exposure or embedding of the internal carotid artery split).

**Character 149.** Embedding of internal carotid artery and its bifurcation: 0 = Internal carotid arterial system is not embedded by bone, a foramen posterius canalis carotici is absent, and the split into palatine and cerebral artery occurs extracranially; 1 = internal carotid arterial system is partially embedded, a foramen posterius canalis carotici interni is present, and the split into palatine and cerebral artery happens at the fenestra caroticus and is thus ventrally exposed; 2 = internal carotid arterial system is ventrally fully embedded by bone, a foramen posterius canalis carotici interni is present and the split into palatine and cerebral artery is ventrally covered by bone.

**Character 150.** Position of the foramen posterius canalis carotici interni (fpcci): 0 = the fpcci is located at the ventral surface of the skull in a position far anterior to the margin of the fenestra postotica; 1 = the fpcci is located at the posterior end of the skull, either on the ventral surface of the skull close to the margin of the fenestra postotica, or on the posterior surface of the skull at the ventral margin of the fenestra postotica. This character is scored inapplicable for taxa that lack a foramen posterius canalis carotici interni.

**Character 151.** Entry of the internal carotid artery into skull relative to cavum acustico-jugulare: 0 = internal carotid enters the skull ventrally to the level of the cavum acustico-jugulare; 1 = internal carotid artery enters the skull within the cavum acustico-jugulare, artery lies in dorsally open trough on dorsal surface of pterygoid and only becomes dorsally covered within the cavum acustico-jugulare.

**Character 152.** Dorsal exposure of the palatine artery and/or anterior parts of the internal carotid artery: 0 = absent, the split of the internal carotid artery is dorsally covered by bone, and the palatine artery exits the basicranium into the cavum cranii via the foramen anterius canalis carotici palatinum; 1 = the internal carotid artery exits the braincase anterodorsally into the sulcus cavernosus where it continues on the floor of the sulcus cavernosus (= sulcus caroticus of Gaffney 1979), and the split into cerebral and palatine arteries occurs within the sulcus cavernosus. The cerebral artery enters the parabasisphenoid medially, whereas the palatine artery continues in the sulcus cavernosus anteriorly without ever being embedded in a canal. This character is scored as inapplicable when the carotid split is ventrally exposed (i.e. ch 148.0 or 148.1).

**Character 153.** Formation of the foramen posterius canalis carotici interni, pterygoid involvement: 0 = present; 1 = absent.

**Character 154.** Formation of the foramen posterius canalis carotici interni, parabasisphenoid involvement: 0 = present; 1 = absent.

**Character 155**. Formation of the foramen posterius canalis carotici interni, quadrate involvement: 0 = present; 1 = absent.

**Character 156.** Formation of the foramen posterius canalis carotici interni, prootic involvement: 0 = present; 1 = absent.

**Character 157.** Hyomandibular branch of the facial nerve: 0 = contained within the canalis cavernosus; 1 = contained in a sulcus or separate canal paralleling the canalis cavernosus.

**Character 158.** Stapedial artery, foramen stapedio-temporale: 0 = present; 1 = absent.

**Character 159.** Stapedial artery, size of foramen stapedio-temporale: 0 = relatively large (the size of a large blood foramina, ≥5 mm diameter); 1 = significantly reduced in size (the size of a nerve foramina, ≤3 mm diameter. This character is scored as inapplicable for taxa without a foramen stepedio-temporale (i.e. ch 156.1).

**Character 160.** Stapedial artery, foramen stapedio-temporale location in the otic chamber: 0 = on dorsal part and pointing dorsally; 1 = on the anterior wall of the otic region, pointing anteriorly. This character is scored as inapplicable for taxa without a foramen stepedio-temporale (i.e. ch 156.1).

**Character 161.** Stapedial artery, formation of the foramen stapedio-temporale, contribution of the quadrate: 0 = absent; 1 = present. Scored inapplicable for taxa without a foramen stapedio-temporale. This character is scored as inapplicable for taxa without a foramen stepedio-temporale (i.e. ch 156.1).

**Character 162.** Stapedial artery, formation of the foramen stapedio-temporale, contribution of the prootic: 0 = absent; 1 = present. Scored inapplicable for taxa without a foramen stapedio-temporale. This character is scored as inapplicable for taxa without a foramen stepedio-temporale (i.e. ch 156.1).

**Character 163.** Stapedial artery, formation of the foramen stapedio-temporale, contribution of the opisthotic: 0 = absent; 1 = present. Scored inapplicable for taxa without a foramen stapedio-temporale. This character is scored as inapplicable for taxa without a foramen stepedio-temporale (i.e. ch 156.1).

**Character 164.** Stapedial artery, formation of the foramen stapedio-temporale, contribution of the supraoccipital: 0 = absent; 1 = present. Scored inapplicable for taxa without a foramen stapedio-temporale. This character is scored as inapplicable for taxa without a foramen stepedio-temporale (i.e. ch 156.1).

**Character 165.** Foramen jugulare posterius, relationship with the fenestra postotica: 0 = separate from fenestra postotica; 1 = coalescent with fenestra postotica.

**Character 166.** Foramen jugulare posterius, formation of lateral bar separating foramen from fenestra postotica: 0 = formed by pterygoid; 1 = formed by opisthotic and/or exoccipital. The character is scored inapplicable for taxa in which the foramen jugulare posterius is coalescent with the fenestra postotica (ch. 163.1).

**Character 167.** Recessus scalae tympani: 0 = almost nonexistent, not surrounded by bone; 1 = well developed.

**Character 168.** Cranial scutes, scute D meeting in midline: 0 = absent; 1 = present.

**Character 169.** Cranial scutes, scute X much smaller than scute D: 0 = absent; 1 = present.

**Character 170.** Cranial scutes, scute X partially separates scutes G: 0 = absent; 1 = present.

**Character 171.** Cranial scutes, scutes A, B, and C forming a continuous posterolateral shelf: 0 = absent; 1 = present.

**Character 172.** Cranial scutes, scute F: 0 = formed by several scutes; 1 = formed by a single scute.

**Character 173.** Cranial scutes, scute J: 0 = formed by several scutes; 1 = formed by a single scute.

**Character 174.** Dentary, medial contact of dentaries: 0 = fused; 1 = open suture.

**Character 175.** Dentary, width triturating surface vs. jaw length: 0 = narrow triturating surface, symphysis less than 1/3 of jaw length; 1 = broad triturating surface, symphysis ≥1/3 jaw length.

**Character 176.** Dentary, symphyseal ridge: 0 = absent; 1 = present.

**Character 177.** Dentary, lingual (tomial) ridge: 0 = prominent; 1 = weak or absent.

**Character 178.** Dentary, size of foramen dentofaciale majus: 0 = small, size of a small vessel; 1 = enlarged, foramen is several mm in diameter.

**Character 179.** Dentary-Surangular arrangement: 0 = lack of a posterior expansion of dentary and anterior projection of surangular; 1 = posterior expansion of dentary present almost reaching the articular surface, covering the dorsal half of the surangular in lateral view, surangular with anterior projection.

**Character 180.** Surangular, with anteromedial process forming a vertical lamina that projects anteriorly into the fossa meckelii: 0 = absent; 1 = present.

**Character 181.** Coronoid, anteromedial process: 0 = absent; 1 = present.

**Character 182.** Coronoid, contribution to triturating surface: 0 = absent; 1 = present.

**Character 183.** Coronoid, notch on posterior margin of coronoid: 0 = absent; 1 = present.

**Character 184.** Coronoid, foramen at anterior end, leading from fossa meckelii into space between mandibular rami: 0 = absent; 1 = present.

**Character 185.** Coronoid process, principally formed by: 0 = coronoid; 1 = dentary; 2 = surangular.

**Character 186.** Coronoid process: 0 = relatively low, dorsally well rounded; 1 = relatively high, process is dorsally or posterodorsally pointed.

**Character 187.** Splenial: 0 = present; 1 = absent.

**POSTCRANIUM**

**Character 188.** Carapace, carapacial scutes: 0 = present; 1 = absent.

**Character 189.** Carapace, carapacial scutes: 0 = present, ful9y covering the carapace; 1 = reduced not fully covering the carapace.

**Character 190.** Carapace, continuous keel on costals: 0 = absent; 1 = present.

**Character 191.** Carapace, continuous keel on neurals: 0 = absent; 1 = present.

**Character 192**. Shell, sculpturing of dorsal surface (carapace) and ventral surface (plastron): 0 = absent, smooth to slightly rugose; 1 = present, development of striations, vermiculations, striations, or pitting.

**Character 193.** Shell, pattern of sculpturing of the dorsal surface (carapace) and ventral surface (plastron): 0 = parallel to radial striations; 1 = vermiculation; 2 = highly dense pattern of pitting combined with striations; 3 = dichotomic striations; 4 = spread pitting without marked striation pattern; 5 = granules (positive relief).

**Character 194.** Carapacial Sutures: 0 = carapacial elements finely sutured or the contact is smooth; 1 = carapacial sutures strongly serrated in adult stage.

**Character 195.** Nuchal, articulation of nuchal with neural spine of eighth cervical vertebra: 0 = articulation via a blunt facet is present; 1 = articulation along a blunt facet absent.

**Character 196.** Raised pedestal on the visceral surface of the nuchal for the articulation with the neural spine of the eighth cervical vertebra: 0 = absent; 1 = present.

**Character 197.** Nuchal, elongate costiform process: 0 = absent; 1 = present.

**Character 198.** Nuchal, length of costiform process: 0 = crosses peripheral 1; 1 = reaches peripherals 2 or 3. This character is scored inapplicable for taxa without a costiform process on the nuchal (ch. 195.0).

**Character 199.** Nuchal, length versus width: 0 = wider than long; 1 = longer than wide or as long as wide.

**Character 200.** Nuchal, posteriomedial fontanelles: 0 = absent; 1 = present.

**Character 201.** Neurals, neural formula 6>4<6<6<6<6: 0 = absent; 1 = present.

**Character 202.** Neurals, shape of neurals: 0 = very irregular in shape, wider than long or squared; 1 = regular, often perfectly hexagonal or pentagonal, longer than wide.

**Character 203.** Neurals: 0 = present; 1 = absent.

**Character 204.** Neurals, number of neurals: 0 = ten or more; 1 = nine or less.

**Character 205.** Peripheral Gutter: 0 = peripheral gutter absent of only anteriorly developed; 1 = peripheral gutter extensively developed along anterior and bridge peripherals.

**Character 206.** Peripherals,: 0 = present; 1 = absent.

**Character 207.** Peripherals, number of peripherals: 0 = more than 11 pairs of peripherals present; 1 = 11 pairs of peripherals present; 2 = 10 pairs of peripherals present. This character is scored as inapplicable when peripherals are absent (ch. 204.1).

**Character 208.** Peripherals, anterior peripherals incised by musk ducts: 0 = absent; 1 = present.

**Character 209.** Costals, medial contact of the first pair of costals: 0 = absent; 1 = present.

**Character 210.** Costals, medial contact of posterior costals: 0 = absent; 1 = present.

**Character 211.** Costals, number of costals involved in medial contact: 0 = medial contact of up to three posterior costals; 1 = medial contact of all costals. This character is scored as inapplicable for turtles without a medial contact of posterior costals.

**Character 212**: Costals, lateral ossification: 0 = all costals fully ossified laterally with strong sutural contact with peripherals, lack of dorsal exposure of distal end of costal ribs and absence of costo-peripheral fontanelles; 1 = lateral sutural contact contact between costals and peripherals absent in at least parts of the costo-peripherial series, resulting in the presence of costo-peripheral fontanelles and/or the exposure of the distal rib ends.

**Character 213**: Costals, position of costo-peripheral fontanelles and exposure of dorsal rib ends: 0 = limited to parts of the carapace; 1 = fontanelles and exposed rib ends present and retained in adults between all costals and along the anterior margin of the first costal. Scored as inapplicable for taxa that lack costo-peripheral fontanelles (character 212.0).

**Character 214**: Costal, fontanelle along anterior margin of costal 1: 0 = anterior margin of first costal positioned very close to nuchal and/or anteriormost peripherals, reducing the fontanelle to an anteroposteriorly narrow, slot-like opening; 1 = extensive fontanelle between first costal and anterior margin of carapace. Scored inapplicable for taxa without costo-peripheral fontanelles (character 212.0) or when costo-peripheral fontanelles are absent along the first costal.

**Character 215**: Posterior costals, shape: 0 = rectangular, much wider mediolaterally than long anteroposteriorly; 1 = square or hexagonal, as wide as long.

**Character 216.** Rib free peripherals: 0 = absent; 1 = present.

**Character 217.** Position of rib free peripherals: 0 = only present anterior and posterior to ribs; 1 = present between sixth and seventh ribs. This character is scored as inapplicable for taxa that lack rib free peripherals (ch. 211.0).

**Character 218.** Costals, alternative short and long ends in the lateral part of costals: 0 = absent; 1 = present.

**Character 219.** Costals, costal 9: 0 = present; 1 = absent.

**Character 220.** Costals, shape of Costal 3: 0 = tapering towards the lateral side of the shell or with parallel anterior and posterior borders; 1 = broadens towards the lateral side of the shell.

**Character 221.** Suprapygals: 0 = present; 1 = absent.

**Character 222.** Suprapygals, number of suprapygals: 0 = one; 1 = two; 2 = more than two.

**Character 223.** Suprapygals, size between suprapygal 1 and 2: 0 = suprapygal 1 smaller than suprapygal 2; 1 = suprapygal 1 larger.

**Character 224.** Cervical scutes: 0 = present; 1 = cervical scutes absent.

**Character 225.** Number of cervical scutes: 0 = more than one cervical scute present; 1 = one cervical scute present. This character is scored as inapplicable when cervical scutes are absent (ch. 219.1)

**Character 226.**  Pygal, posterior notch: 0 = present; 1 = absent.

**Character 227.** Supramarginals: 0 = present; 1 = absent.

**Character 228.** Supramarginals, separating marginal and pleurals: 0 = complete row present, fully separating marginals from pleurals; 1 = partial row present, incompletely separating marginals from pleurals. This character is scored as inapplicable for turtles in which supramarginals are absent.

**Character 229.** Vertebrals, shape of the verterbrals: 0 = vertebrals 2 to 4 signicantly broader than pleurals; 1 = vertebrals 2 to 4 as narrow as, or narrower than, pleurals.

**Character 230.** Vertebrals, position of vertebral 3-4 sulcus in taxa with five vertebrals: 0 = sulcus positioned on neural 6; 1 = sulcus positioned on neural 5.

**Character 231.** Vertebrals, vertebral 3-4 sulcus with a wide posteriorly oriented medial embayment: 0 = absent; 1 = present.

**Character 232.** Vertebrals, verebral 1: 0 = vertebral 1 does not enter anterior margin of carapace; 1 = enters anterior margin.

**Character 233.** Marginals, marginal scutes overlap onto costals: 0 = absent, marginals restricted to peripherals; 1 = present.

**Character 234.** Pleurals, at least one pair of additional pleural scutes located laterally of vertebral scute 1, with anterior contact with cervical scute: 0 = absent; 1 = present.

**Character 235.** Plastron, connection between carapace and plastron: 0 = osseous; 1 = ligamentous.

**Character 236.** Plastron, central plastral fontanelle: 0 = absent; 1 = present.

**Character 237 .** Plastron, posterior plastral fontanelle, posterior plastral fontanelle between the xiphiplastra and/or the hypoplastra: 0 = absent in adult stage; 1 = retained in adult stage.

**Character 238.** Plastron, plastral kinesis: 0 = absent, scutes sulci and bony sutures do not overlap; 1 = present, scutes sulci coincide with epiplastral-hyoplastral contact.

**Character 239.** Plastron, plastral kinesis: 0 = between hyoplastron and hypoplastron; 1 = between hyoplastron and epiplastron- entoplastron.

**Character 240.** Plastron, hyo-hypoplastra contact: 0 = contact between hyo-hyoplastra absent or reduced; 1 = extensive contact between hyo-hyoplastra (even for those taxa with plastral kinesis).

**Character 241**: Plastron, hyo-hypoplastra serrations: 0 = serrations on the lateral and medial margins absent or weakly developed; 1 = strong serrations present along the surfaces that face other bones, but serrations are absent along the margin of the central fontanelle and the lateral contact area of hyo- and hypoplastro; 2 = strong serrations along all margins but the anterolateral margin of the hyoplastra and the posterlateral margin of the hypoplastra present, giving these elements a ‘star-shaped’ appearance.

**Character 242.** Axillar and inguinal notches: 0 = deep U or V-shaped axillar and inguinal notches; 1 = very shallow axillar and inguinal notches, and long lateral edges.

**Character 243.** Entoplastron: 0 = present; 1 = absent.

**Character 244.** Entoplastron, anterior entoplastral process: 0= present, medial contact of epiplastra absent; 1 = absent, medial contact of epiplastra present.

**Character 245.** Entoplastron, distinct posterolateral process: 0 = present; 1 = absent.

**Character 246:** Entoplastron, shape of the entoplastron: 0 = ‘dagger-shaped’, with dorsoventrally thick anterior end and long posterior process that extends along the dorsal surface of the plastron and sometimes reaches the mesoplastra; 1 = plate like and diamond-shaped or hexagonal in ventral view, with all margins of subequal length; 2 = T-shaped or triangular, i.e. entoplastron has a mediolaterally expanded anterior end and a progressively narrowing posterior process; 3 = strap like and V-shaped, with posterolateral processes diverging from the midline of the plastron.

**Character 247:** Entepiplastron: 0 = absent, entoplastron and epiplastra are separate elements; 1 = present, entoplastron is fused with epiplastra, resulting in a laterally extremely expanded entepiplastron wings.

**Character 248.** Entoplastron, suture with hyoplastra: 0 = tightly sutured; 1 = lightly sutured to almost absent contact between both.

**Character 249**: Epiplastra, shape: 0 = epiplastra squarish in shape and forming parts of the anterior plastral lobe; 1 = epiplastra elongate, become narrower posteriorly along the anterolateral margin of the hyoplastron, and with gently convex lateral margin; 2 = epiplastra are laterally strongy expanded to a wing-like shape; 3 = epiplastra rod-like and anteriorly as narrow as posteriorly, with concave lateral margin.

**Character 250.** Epiplastra, very thick anterior lip in dorsal view: 0 = present; 1 = absent.

**Character 251.** Hyoplastra, contacts of axillary buttresses: 0 = absent to slightly contacting peripherals only; 1 = peripherals and costal 1.

**Character 252.** Hyoplastra, axillary buttresses: 0 = present; 1 = ossified axillary buttresses absent.

**Character 253.** Hyoplastra, termination of axillary buttresses: 0 = terminates on peripheral 1 or 2; 1 = terminates on peripheral 3; 2 = terminates on peripheral 4 or 5 level. This character is scored as inapplicable for turtles without axillary buttresses (ch. 247.1).

**Character 254.** Mesoplastron: 0 = present; 1 = absent.

**Character 255.** Number of mesoplastra: 0 = two; 1 = one. This character is scored as inapplicable when mesoplastra are absent (ch. 249.1).

**Character 256.** Mesoplastron, medial contact of mesoplastra: 0=present, or virtually present when a central plastral fonta- nelle is present, absence of contact between hyoplastron and hypoplastron; 1 = absent, partial contact between hyoplastron and hypoplastron present.

**Character 257.** Hypoplastra, contacts of inguinal buttresses: 0 = absent to slightly contacting peripherals; 1 = peripheral and costal 5; 2 = peripheral, costals 5 and 6; 3 = peripherals and costal 4.

**Character 258.** Hypoplastra, termination of inguinal buttresses: 0 = peripheral 8; 1 = peripheral 7; 2 = peripheral 6.

**Character 259.** Xiphiplastra, distinct anal notch: 0 = absent; 1 = present.

**Character 260:** Xiphiplastra, shape of xiphiplastra: 0 = triangular, trapezoidal, or rectangular plate-like element; 1 = anteroposterior elongate rods.

**Character 261**: Xiphiplastra, articulation with hypoplastron: 0 = the xiphiplastra articulate with the hypoplastra along an anteriorly facing margin, forming a mediolaterally broad suture; 1 = the xiphiplastra have an elongate anterolateral process articulating along the posterolateral margin of the hypoplastron, resulting in an oblique suture, and the hypoplastra extend posteriorly along the anteromedial margin of the xiphiplastra.

**Character 262**: Xiphiplastra, posteriorly in contact with one another, often sutured along the midline and forming a plastral lobe: 0 = present; 1 = absent.

**Character 263.** Plastral scutes: 0 = present; 1 = absent.

**Character 264.** Plastral scutes, midline sulcus: 0 = straight; 1 = distinctly sinuous, at least for part of its length.

**Character 265.** Gular, number of gulars: 0 = one pair of scutes; 1 = only one scute.

**Character 266.** Extragulars: 0 = present; 1 = absent.

**Character 267.** Extagulars, medial contact: 0 = absent; 1 = present, contacting one another anterior to gular(s); 2 = present, contacting one another posterior to gular(s).

**Character 268.** Extragulars, anterior plastral tuberosities: 0 = present; 1 = absent.

**Character 269.** Extragulars, restricted to epiplastra: 0 = present; 1 = absent, extragulars reach the entoplastron.

**Character 270.** Intergulars: 0 = absent; 1 = present.

**Character 271.** Humerals, number of pairs: 0 = one pair present; 1 = two pairs present, subdivided by a plastral hinge.

**Character 272.** Humerals, humero-pectoral sulcus: 0 = restricted to hyoplastra; 1 = crossing the posterior portion of ento- plastron.

**Character 273.** Pectorals: 0 = present; 1 = absent.

**Character 274.** Pectorals, antero-posteriorly developed: 0 = present; 1 = absent, very short antero-posterior development.

**Character 275.** Abdominals: 0 = present; 1 = absent.

**Character 276.** Abdominals, medial contact to one another: 0 = present; 1 = absent. This character is scored as inapplicable for turtles that lack abdominals.

**Character 277.** Anals: 0 = only cover parts of the xiphiplastra; 1 = overlap anteromedially onto the hypoplastra.

**Character 278.** Inframarginals: 0 = present; 1 = absent.

**Character 279.** Number of inframarginals: 0 = more than two pair present, plastral scales do not contact marginals; 1 = two pair present (axillaries and inguinals), limited contact between plastral scales and marginals present. This character is scored as inapplicable when inframarginals are absent (ch. 271.1).

**Character 280.** Cervical ribs: 0 = large cervical ribs present; 1 = cervical ribs reduced or absent.

**Character 281.** Cervicals, position of the transverse processes: 0 = middle of the centrum; 1 = anterior end of the centrum.

**Character 282.** Cervicals, posterior cervicals with strongly developed ventral keels: 0 = absent or slightly developed in all vertebrae; 1 = present, more developed on posterior vertebrae.

**Character 283.** Cervicals, cervical 8 centrum significantly shorter than cervical 7: 0 = absent; 1 = present.

**Character 284.** Cervicals, triangular diapophyses: 0 = absent; 1 = present.

**Character 285.** Cervicals, central articulations of cervical vertebrae: 0 = articulations not formed, cervical vertebrae am- phicoelous or platycoelous; 1 = articulations formed, cervical vertebrae procoelous or opisthocoelous.

**Character 286.** Cervicals, articulation between cervical 8 and dorsal vertebrae 1: 0 = 8 (dorsal 1; 1 = 8) dorsal 1; 2 = vertebrae articulate along zygapophyses only.

**Character 287.** Cervicals, biconvex cervical vertebrae in the middle of the neck: 0 = absent; 1 = present.

**Character 288.** Cervicals, biconvex cervical vertebra in the middle of the neck: 0 = cervical 2; 1 = cervical 3; 2 = cervical 4; 3 = cervical 5.

**Character 289.** Cervicals, biconcave cervical vertebrae: 0 = absent; 1 = present.

**Character 290.** Cervicals, double articulation between cervical 5 and 6: 0 = absent; 1 = present.

**Character 291.** Cervicals, double articulation between cervical 6 and 7: 0 = absent; 1 = present.

**Character 292.** Cervicals, central articulation between cervical 6 and 7: 0 = cervical 6 concave ( cervical 7 convex; 1 = platycoelous, cervical 6 II cervical 7.

**Character 293.** Cervicals, double articulation between cervical 7 and 8: 0 = absent; 1 = present.

**Character 294.** Cervicals, height versus length of centra and neural arch: 0 = total height of centra and neural arch longer than the anteroposterior length of the cervical centra; 1 = total height of centra and neural arch much shorter than the anteroposterior length of the cervical centra.

**Character 295.** Cervicals, modification of neural arch on cervical 8: 0 = neural arch without modificiation of postzygapophyses; 1 = neural arch with postzygapophyses pointing anteroventrally.

**Character 296.** Cervicals, postzygapophyses united in midline: 0 = absent; 1 = present.

**Character 297.** Cervicals, ventral process on cervical 8: 0 = absent; 1 = present, well developed (as tall or taller than the height of the centrum).

**Character 298.** Cervicals, shape of central articulation of cervicals 7 and 8: 0 = as high as wide; 1 = much wider than high.

**Character 299.** Ribs, length of first dorsal rib: 0 = long, extends full length of first costal and may even contact peripherals distally; 1 = intermediate, in contact with well-developed anterior bridge buttresses; 2 = intermediate to short, extends less than halfway across first costal.

**Character 300.** Ribs, contact of dorsal ribs 9 and 10 with costals: 0 = present; 1 = absent.

**Character 301.** Dorsal rib 10: 0 = long, spanning full length of costals and contacting peripherals distally; 1 = short, not span- ning father distally than pelvis.

**Character 302.** Dorsals, anterior articulation of the first dorsal centrum: 0 = faces at most slightly anteroventrally; 1 = faces strongly anteroventrally.

**Character 303.** Caudals, tail club: 0 = present; 1 = absent.

**Character 304.** Caudals, anterior caudal centra: 0 = amphicoelous; 1 = procoelous or platycoelous; 2 = opisthocoelous.

**Character 305.** Caudals, posterior caudal centra: 0 = amphicoelous; 1 = procoelous or platycoelous: 2 = opisthocoelous.

**Character 306.** Caudals, chevrons: 0 = present on nearly all caudal vertebrae: 1 = absent, or only poorly developed, along the posterior caudal vertebrae.

**Character 307.** Caudals, tail ring: 0 = absent; 1 = present.

**Character 308.** Scapula, anterodorsal ridge of acromion: 0 = present; 1 = absent.

**Character 309.** Scapula, ventral ridge of acromion: 0 = present; 1 = absent developed proximally near glenoid.

**Character 310.** Scapula, horizontal ridge of acromion: 0 = well-developed, coracoid foramen present; 1 = reduced, only developed along distal portion of acromion.

**Character 311.** Scapula, glenoid neck on scapula: 0 = absent; 1 = present.

**Character 312.** Scapula, lamina between the dorsal process of the scapula and the acromion: 0 = well developed; 1 = reduced; 2 = absent.

**Character 313.** Scapula, internal angle between acromion process and scapular process ≥110°: 0 = absent; 1 = present.

**Character 314.** Coracoid, coracoid vs humerus length: 0 = shorter than humerus; 1 = at least as long as humerus.

**Character 315.** Coracoid, foramen: 0 = present; 1 = absent.

**Character 316.** Cleithrum: 0 = present; 1 = absent.

**Character 317.** Cleithrum, contact with carapace: 0 = present; 1 = osseous contact with carapace absent.

**Character 318.** Pelvis, pelvis-shell attachment: 0 = pelvis-shell attachment by ligaments; 1 = pelvis attached by strong sutural contact of the ischium and pubis with the plastron, and illium with the carapace.

**Character 319.** Pelvis, thyroid fenestra: 0 = coalescent; 1 = two separated fenestra completely or partially separated.

**Character 320.** Ilium, elongated iliac neck: 0 = absent; 1 = present.

**Character 321.** Ilium, iliac scar: 0 = extends from costals onto the peripherals and pygal; 1 = positioned on costals only.

**Character 322.** Ilium, shape of the ilium articular site on the visceral surface of the carapace: 0 = narrow and pointed poste- riorly; 1 = oval.

**Character 323.** Ilium, posterior notch in acetabulum: 0 = absent; 1 = present.

**Character 324.** Ilium, thelial process: 0 = absent; 1 = present.

**Character 325.** Pubis, lateral process: 0 = small, poorly developed, columnar; 1 = well developed and flat.

**Character 326.** Pubis, epipubis process: 0 = osseus or calcified; 1 = cartilaginous or absent.

**Character 327.** Ischium, ischial contacts with plastron: 0 = contact via a large central tubercle; 1 = contact via two separate ischial processes.

**Character 328.** Ischium, lateral process of ischium or metischial process: 0 = absent; 1 = present.

**Character 329.** Hypoischium: 0 = present; 1 = absent.

**Character 330.** Humerus, ectepicondylar foramen: 0 = in a channel; 1 = only a groove.

**Character 331.** Humerus, proximal articular surface of humerus: 0 = with shoulder on preaxial side, upturned; 1 = without shoulder, not upturned.

**Character 332.** Humerus, lateral process of humerus: 0 = abuts caput humeri; 1 = slightly separated from caput humeri; 2 = located distal to caput humeri but along proximal end of shaft; 3 = located at middle of humeral shaft.

**Character 333.** Humerus, prominent anterior projection of lateral process: 0 = absent; 1 = present.

**Character 334**: Humerus, distal articulation: 0 = articular surface forms distinct trochlea; 1 = rounded epiphyseal surface without clearly defined articulation facets.

**Character 335.** Humerus, length of the humerus versus the width of the proximal end: 0 = two times or less the width of the proximal end: 1 = more than two times the width of the proximal end.

**Character 336.** Humerus, scar for Muscle latissimus dorsi and Muscle teres major: 0 = located anterior to humeral shaft; 1 =

located at middle of shaft.

**Character 337.** Humerus, humerus length vs femur length: 0 = shorter than femur; 1 = longer than femur.

**Character 338.** Ulna, contact with radius through rugosity and ridge: 0 = absent; 1 = present.

**Character 339.** Radius, curves towards anterior: 0 = absent; 1 = present.

**Character 340.** Manus, phalangeal formula of the manus: 0 = most digits with two shortenened phalanges: 1 = most digits with three elongated phalanges.

**Character 341.** Manus, rigid articulations in 1^st^ and 2^nd^ digit: 0 = absent; 1 = present.

**Character 342.** Manus, rigid articulations in 3^rd^ to 5^th^ digit: 0 = absent; 1 = present.

**Character 343.** Manus, flippers: 0 = absent; 1 = short flippers present; 2 = elongate flippers present. Joyce (2007: ch 134, Manus C).

**Character 344.** Ulnare, size of the ulnare vs the intermedium: 0 = smaller than intermedium: 1 = nearly as large as intermedium; 2 = much larger than intermedium.

**Character 345**: Size of proximal carpals vs. distal carpals: 0 = proximal carpals are of similar size with respect to distal carpals; 1 = proximal carpals are much larger than distal carpals.

**Character 346**: Relative lengths of manual phalanges on the 3^rd^ and 4^th^ digit: 0 = the 1^st^ phalanx is longer than or equally long as the 2^nd^ phalanx; 1 = the 2^nd^ phalanx is longer than the 1^st^ phalanx. This character is scored as inapplicable when the manus digits only have two phalanges (i.e. the second phalanx is an ungual).

**Character 347**: 3^rd^ phalanx on 5^th^ manual digit: 0 = absent; 1 = present.

**Character 348 (modified from Evers et al. 219: ch.348)**: Relactive lengths of central digits in the manus: 0 = 4^th^ digit longer than 3^rd^ digit; 1 = 3^rd^ digit longer than fourth digit. This character is scored as inapplicable when the 3^rd^ and 4^th^ digits are equally long.

**Character 349.** Pes, number of digits: 0 = five; 1 = four.

**Character 350.** Manus and Pes, flattening of carpals and tarsal elements: 0 = absent; 1 = present.

**Character 351.** Manus and Pes, hyperphalangy manus digits 4 and 5, pes digit 4: 0 = absent; 1 = present.

**Character 352**: Femur, femoral trochanters: 0 = distinct, and separated from one another; 1 = connected by a ridge.

**Character 353**: Femur, intertrochanteric ridge: 0 = ridge is low and concave, creating a notch between the major and minor trachenter; 1 = ridge is high and obliterates intertrochanteric notch, and the proximal surface of the trochanters and their connecting ridge forms a continuous surface. This character is scored as inapplicable when an intertrochanteric ridge is absent (character 352.0)

**Character 354**: Femur, connection between femoral head surface and the major trochanter: 0 = the femoral head and major trochanter have distinct proximal surfaces separated by a deep notch; 1 = the femoral head surface slopes toward the major trochanter and forms a continuous proximal surface with it.

**Character 355.** Tibia, tibial pit for pubotibialis and flexor tibialis internus muscles: 0 = absent; 1 = present.

**Character 356 (new character).** Radius, relative placement to ulna within forearm: 0 = posterior to ulna; 1 = anterior to ulna, resulting in a constant hyperextension of the ellbow.

**2. Supplementary figures**


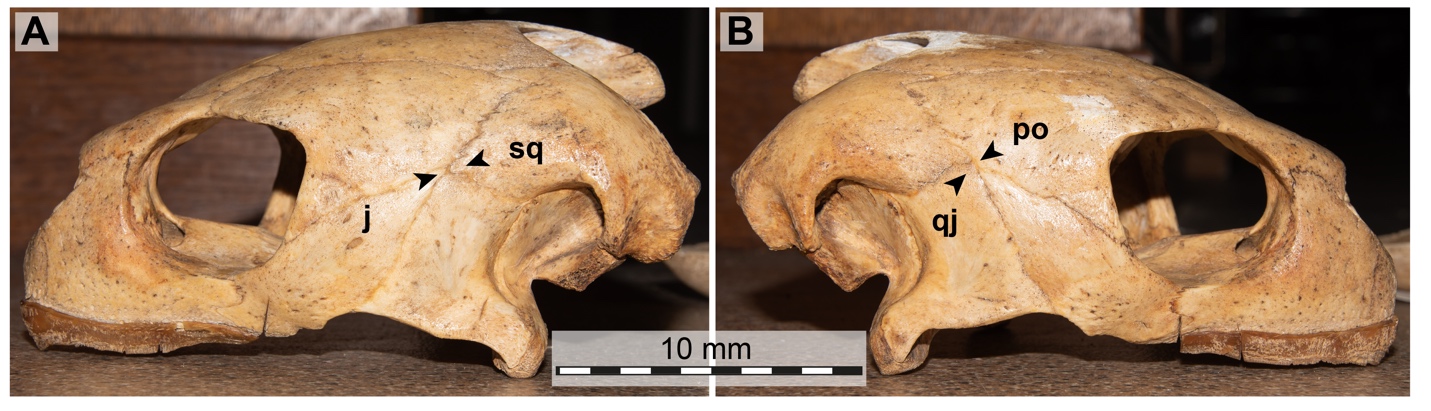


**Figure S1.** Photographs of *Caretta caretta* (EMV_2004_3_22). **A** left lateral side with unusual contact between jugal and squamosal. **B** right lateral side with regular morphology and contact between quadratojugal and postorbital. *j* jugal; *qj* quadratojugal; *po* postorbital; *sq* squamosal.

**
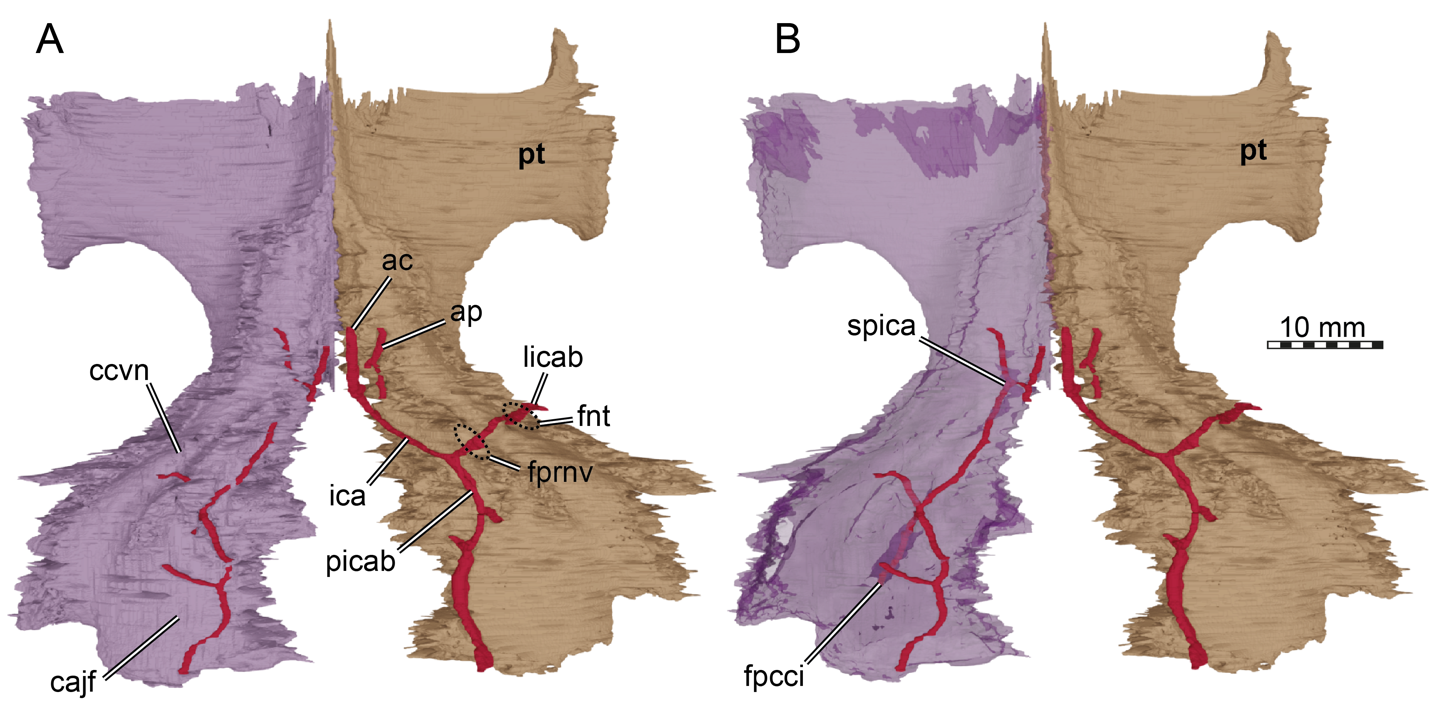
**

**Figure S2.** Potential arterial soft tissues of TMP 97.99.1. 3D renderings of the pterygoids in dorsal view, with arterial soft tissues segmented in red. A, pterygoids solid. B, pterygoids transparent. Abbreviations: ac, cerebral artery; ap, palatine artery; cajf, floor of cavum acustico-jugulare; ccnv, canalis cavernosus; fnt, foramen nerv trigemini; fpcci, foramen posterius canalis carotici interni; fprnv, foramen pro ramo nervi vidiani; ica, internal carotid artery; licab, lateral branch of internal carotid artery; picab, posterior branch of internal carotid artery; pt, pterygoid; spica, split of internal carotid artery into palatine and cerebral arteries.


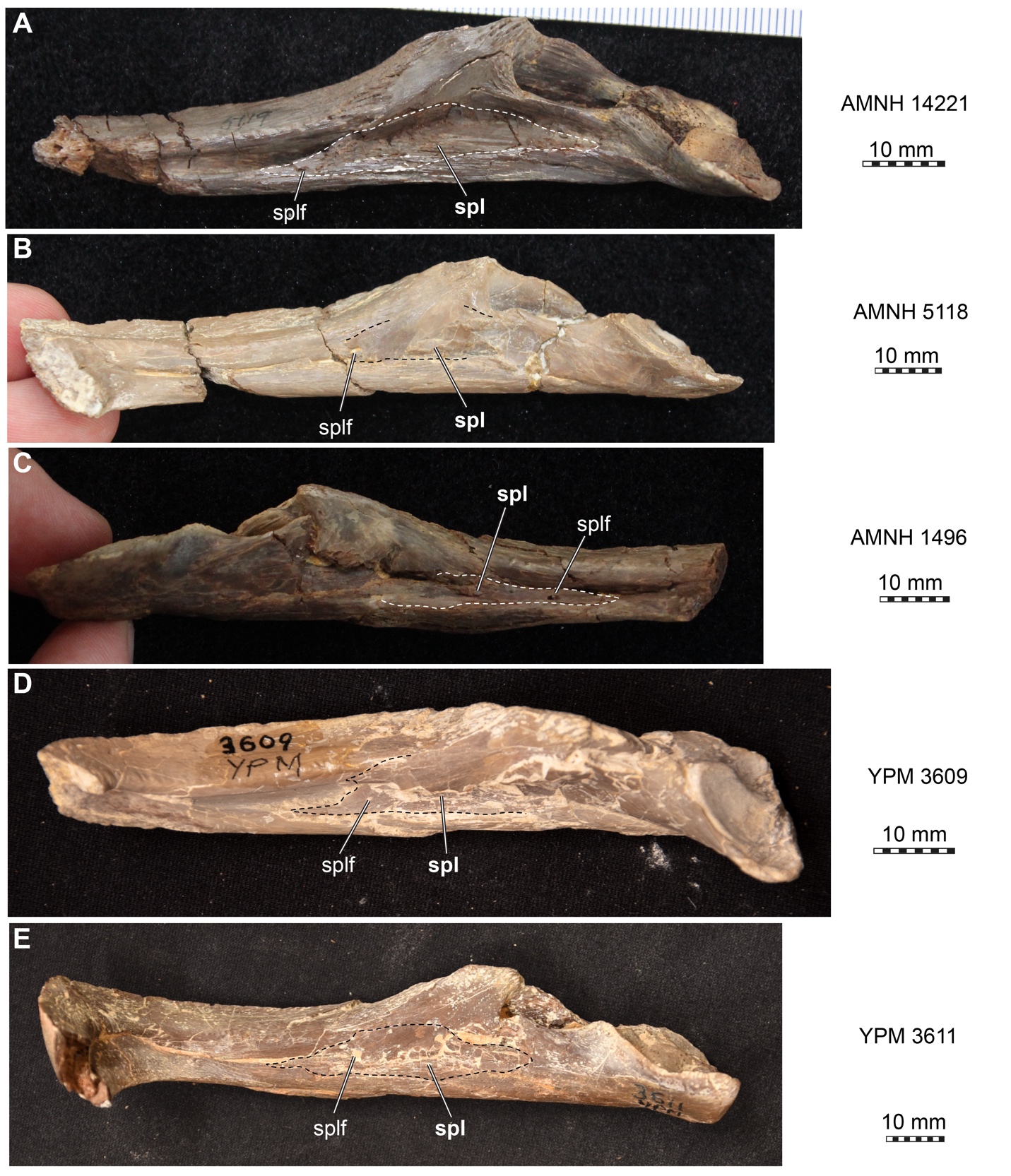


**Figure S3.** Evidence for the presence of splenials in *Toxochelys* spp. Approximate splenal shapes or sutures visible from photos are indicated with dashed lines. Abbreviations: AMNH, American Museum of Natural History; splf, splenial foramen; spl, splenial; YPM, Yale Peabody Museum.


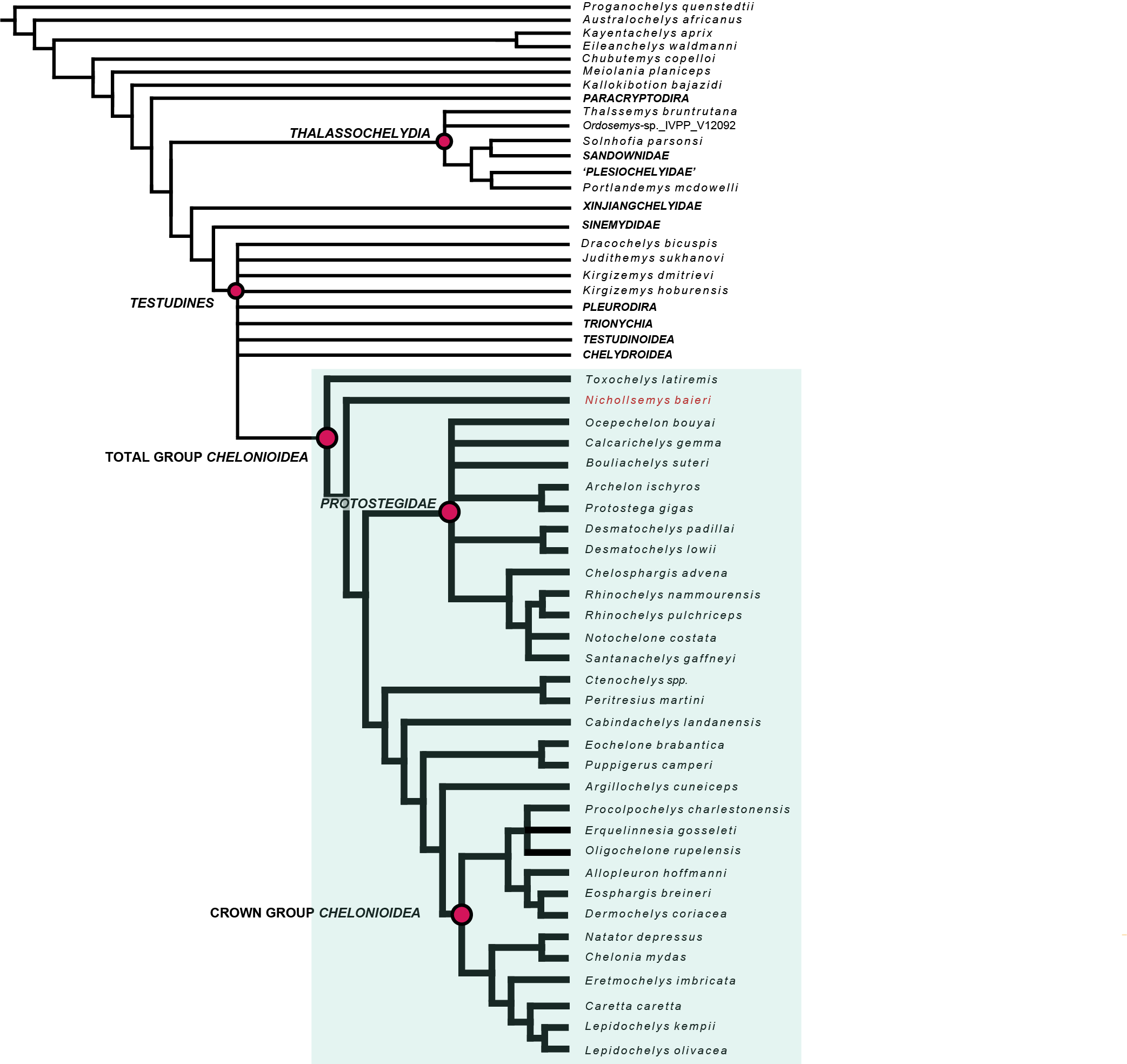


**Figure S4.** Strict consensus tree of 432 MPTs recovered through equally weighted parsimony analysis using unordered characters of the original Joyce et al. (2021) character-taxon matrix in which only the scorings for *Nichollsemys baieri* were updated. This tree, alongside the individual MPTs and a 50% majority rule consensus tree are available as Supplementary file S5.


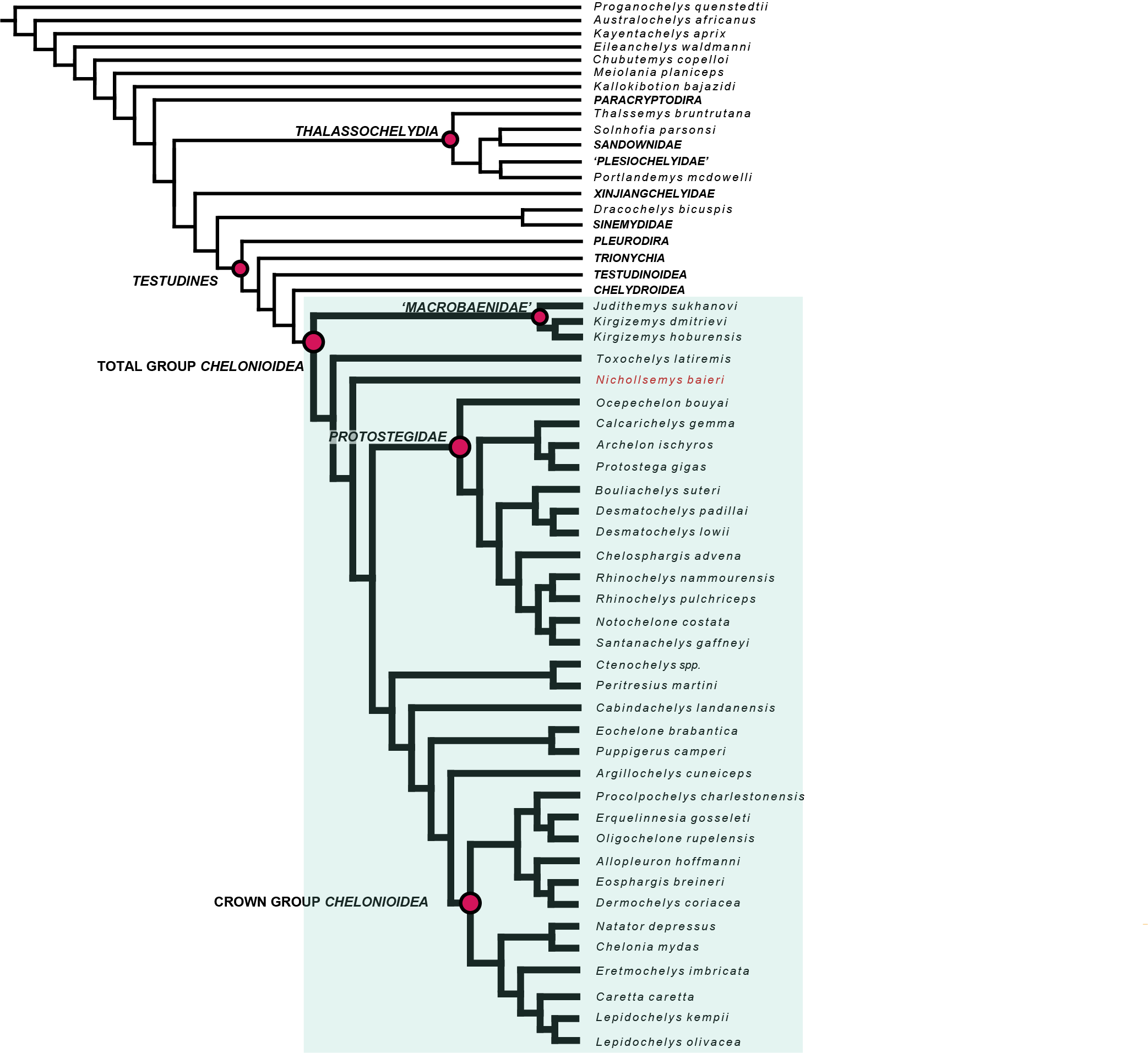


**Figure S5.** Strict consensus tree of 2 MPTs recovered through implied weighting (K=12) parsimony analysis using ordered characters and the fully revised character-taxon matrix. This tree, alongside the individual MPTs are available as Supplementary file S6.
